# Artificial intelligence for medication recognition and safety: a systematic review of detection, classification, OCR, and digital health applications

**DOI:** 10.3389/fdgth.2026.1963607

**Published:** 2026-10-15

**Authors:** A. Sai Sachin, J. Karthikeyan

**Affiliations:** School of Computer Science Engineering and Information Systems, Vellore Institute of Technology, Vellore, Tamil Nadu, India

**Keywords:** artificial intelligence, deep learning, digital health, medication recognition, medication safety, optical character recognition, pill classification, pill detection

## Abstract

**Introduction:**

Medication errors and adverse drug events remain causes of patient harm, underscoring the need for technologies supporting medication identification, verification, and medication safety. Artificial intelligence (AI), computer vision, optical character recognition (OCR), and digital health technologies are applied to medication recognition.

**Objective:**

To systematically review AI-based medication recognition systems and characterize recognition tasks, computational approaches, datasets, evaluation, validation, generalizability, deployment, applications, and emerging directions.

**Methods:**

A systematic review was conducted in accordance with PRISMA 2020. Scopus, Web of Science, IEEE Xplore, and PubMed/MEDLINE were searched for publications from 1 January 2016 to 4 September 2026, using two conceptual blocks covering medication recognition and AI methods. Of 895 retrieved records, 136 duplicates were removed, and 759 records underwent screening. After full-text assessment, 14 reports were excluded, and 91 studies were included. Title-and-abstract screening yielded 84.72% observed agreement (Cohen’s κ=0.527). For methodological assessment, both reviewers independently applied D1–D8 to the 91 included studies before consensus resolution. Across 728 domain-level assessments, observed agreement was 97.66%, with an aggregate Cohen’s κ of 0.959; domain-specific κ values ranged from 0.857 to 1.000. Evidence was synthesized through cross-study analysis and four evidence maps.

**Results:**

Evidence shows use of detection, classification, OCR/imprint recognition, hybrid and multimodal architectures, alongside few-shot, low-shot, zero-shot, and incremental learning. Applications extend to assistive, mobile, and IoT-enabled systems, medication verification, and clinical decision support. Heterogeneity in datasets, imaging conditions, evaluation protocols, and validation strategies limits comparison and generalizability. External validation, cross-dataset testing, unseen-medication evaluation, prospective deployment, and clinical testing remain limited.

**Conclusion:**

AI-based medication recognition is evolving from a computer vision task into a digital informatics component supporting medication safety, accessibility, verification, and patient care. Future research should prioritize diverse datasets, leakage control, independent multicenter validation, unseen-medication evaluation, explainable and privacy-preserving AI, interoperability, and resource-efficient deployment. Evaluation should extend beyond accuracy to clinical utility, safety, usability, trust, and patient-centered outcomes.

## 1 Introduction

### 1.1 Background and motivation

Medication safety remains a pressing global public health priority. Errors can occur at virtually any stage of the medication process—prescribing, transcribing, dispensing, administration, or monitoring—and continue to be a leading cause of preventable patient harm. Beyond the toll on patients, medication-related harm also imposes a considerable economic burden on health systems worldwide. In response, the World Health Organization (WHO) launched the Medication Without Harm initiative, aimed at promoting coordinated global strategies to reduce preventable medication-related harm (1).

Digital health technologies are taking on a growing role in supporting medication-related processes, linking computational methods with clinical workflows, health information systems, and patient-facing services. Artificial intelligence (AI) in particular has become an increasingly valuable tool in healthcare, with the potential to strengthen clinical workflows, improve health-system operations, and enable more data-informed care (2). Within digital health informatics, AI-based medication recognition provides a computational foundation for identifying medicines and linking recognition outputs to medication information, verification processes, and user-facing interfaces.

Reliable medication identification carries particular weight in contexts involving visually similar medicines, older adults, visually impaired individuals, and users who may find medication labeling or instructions difficult to interpret. Progress in computer vision, deep learning, optical character recognition (OCR), and multimodal AI has made it possible to automate several aspects of medication detection, classification, imprint recognition, and information retrieval. These techniques open up meaningful opportunities to support medication identification and verification, yet strong recognition performance on its own does not guarantee clinical effectiveness or measurable gains in medication-safety outcomes (3, 4).

Given these developments, a clear need remains to systematically map the methodological landscape of AI-based medication recognition—examining the datasets in use, evaluation and validation practices, generalizability across populations and settings, deployment challenges, and how such systems might be responsibly integrated into digital-health informatics workflows across diverse healthcare environments.

### 1.2 Medication safety and pharmaceutical identification challenges

Medication errors and adverse drug events (ADEs) remain a persistent problem across healthcare systems, contributing to patient harm, elevated mortality, and rising healthcare costs (5). These errors can arise at nearly any stage of the medication-use process, from prescribing and preparation through dispensing, administration, and monitoring. Evidence from pediatric perioperative care points to the severity such errors can reach in this setting and reinforces the case for digital technologies that can strengthen medication verification and safety practices.

These concerns are especially acute for older adults, who frequently take multiple medications at once and face an elevated risk of inappropriate use and related complications. Nursing-home-based studies have documented preventable medication errors and ADEs, including some arising during the monitoring phase of care. Findings like these speak to the value of intelligent systems built to support medication verification, monitoring, and clinical decision-making, particularly for vulnerable populations (6). Within digital health informatics, AI-based medication recognition can function as an identification layer linking medication images to structured information and broader healthcare workflows.

Counterfeit and substandard medicines add a further layer of difficulty, carrying direct implications for both patient safety and the integrity of pharmaceutical supply chains. Developments in computer vision, artificial intelligence, mobile health, and identification technologies have opened the door to new approaches for pharmaceutical authentication, pill recognition, and medication tracking. Pairing visual recognition with QR codes and digital-health platforms may offer additional support for medication identification and supply-chain transparency (7). Even so, the ability to recognize a medication does not, by itself, confirm pharmaceutical authenticity or guarantee clinical effectiveness.

Taken as a whole, these challenges make the case for dependable AI-based medication-recognition technologies capable of supporting identification and verification within digital-health ecosystems—tools whose real-world value ultimately rests on rigorous validation, interoperability, usability, privacy protection, and careful integration with medication information and human oversight.

### 1.3 Artificial intelligence and digital health technologies for medication recognition

Digital health technologies and artificial intelligence (AI) are being drawn on with increasing frequency to support medication identification, verification, and information-supported healthcare workflows, as shown in Figure 1. Look-alike and sound-alike medications remain a well-recognized source of identification and dispensing errors, particularly in complex healthcare environments. Recent progress in computer vision, deep learning, optical character recognition (OCR), and mobile health has made it possible for AI-based systems to recognize medications and support verification across a range of clinical and consumer settings (8).

**Figure 1:**
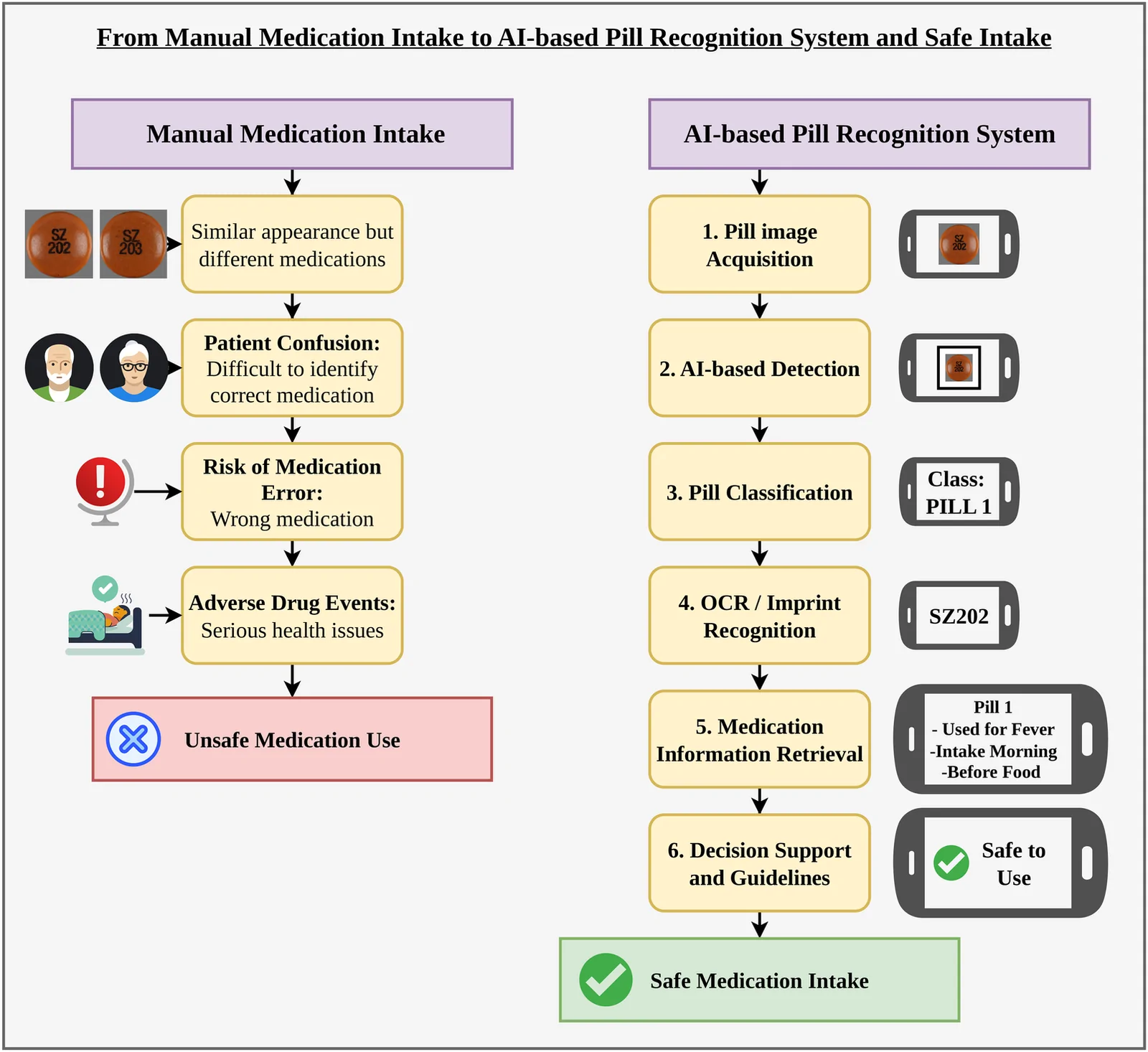
A conceptual overview of the conventional medication intake and AI-based pill recognition system for accurate medication identification and safe usage.

Work in this space has moved away from largely experimental model-building and toward more application-oriented systems, evaluated across cloud platforms, desktop computers, mobile devices, and other deployment environments. The rise of code-free and simplified AI development platforms may further ease technical barriers for non-specialist users looking to implement these tools. Reported performance, even so, continues to hinge heavily on the underlying dataset, imaging conditions, medication formulary, evaluation protocol, and deployment context. System design and evaluation should therefore be guided by the intended clinical workflow, available medication information, usability requirements, and safety considerations, rather than by performance metrics considered in isolation (8).

Bringing AI into digital health informatics also raises questions around transparency, explainability, and how user trust is calibrated. Work examining automated medication verification alongside pharmacists suggests that communicating model uncertainty can help bring clinician trust more closely in line with the actual reliability of AI-generated recommendations. This body of evidence underscores the importance of uncertainty-aware, explainable design wherever recognition outputs are embedded within medication-related digital-health workflows (9).

In general, the literature suggests that AI-based medication recognition operates as a technological interface linking medication images to digital information resources, with potential applications spanning verification, accessibility, and patient-centered care. Recognition performance, however, should not be mistaken for demonstrated clinical effectiveness. Rigorous external validation, usability assessment, workflow integration, and safety evaluation remain essential prerequisites for responsible implementation within digital health informatics (8, 9).

### 1.4 Research challenges and knowledge gaps

While advances have been made in artificial intelligence (AI), computer vision, and digital health, there are still several limitations that hinder the widespread adoption and effectiveness of AI-powered medication recognition systems. Several studies have achieved higher performance in detecting, classifying, recognizing imprints, and verifying medications for pill detection and classification (3, 4, 8), and for medication verification (3), respectively, but most of them use institution-specific datasets, strictly controlled imaging conditions, and a limited number of classes. Reported performance may not necessarily be reproducible across other healthcare settings, patient populations, medication strengths, devices, or image-acquisition settings. Direct comparison and reproducibility between different studies is also hindered by inconsistencies in the composition of datasets, their preprocessing methods, the evaluation protocols, and the benchmarking procedures.

Integrating experimental systems into real healthcare processes is still a major issue. Recognition accuracy is often reported in a controlled setting of studies (3, 4, 8) and comparatively fewer studies are focused on independent or multicenter datasets, prospective real-world deployment, workflow integration, usability, and downstream medication-safety outcomes. These gaps are most important for vulnerable populations, and for medication-use processes where medication errors and adverse drug events continue to be an issue (5, 6).

In addition to the technical aspects, explainability, transparency, privacy and security, interoperability, scalability, and the establishment of appropriate user trust are critical for the successful implementation. Uncertainty-aware and trustworthy AI implies that recognition results should be explainable and comprehensible to both medical staff and patients (9). More importantly, higher recognition performance should not necessarily be viewed as evidence of clinical effectiveness, unless validated.

Much of this literature is scattered across computer vision, deep learning, mobile health, and healthcare informatics literature (3, 4, 8, 9). There is therefore a need for a systematic synthesis to collate this evidence, identify the lack of generalizability and deployment, and clarify the significance of this evidence in relation to digital health informatics, which could eventually help to develop safe and clinically relevant medication-recognition systems.

### 1.5 Research questions and contributions

Artificial intelligence (AI), computer vision, and digital health technologies have together pushed the field of medication recognition forward, drawing on deep learning, optical character recognition, mobile health, and health informatics. Yet the relevant evidence base remains scattered across methodological approaches, datasets, evaluation strategies, deployment environments, and healthcare applications, making it difficult to form a consolidated picture of where the field currently stands. To help close these gaps, this review was structured around the following research questions:

RQ1. Which recent AI methodologies and computational frameworks have been applied to medication recognition and identification?

RQ2. How do existing medication recognition approaches differ in terms of methodological category, architecture, dataset composition, reported performance, and application setting?

RQ3. Which datasets, evaluation metrics, validation strategies, generalizability assessments, and deployment characteristics are reported across the medication recognition literature?

RQ4. What technical, clinical, operational, and implementation challenges affect the real-world deployment of AI-based medication recognition technologies?

RQ5. In what ways might current medication recognition systems support medication safety, clinical decision support, healthcare access, and patient-centered digital health services?

RQ6. What emerging research trends, methodological gaps, and future directions could help build safer, more generalizable, scalable, and clinically relevant medication recognition systems?

In addressing these questions, this systematic review brings together the available evidence on AI-based medication recognition within a digital health informatics context, covering computational methods, datasets, evaluation practices, validation approaches, generalizability, deployment, and healthcare applications. The review also presents a structured cross-study synthesis, organizing included studies into detection, classification, OCR/imprint recognition, detection–classification hybrid, multimodal and intelligent hybrid, and few-shot/zero-shot methodological categories. To strengthen transparency, the review drew on independent dual screening, full-text assessment, evidence mapping, and a manual D1–D8 methodological assessment, enabling a systematic characterization of methodological limitations, validation strength, generalizability, and deployment considerations across the included evidence.

### 1.6 Organization of the review

In Section 2, the methodological framework used to conduct this systematic review is outlined, including the PRISMA 2020 framework, search strategy, inclusion and exclusion criteria, study selection process, quality assessment by two independent reviewers, and data extraction. Section 3 concludes by exploring the theoretical underpinnings of medication recognition from a digital health informatics lens, with a focus on computer vision, medication safety, health care workflows, and patient-focused applications. In Section 4, a structured taxonomy is given for the medication recognition approaches that were identified in the literature.

In Section 5, the various architectures and computational methods used in medication recognition research are summarized, including deep learning, computer vision, OCR, multimodal, few-shot, low-shot, and zero-shot. Section 6 presents a structured cross-study synthesis of the 91 studies included, which is based on datasets, evaluation methods, performances reported, the type of validation undertaken, generalizability, deployment characteristics, and methodological limitations. This section also consolidates comparative tables, evidence maps, and the manual D1–D8 methodological assessment to facilitate a contextual interpretation of the evidence, rather than a direct comparison of models under various conditions. Section 7 then discusses the key research gaps and challenges that influence the generalizability, validation, deployment, usability, trust, privacy, and scalability of AI systems.

In Section 8, the reported and potential uses of medication recognition systems in digital health and health care workflow are examined from the perspectives of medication safety, pharmacovigilance, clinical decision support, mobile and assistive applications, and systems incorporating IoT. In Section 9 we discuss future research trends, such as multimodal AI, foundation models, explainable AI, federated learning, edge intelligence, and generative AI. The discussion is presented in Section 10 and the review is concluded by summarizing the main findings, contributions, limitations, and priorities for future research in Section 11.

## 2 Systematic review methodology

### 2.1 Review framework and PRISMA protocol

This study employed a systematic review methodology to synthesize the available evidence on artificial intelligence (AI)-based medication recognition, with particular emphasis on computational approaches for identifying, detecting, classifying, and recognizing medications from visual, textual, or multimodal information. The review was designed to characterize the methodological landscape of medication-recognition systems and to examine their evaluation, validation, generalizability, and potential application within digital-health contexts. The review was conducted in accordance with the Preferred Reporting Items for Systematic Reviews and Meta-Analyses (PRISMA) 2020 guidelines (10).

The review procedures were predefined and applied consistently throughout the study-selection and evidence-synthesis process. The review was not prospectively registered in PROSPERO or another public review registry, and no formal review protocol was prepared. The review comprised five sequential stages: identification of potentially relevant records, removal of duplicate records, title-and-abstract screening, full-text eligibility assessment, and final inclusion of eligible studies. These stages were maintained as distinct steps to ensure a transparent separation between record retrieval, preliminary screening, and final study eligibility.

The review covered publications available from 1 January 2016 through 4 September 2026, with the final literature search conducted on 4 September 2026. The search was designed to capture technically focused studies of AI-based medication recognition without requiring additional application-specific concepts that could unnecessarily restrict retrieval. Accordingly, medication-recognition concepts and AI-method concepts formed the principal search framework, while characteristics such as medication safety, digital-health application, and deployment use were considered during subsequent study assessment and synthesis where applicable.

Study selection involved independent assessment by two reviewers, with disagreements resolved through discussion and consensus. Both reviewers independently assessed the full texts of records retained after title-and-abstract screening and independently performed the study-level methodological assessment, followed by discussion and consensus.

The complete study-selection pathway, including identification, deduplication, screening, full-text assessment, and final inclusion, is presented in the PRISMA 2020 flow diagram in Figure 2. As shown in Figure 3, detailed procedures for searching, eligibility assessment, reviewer agreement, methodological assessment, and evidence synthesis are described in the following subsections.

**Figure 2:**
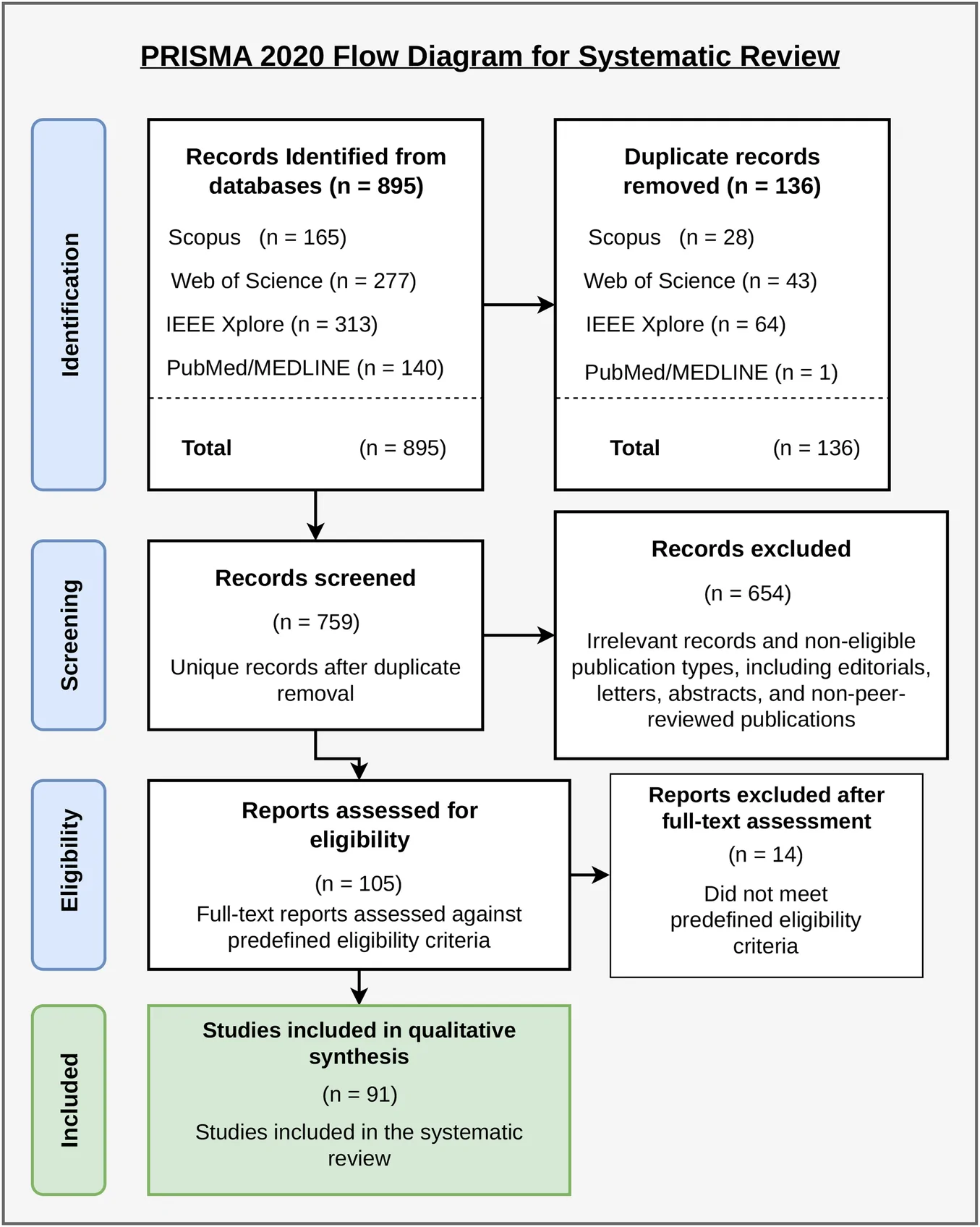
PRISMA 2020 flow diagram of the study selection process.

**Figure 3:**
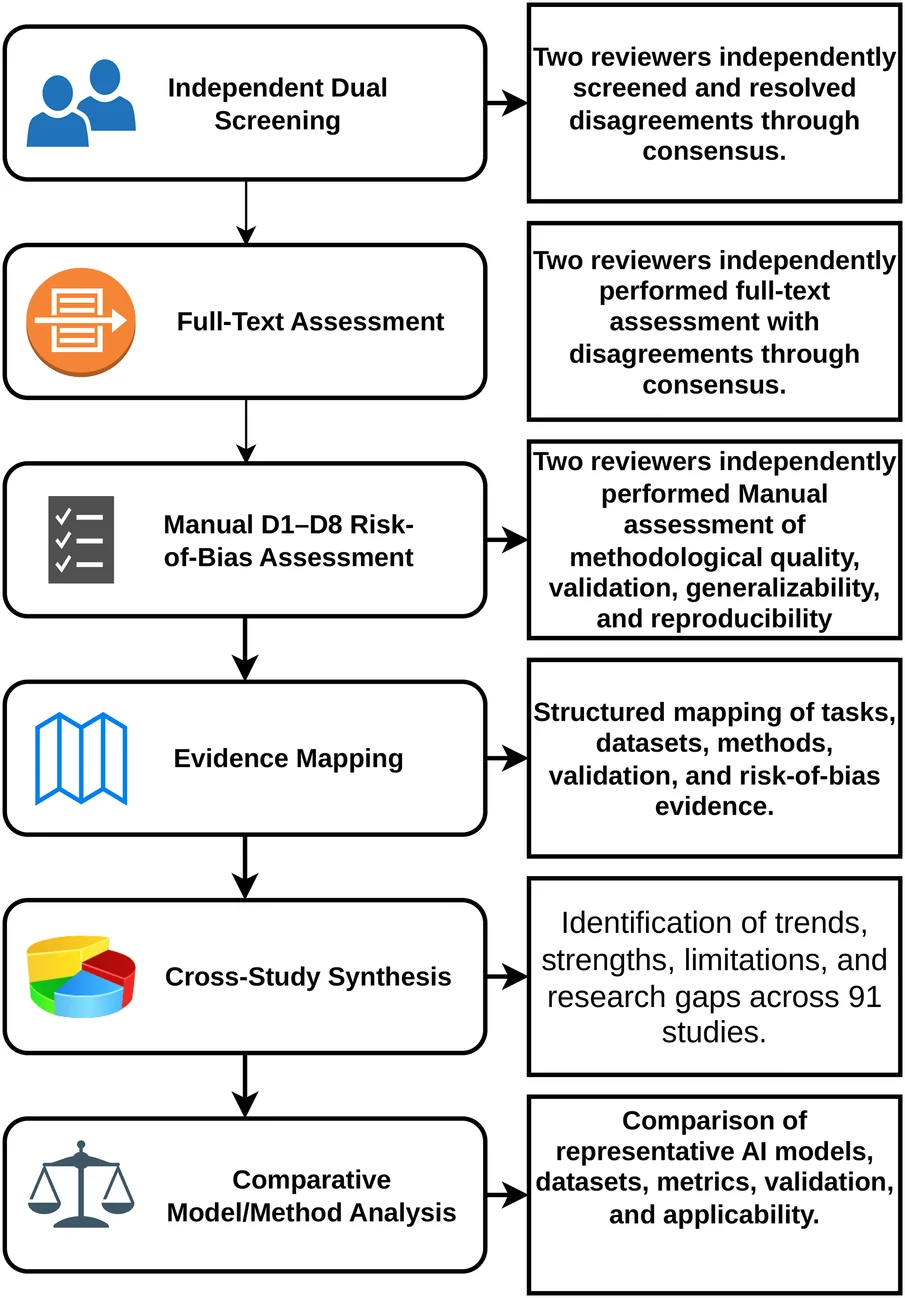
Study assessment and synthesis framework.

### 2.2 Research questions

Research questions were defined to provide a structured framework for the systematic review, data extraction, evidence mapping, cross-study synthesis, and interpretation of the included studies. The questions guided the examination of artificial intelligence (AI)-based medication recognition, including the recognition tasks, methodological approaches, datasets, evaluation practices, validation strategies, generalizability, and application characteristics reported in the literature.

The research questions were designed to investigate the methodological and technical landscape of AI-based medication recognition while also considering its reported real-world and healthcare application contexts. They guided the assessment of medication detection, classification, identification, OCR and imprint recognition, as well as hybrid, multimodal, few-shot, low-shot, and zero-shot approaches. The questions also supported examination of dataset characteristics, performance evaluation, validation and external generalizability, reproducibility, deployment evidence, and methodological concerns identified through the D1–D8 assessment framework.

The research questions further provided the basis for the structured evidence maps and cross-study synthesis, enabling the identification of methodological trends, strengths, limitations, generalizability constraints, deployment gaps, and research opportunities across the 91 included studies. Application-related characteristics, including medication safety, digital-health, mobile, and IoT contexts, were considered where reported but were not treated as independent requirements for study inclusion.

### 2.3 Data sources and search strategy

A comprehensive literature search was conducted in Scopus, Web of Science, IEEE Xplore, and PubMed/MEDLINE to identify studies addressing AI-based medication and pill recognition. The search covered publications from 1 January 2016 through 4 September 2026 and was conducted on 4 September 2026. English-language publications were considered. These databases were selected to provide complementary coverage of computational, biomedical, healthcare, and digital-health research.

To maximize retrieval of technically focused medication-recognition studies, the search strategy comprised two conceptual blocks: (1) medication/pill recognition and (2) AI and computational methods. Terms within each block were combined using OR, and the two blocks were combined using AND. The medication-recognition block included terms related to pill, tablet, capsule, medication, medicine, and drug recognition, detection, classification, identification, image recognition, imprint recognition, and OCR. The AI-method block included artificial intelligence, deep learning, computer vision, convolutional neural networks, few-shot learning, zero-shot learning, and multimodal learning.

Database-specific field operators were used according to each platform: TITLE-ABS-KEY in Scopus, TS in Web of Science, database-supported search fields in IEEE Xplore, and title/abstract ([tiab]) fields in PubMed. The search keywords and conceptual search blocks are summarized in Table 1, while the database-specific search fields and search characteristics are presented in Table 2.

**Table 1:** Search keywords and conceptual blocks.

Database	Search Keywords
Scopus, Web of Science, IEEE Xplore, and PubMed	**Block 1 — Medication/Pill Recognition:** (“pill recognition” OR “pill detection” OR “pill classification” OR “pill identification” OR “pill image recognition” OR “pill image classification” OR “tablet recognition” OR “tablet detection” OR “tablet classification” OR “tablet identification” OR “tablet image recognition” OR “capsule recognition” OR “capsule detection” OR “capsule classification” OR “capsule identification” OR “medication recognition” OR “medication detection” OR “medication classification” OR “medication identification” OR “medication image recognition” OR “medicine recognition” OR “medicine detection” OR “medicine classification” OR “medicine identification” OR “medicine image recognition” OR “drug recognition” OR “drug detection” OR “drug classification” OR “drug identification” OR “drug image recognition” OR “drug image classification” OR “imprint recognition” OR “pill imprint recognition” OR “medication imprint recognition” OR “pill imprint” OR “pill OCR” OR “medication OCR”) AND **Block 2 — AI Methods:** (“artificial intelligence” OR “deep learning” OR “computer vision” OR “convolutional neural network” OR “few-shot learning” OR “zero-shot learning” OR “multimodal learning” OR “multimodal”)

**Table 2:** Database sources and search characteristics.

Database	Search field	Search date	Date range	Language	Records
Scopus	TITLE-ABS-KEY	4 September 2026	2016–2026	English	165
Web of Science	TS	4 September 2026	2016–2026	English	277
IEEE Xplore	Database-supported search fields	4 September 2026	2016–2026	English	313
PubMed/MEDLINE	Title/Abstract ([tiab])	4 September 2026	2016–2026	English	140
**Total**					**895**

Bold values indicate total or overall results, where applicable.

The search retrieved 895 records: 313 from IEEE Xplore, 277 from Web of Science, 165 from Scopus, and 140 from PubMed. Retrieved records were exported and consolidated for duplicate removal and subsequent independent title-and-abstract screening. The detailed study-selection process and resulting full-text eligibility assessment are described in the following subsection.

### 2.4 Inclusion and exclusion criteria

Predefined inclusion and exclusion criteria were established before study selection and were applied consistently during title and abstract screening and subsequent full-text eligibility assessment. The criteria were designed to distinguish primary studies addressing medication recognition from adjacent medication-management, pharmaceutical, healthcare, and digital-health applications. The detailed criteria are presented in Table 3.

**Table 3:** Inclusion and exclusion criteria applied during the study selection process.

Inclusion Criteria	Exclusion Criteria
Primary studies addressing direct medication, pill, tablet, or capsule recognition, detection, classification, identification, imprint recognition, or medication-related OCR from visual, textual, or multimodal information.	Studies in which medication recognition was not the primary research objective, including general medication-management, pharmaceutical informatics, healthcare, or digital-health studies without a direct medication-recognition component.
Studies applying artificial intelligence or related computational methods, including machine learning, deep learning, computer vision, convolutional neural networks, few-shot, low-shot, zero-shot, or multimodal learning, to the recognition task.	Studies focused on drug development, drug delivery, chemistry, pharmacology, manufacturing, pill counting, missing-pill detection, tablet-defect detection, or other pharmaceutical applications without direct medication recognition.
Peer-reviewed journal articles and peer-reviewed conference papers reporting original research.	Secondary reviews, systematic reviews, surveys, editorials, letters, patents, dissertations, theses, abstracts, presentations, and other non-peer-reviewed publications. Secondary reviews were not included as primary evidence but could be used for contextual or background information.
Studies reporting sufficient information on the dataset or data source, recognition methodology, evaluation procedure, experimental results, or relevant application characteristics.	Studies lacking sufficient methodological or evaluation information to determine the nature or performance of the recognition approach.
English-language publications with accessible full text.	Non-English publications or publications for which the full text could not be accessed.
Studies reporting medication safety, medication management, digital-health or related healthcare applications in association with a direct medication-recognition task, where such characteristics were reported.	Medical imaging or diagnostic studies unrelated to direct medication recognition, as well as general recognition or classification studies without a medication-recognition application.

Studies were eligible for inclusion when the primary research objective involved the direct recognition, detection, classification, identification, or imprint/OCR recognition of medications, pills, tablets, or capsules. Eligible studies could use visual, textual, or multimodal information and were required to apply artificial intelligence or related computational methods to the medication-recognition task. The included methodological approaches encompassed deep learning, computer vision, convolutional neural networks, hybrid frameworks, few-shot learning, low-shot learning, zero-shot learning, and multimodal learning.

The eligibility criteria were intentionally focused on primary medication-recognition research rather than on medication management or pharmaceutical informatics more broadly. Studies addressing medication safety, medication management, digital health, Internet of Things (IoT), or related healthcare applications were considered eligible only when a direct medication-recognition component was present and formed a substantive part of the study. These characteristics were therefore treated as application or contextual dimensions during data extraction and synthesis rather than as independent requirements for retrieval or inclusion.

Studies were excluded when medication recognition was not the primary research objective or when the reported system addressed an adjacent application without directly recognizing medications. This included studies concerned primarily with drug development, drug delivery, chemistry, pharmacology, manufacturing, pill counting, missing-pill detection, tablet-defect detection, or other pharmaceutical processes without a direct medication-recognition task. Medical imaging and diagnostic studies unrelated to direct medication recognition were also excluded. Studies using only conventional image processing or OCR without a qualifying artificial-intelligence or computational learning component were excluded when they did not satisfy the predefined AI-based recognition criterion.

Secondary reviews, systematic reviews, and surveys were not included as primary evidence in the final synthesis. Such publications could be consulted for background or contextual understanding, but the evidence synthesis was restricted to eligible primary research studies. Editorials, letters, patents, dissertations, theses, abstracts, presentations, and other non-peer-reviewed publications were excluded. Studies were also excluded when insufficient methodological or evaluation information was available to determine the nature of the recognition approach or its reported results, or when the publication was not available in English, or the full text could not be accessed.

The same eligibility criteria were applied during both title and abstract screening and full-text assessment. Following title and abstract screening, 105 records were retained for full-text eligibility assessment. Of these, 14 records were excluded after full-text assessment because they did not satisfy the predefined eligibility criteria. The remaining 91 studies satisfied all inclusion criteria and were included in the final qualitative synthesis, evidence mapping, methodological assessment, and cross-study synthesis.

### 2.5 Dual study-selection process

The study-selection process followed the PRISMA 2020 framework and comprised identification, deduplication, title and abstract screening, full-text eligibility assessment, and final inclusion. Following removal of duplicate records, the remaining records were independently assessed against the predefined inclusion and exclusion criteria.

The study-selection process was conducted by two reviewers. Reviewer 1 compiled the retrieved database records and screening dataset, while Reviewer 1 and Reviewer 2 independently screened the titles and abstracts. The same two reviewers independently assessed the full texts of records retained after title and abstract screening. Their decisions were compared, and any disagreements during title and abstract screening or full-text assessment were discussed and resolved through consensus.

A total of 759 unique records underwent independent title and abstract screening, as shown in Figure 2. The reviewers agreed on 643 records and disagreed on 116 records, corresponding to an observed agreement of 84.72%. Cohen’s kappa (11) was used to quantify inter-rater agreement beyond chance and was calculated as in (Equation 1):

$$\kappa =\frac{P_{o}-P_{e}}{1-P_{e}},$$ (1)

where $P_{o}$ represents the observed proportion of agreement and $P_{e}$ represents the expected proportion of agreement by chance. For the 759 screened records, the resulting Cohen’s κ was 0.527, indicating moderate agreement. Following consensus resolution, 105 records were retained for full-text eligibility assessment, while 654 were excluded during title and abstract screening.

The 105 retained records underwent full-text assessment by both reviewers. Finally, 91 studies were included in the final qualitative synthesis, methodological assessment, evidence mapping, and cross-study synthesis.

Full-text exclusion audit. To improve transparency and reproducibility of the eligibility assessment, the reasons for excluding the 14 reports that did not satisfy the predefined eligibility criteria at the full-text stage were systematically recorded. The exclusions primarily reflected differences in the primary study objective, recognition scope, methodological contribution, and research design. A concise categorization of the full-text exclusion reasons is provided in Table 4, while the paper-level exclusion decisions were retained in the screening audit dataset.

**Table 4:** Summary of reasons for exclusion after full-text assessment (n=14).

Full-text exclusion category	Studies (n)
Medication recognition task not directly addressed	2
Image processing without a qualifying recognition contribution	1
AI-based medication-recognition methodology not sufficiently established	7
Text-recognition/imprint-focused rather than broader medication recognition	3
Case study/tutorial-style rather than clearly a primary research contribution	1
**Total**	**14**

Bold values indicate total or overall results, where applicable.

The complete study-selection pathway is presented in the PRISMA 2020 flow diagram in Figure 2.

### 2.6 Manual risk-of-bias assessment

Because no single validated risk-of-bias instrument was identified that adequately captured the methodological characteristics specific to AI-based medication-recognition studies, a structured manual D1–D8 risk-of-bias assessment framework was developed. The framework was designed to assess methodological concerns across diverse AI methods, datasets, evaluation procedures, and application contexts. Existing appraisal frameworks were considered; however, they were not adopted because they did not jointly address medication-image dataset quality, annotation and data leakage, task-specific evaluation, external validation, reproducibility, real-world deployment, and AI-specific transparency and safety within a single framework.

The framework comprised eight predefined domains: D1—Dataset Quality; D2—Ground Truth/Annotation; D3—Data Split/Leakage; D4—Evaluation Methodology; D5—Generalizability/External Validation; D6—Reproducibility/Reporting; D7—Real-world/Clinical Applicability; and D8—Transparency/Safety. Each domain was assessed using the predefined signaling questions, operational considerations, and judgment criteria presented in Table 5.

**Table 5:** D1–D8 manual risk-of-bias assessment rubric and operational criteria.

Domain	Signaling question	Operational considerations	Judgment
D1 Dataset Quality	Is the dataset sufficiently described, appropriate, and diverse for the recognition task?	Dataset size, diversity, representativeness, image/data quality, and suitability for the stated task.	Low/Moderate / High
D2 Ground Truth and Annotation	Are ground-truth labels or annotations adequately defined and reliable?	Annotation procedure, label definition, annotation quality, verification, and completeness.	Low/Moderate / High
D3 Data Splitting and Leakage	Are training, validation, and test data appropriately separated and potential leakage addressed?	Split methodology, independence of test data, patient/subject or image overlap where applicable, and leakage controls.	Low/Moderate / High
D4 Evaluation Methodology	Is the evaluation methodology appropriate and sufficiently reported for the recognition task?	Evaluation design, performance measures, test procedure, experimental protocol, and appropriateness of reported metrics.	Low/Moderate / High
D5 Generalizability and External Validation	Is there evidence that the approach generalizes beyond the development dataset or controlled setting?	External validation, cross-dataset testing, unseen-medication evaluation, independent data, and diversity of evaluation conditions.	Low/Moderate / High
D6 Reproducibility and Reporting	Is sufficient methodological information reported to understand and reproduce the approach?	Dataset description, model/configuration details, preprocessing, training procedure, experimental settings, and implementation information.	Low/Moderate/High
D7 Real-world/Clinical Applicability	Is there evidence that the approach can operate in realistic or healthcare-related settings?	Deployment evidence, workflow integration, usability-related evidence, clinical/real-world testing, and practical applicability.	Low/Moderate / High
D8 Transparency/Safety	Are limitations, uncertainty, transparency, explainability, and safety considerations adequately addressed?	Reporting of limitations, uncertainty, explainability, safety considerations, risks, and responsible-use information.	Low/Moderate / High

Each domain received a standardized judgment of Low, Moderate, or High methodological concern. Low concern indicated that the relevant methodological feature was adequately described and appropriately addressed; Moderate concern indicated incomplete reporting or identifiable limitations; and High concern indicated substantial methodological limitations, inadequate reporting, or a clear risk of bias. An overall study-level assessment was derived qualitatively from the pattern and materiality of domain-specific concerns, and no numerical composite risk-of-bias score was assigned.

Both reviewers independently applied the D1–D8 framework to the 91 included studies before consensus resolution. Their independent ratings were compared for inter-rater agreement using observed percentage agreement and Cohen’s kappa. Across 728 paired domain-level assessments (91 studies × 8 domains), the reviewers agreed on 711 assessments, corresponding to 97.66% observed agreement, with an aggregate Cohen’s κ of 0.959, as summarized in Table 6. Domain-specific observed agreement ranged from 94.51% to 100.00%, while Cohen’s κ values ranged from 0.857 to 1.000. The 17 domain-level disagreements were subsequently discussed and resolved through consensus.

**Table 6:** Inter-rater agreement for the D1–D8 methodological assessment across the 91 included studies.

Domain	Observed agreement (%)	Cohen’s κ
D1 Dataset Quality	100.00	1.000
D2 Ground Truth/Annotation	95.60	0.913
D3 Data Split/Leakage	98.90	0.982
D4 Evaluation Methodology	98.90	0.977
D5 Generalizability/External Validation	96.70	0.940
D6 Reproducibility/Reporting	98.90	0.979
D7 Real-world/Clinical Applicability	97.80	0.959
D8 Transparency/Safety	94.51	0.857
**Overall D1–D8**	**97.66**	**0.959**

Bold values indicate total or overall results, where applicable.

Cohen’s kappa was calculated using the three predefined judgment categories: Low, Moderate, and High. Agreement was calculated exclusively from the independent pre-consensus ratings of Reviewer 1 and Reviewer 2; the finalized consensus ratings were not used in the inter-rater agreement calculation. The finalized D1–D8 assessments were subsequently used for the study-level methodological synthesis and D5 generalizability assessment.

The D1–D8 framework was used as a study-level assessment of methodological concerns and was not intended to provide an overall certainty-of-evidence rating across the body of evidence. The high inter-rater agreement supports consistent application of the predefined assessment criteria between the two reviewers; however, the framework remains a manually developed assessment approach and should not be interpreted as a validated certainty-of-evidence instrument. No formal publication bias assessment, sensitivity analysis, or quantitative certainty-of-evidence assessment was performed due to substantial heterogeneity in study designs, recognition tasks, datasets, evaluation protocols, and reported outcomes. These limitations were considered when interpreting the synthesized evidence.

### 2.7 Data extraction

A structured data-extraction process was used to ensure consistent capture and organization of study-level evidence. Reviewer 1 compiled the retrieved records and extracted the study-level information, while Reviewer 2 independently verified the extracted information. Any discrepancies identified during verification were discussed and resolved through consensus. The extraction process captured publication details, study objectives, AI-based recognition task, methodological approach, dataset or data source, dataset size, model or architecture, performance metrics, validation strategy, generalizability, deployment characteristics, and reported healthcare or digital-health application context.

Additional methodological variables were extracted where reported, including data-splitting procedures, information relevant to potential data leakage, OCR or imprint recognition, and safety-related characteristics. Study-specific dataset names, methodological details, and reported performance measures were retained to preserve the context of individual studies.

For cross-study synthesis, AI-based recognition approaches were categorized into the finalized methodological families: Detection, Classification, OCR/Imprint Recognition, Hybrid, Multimodal, Few-Shot, Low-Shot, and Zero-Shot. Each study was assigned one primary methodological family for the taxonomy, while complementary methods and application characteristics were retained in the study-level dataset. Dataset characteristics and validation strategies were similarly standardized for synthesis while preserving the original study-specific information.

Performance measures were extracted as reported and were not converted into a single common metric because detection, classification, OCR/imprint recognition, retrieval, and other recognition tasks use different evaluation criteria. Comparisons were therefore interpreted within appropriate task, dataset, and evaluation contexts rather than as a universal ranking of AI models.

The finalized extraction dataset for the 91 included studies was used to construct the evidence maps and cross-study synthesis, examining methodological approaches, dataset characteristics, evaluation and validation practices, generalizability, reproducibility, deployment characteristics, and research gaps.

### 2.8 Evidence mapping

A structured evidence-mapping framework was used to provide a transparent overview of the methodological characteristics of the 91 included studies. The maps were generated from the finalized study-level extraction and D1–D8 assessment datasets and were designed to describe relationships among AI tasks, methodological families, datasets, performance-metric reporting, generalizability, and methodological concerns.

Four complementary evidence maps were constructed. Map 1A examined the distribution of AI tasks and methodological families, Map 1B examined dataset categories across AI tasks, Map 2A examined AI tasks and performance-metric reporting status, and Map 2D examined AI tasks and D5 generalizability/external-validation assessments. The maps were descriptive and were not intended to rank AI models or establish comparative performance across heterogeneous datasets, recognition tasks, and evaluation protocols.

The evidence maps were generated programmatically using Python code executed in Google Colab from the finalized study-level CSV records. The same standardized study-level records used for the evidence synthesis and relevant methodological assessments were used to generate the corresponding map categories and visualizations. Each map was checked against the underlying study records to ensure consistency of study identifiers, methodological categories, dataset categories, performance-metric reporting status, and D5 generalizability/external-validation classifications. The resulting maps were subsequently used to support the cross-study synthesis.

#### 2.8.1 AI task and methodological family distribution

Evidence Map 1A (Figure 4) visualizes the distribution of the primary methodological families across the standardized AI-task categories and provides an overview of the methodological landscape of medication recognition.

**Figure 4:**
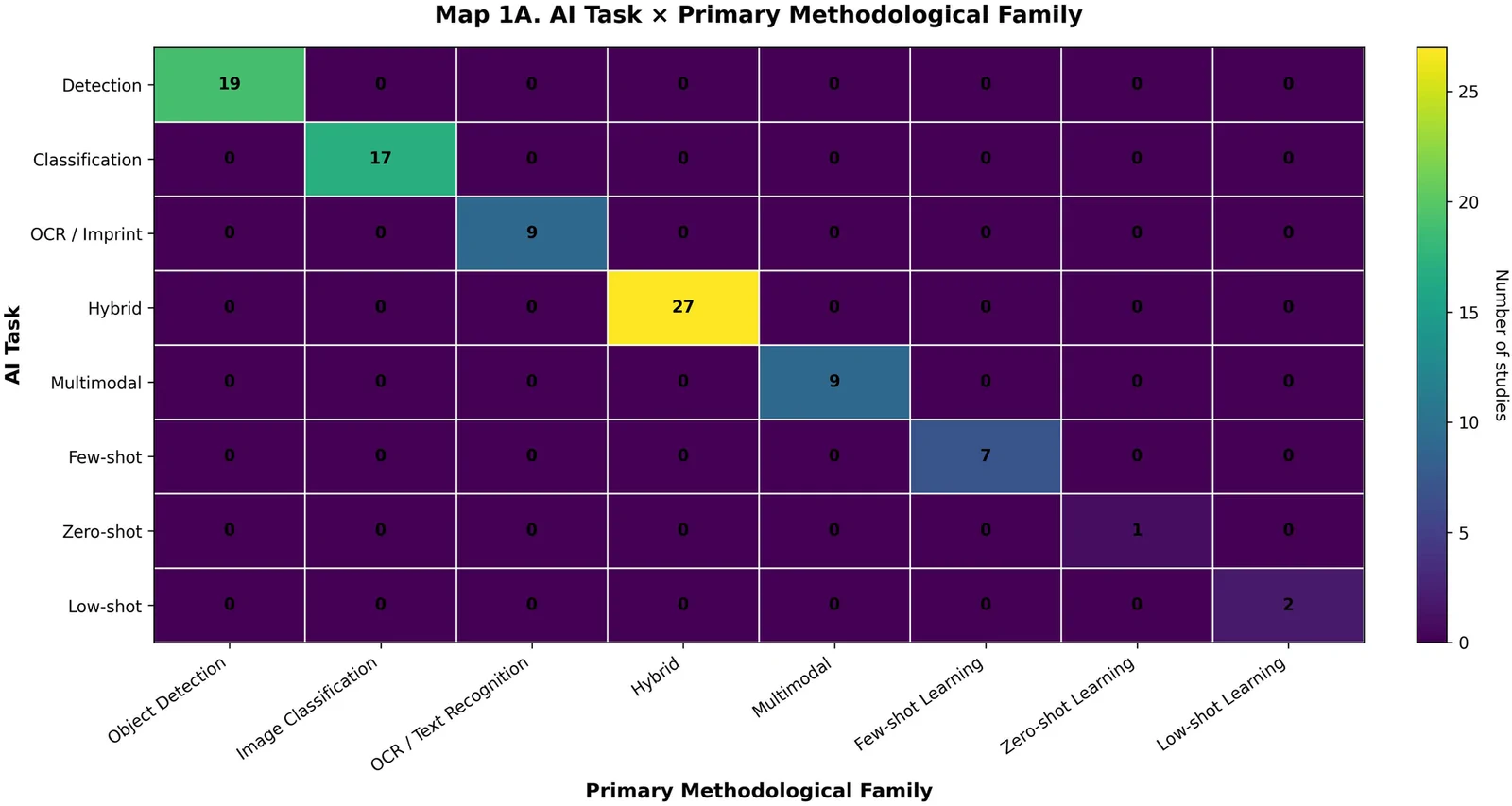
Evidence Map 1A: AI task and methodological family distribution.

#### 2.8.2 Dataset and AI task distribution

Evidence Map 1B (Figure 5) shows the distribution of standardized dataset categories across the AI-task categories while retaining study-level dataset information.

**Figure 5:**
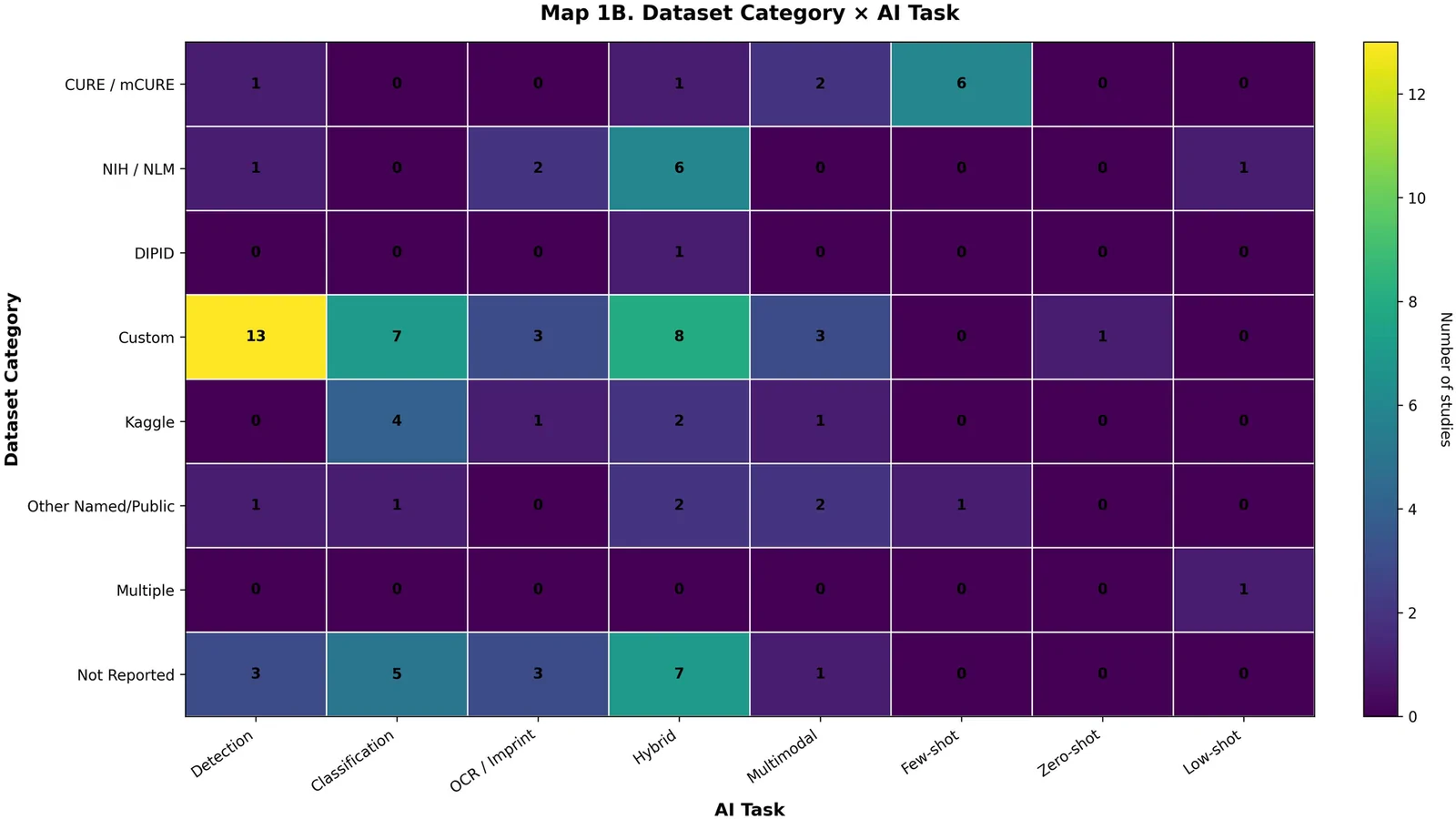
Evidence Map 1B: Dataset and AI task distribution.

#### 2.8.3 AI task and performance metrics reporting

Evidence Map 2A (Figure 6) presents the status of performance-metric reporting across the AI-task categories, distinguishing studies that reported performance metrics, partially reported them, or did not report them.

**Figure 6:**
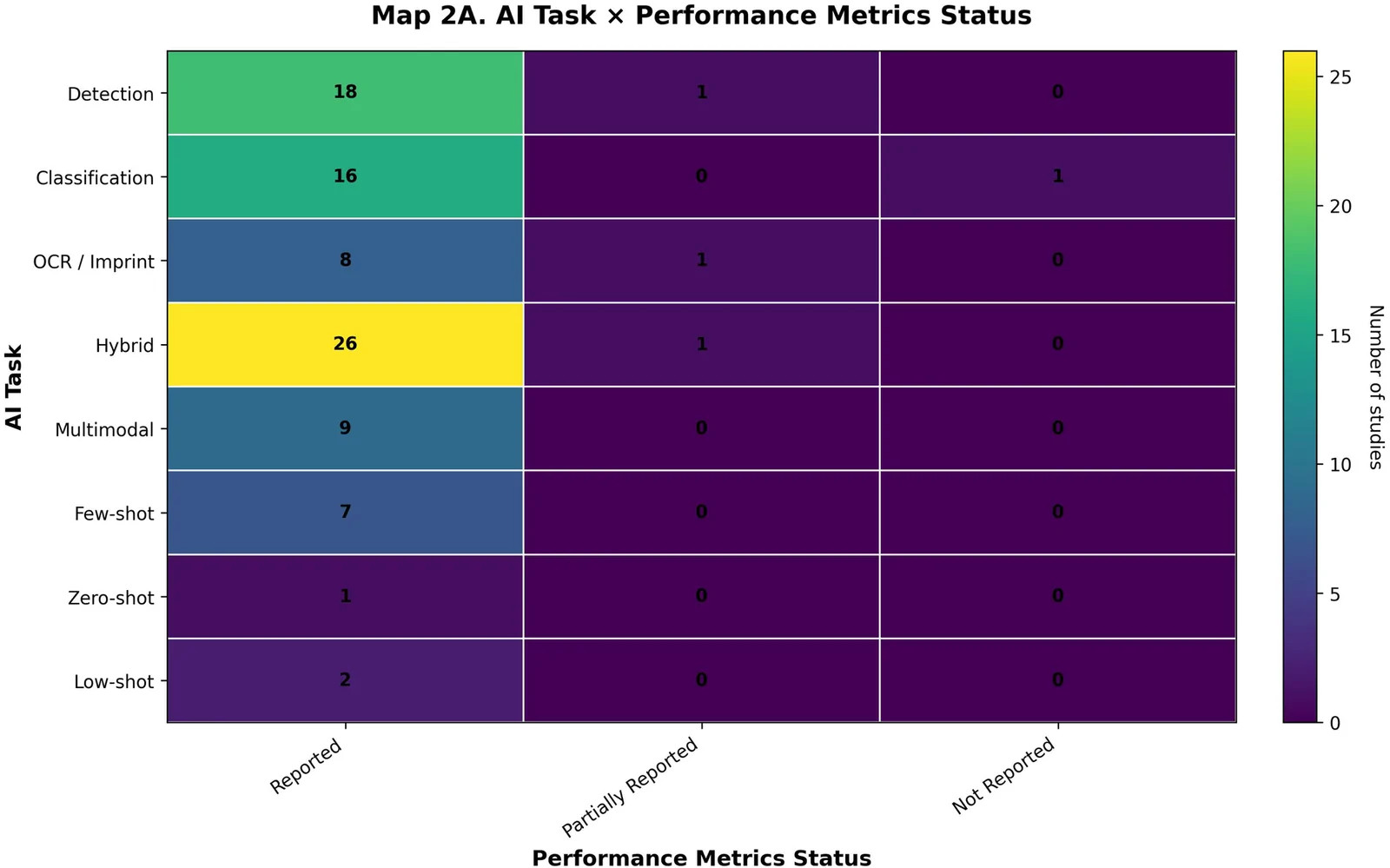
Evidence Map 2A: AI task and performance-metric reporting status.

#### 2.8.4 AI task and generalizability/external validation assessment

Evidence Map 2D (Figure 7) presents the AI-task categories according to the D5 Generalizability/External Validation assessment, highlighting differences in the reported evidence supporting generalizability beyond the development or evaluation setting.

**Figure 7:**
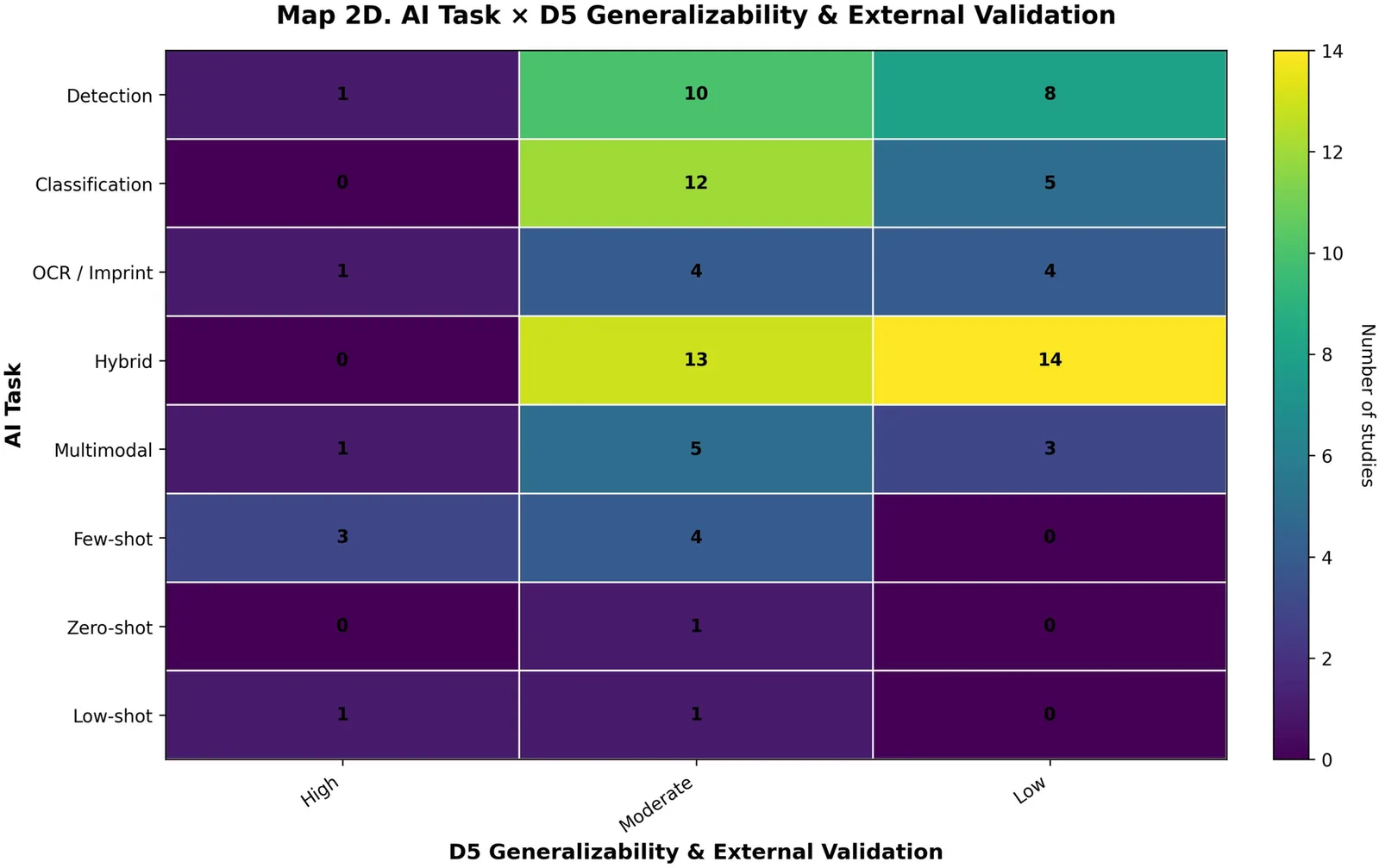
Evidence Map 2D: AI task and generalizability/external-validation assessment.

### 2.9 Cross-study synthesis

A structured cross-study synthesis was conducted using the finalized study-level dataset of 91 included studies to integrate evidence across AI tasks, methodological families, datasets, evaluation strategies, validation, generalizability, deployment, and methodological assessment. Given substantial heterogeneity in recognition tasks, datasets, model architectures, performance measures, and experimental protocols, the synthesis was qualitative and descriptive rather than based on pooled quantitative estimates.

The 91 included studies constituted the complete analytical evidence base for the review. For cross-study interpretation, 53 studies provided sufficiently comparable methodological, dataset, evaluation, and performance information to support structured within-task comparison, while 36 studies were classified as conditionally comparable because limitations in methodological, dataset, or evaluation reporting restricted direct numerical interpretation. Two additional studies were retained for narrative synthesis because the available evidence did not support structured cross-study comparison. Thus, all 91 studies contributed to the overall evidence synthesis, while the depth of comparative interpretation varied according to the completeness and comparability of the reported evidence.

Studies were first characterized according to their primary AI-based medication-recognition task and methodological family. Dataset characteristics, validation strategies, D1–D8 methodological assessments, and deployment-related characteristics were then examined across these categories. Reported performance metrics were retained in their original form and interpreted within the corresponding task, dataset, and evaluation context. Direct comparisons were therefore restricted to studies with sufficiently comparable tasks, datasets, evaluation protocols, and reported outcomes. Models were not ranked universally across heterogeneous studies, and higher reported performance was not interpreted as evidence of overall superiority when experimental conditions differed.

For concise presentation in the main manuscript, 34 representative studies were selected from the complete evidence base to illustrate methodological, dataset, evaluation, and model-comparison diversity rather than to rank models according to reported performance. Thirty-three representative studies were selected from the structured-comparison group, and one additional detection study was selected from the conditionally comparable group because it provided an explicit within-study comparison of multiple detection architectures. The complete structured-comparison and conditionally comparable study sets were retained in the Supplementary Material, while the 34 representative studies were used to construct the main-text comparative tables.

Selection of representative studies was based on methodological and evidentiary coverage rather than reported performance. The selection considered representation across the major recognition tasks and methodological families, variation in dataset type and scale, availability of quantitative performance results, differences in evaluation and validation settings, and diversity in application or deployment context. Studies with highly similar methodological configurations and evaluation settings were not repeatedly represented when their inclusion would provide limited additional comparative information. Accordingly, the 34-study subset was intended to provide broad coverage of the comparative evidence while maintaining a concise main-text presentation.

The cross-study synthesis integrated findings on methodological approaches, dataset characteristics, validation practices, generalizability, reproducibility, deployment evidence, and reported performance to identify consistent patterns and evidence gaps. As shown in Figure 8, a dedicated cross-study synthesis visualization was generated from the finalized study-level dataset to visualize the distribution of studies across the synthesis categories and provide traceability between the individual-study evidence and the overall synthesis.

**Figure 8:**
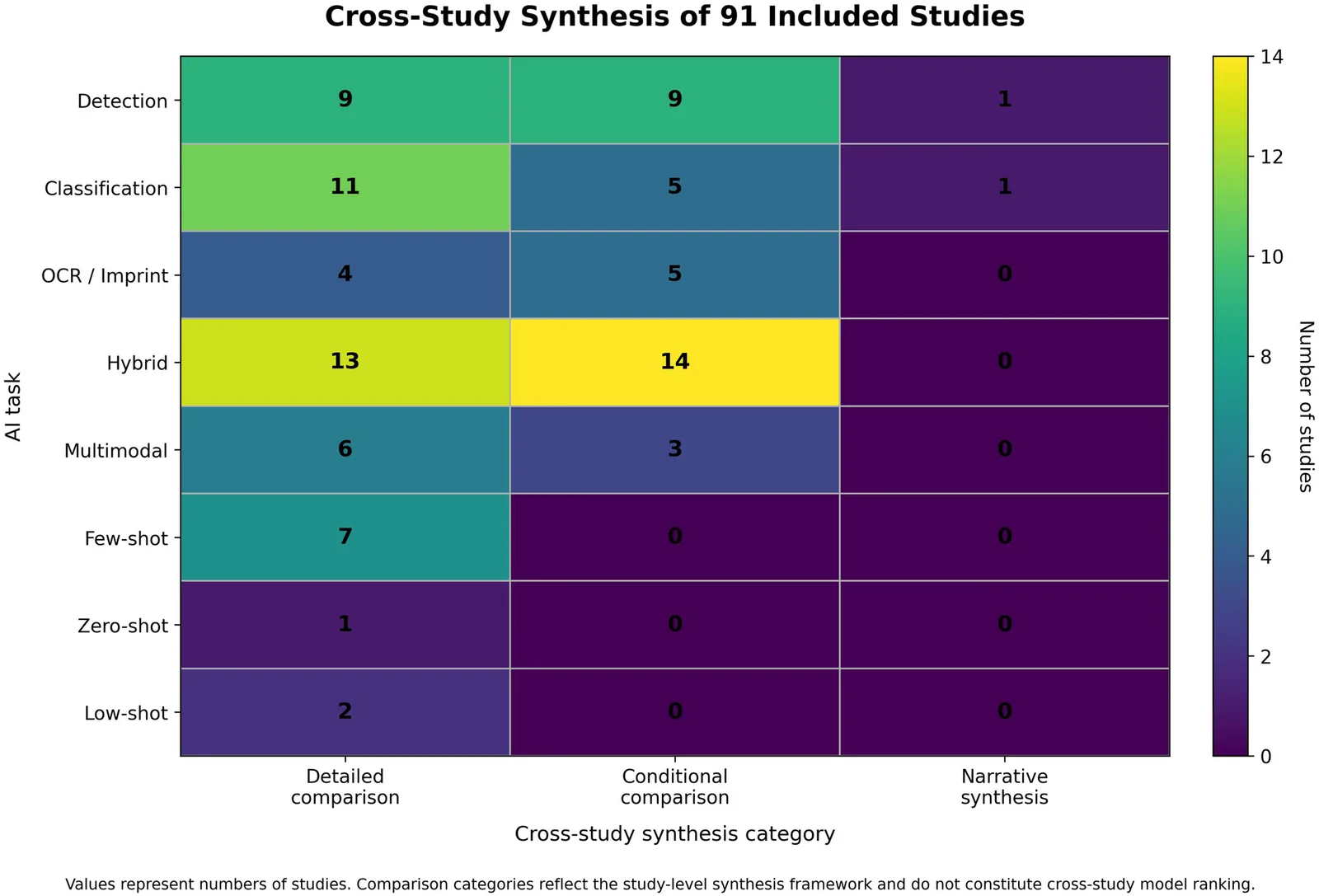
Cross-study synthesis visualization.

## 3 Foundations of AI-based medication identification and digital health

### 3.1 Medication identification as a computer vision and health informatics problem

Medication identification sits at the intersection of computer vision, artificial intelligence, and health informatics. Accurate recognition can help reduce medication-identification errors and support safer medication use, particularly for older adults, visually impaired individuals, and patients with limited health literacy (4). Traditional medicine verification is mostly manual and depends on visual attributes including shape, color, size, and imprint, which can be time-consuming and susceptible to human error. AI combined with digital imaging has thus enabled the development of automated medication recognition systems that enhance the efficiency and reliability of medication identification, as detailed in Alahmadi et al. (4) and Ponte et al. (12).

From a computer vision perspective, the problem of medication recognition is typically described as a fine-grained recognition problem because many medications look very similar. The variations in illumination, viewing angle, image quality, background complexity, and imprint visibility (13) can cause changes to the recognition performance. As a solution to these problems, recent works explored incorporating visual features such as shape, color, texture, contours, and imprinted text into deep learning, image preprocessing, and optical character recognition (OCR) techniques to achieve better recognition performance under different imaging conditions (12–14).

In addition to image analysis, medication identification can be approached using a health informatics perspective, for example by connecting the recognition output to a medication database, information retrieval system, language-based interface, or mobile health application. Such integration can facilitate medication verification, access to medication information, and user-centered medication assistance through text and/or speech interfaces (4, 14). In this way, the integration of computer vision, OCR, multimodal AI, and digital health technologies does not just support medication recognition but also medication-information and medication-management workflows, although their practical and clinical use depends on the validation, deployment conditions, usability, safety, and appropriate integration of these technologies (4, 12–14).

### 3.2 Multi-attribute learning for pharmaceutical identification

Pharmaceutical identification has, in recent years, shifted increasingly toward the joint analysis of multiple visual and textual characteristics, moving away from treating a medication as a single visual object. Many pharmaceuticals share similar shapes, colors, and other physical features, making reliable identification difficult when based on any single attribute alone. To overcome this drawback, multi-attribute learning techniques that employ complementary attributes like shape, color, texture, imprint characteristics, and information related to the medication are being studied for improving the recognition performance and robustness of the medication-recognition system (3, 12).

With the development of deep learning, the properties of complementary medicines have been learned and integrated into a single recognition framework of pharmaceuticals, further supporting multi-attribute pharmaceutical recognition. Different hybrid methods, including shape and color features, type of medication, and metadata verification, have been studied as a method for increasing the confidence and robustness of medication recognition (3). Likewise, image processing, optical character recognition (OCR), and feature extraction-based computer vision systems have been employed to detect relevant pharmaceutical properties, such as in verification and medication dispensing systems (12).

The emergence of the significance of pill-imprint identification has also led to the development of multimodal learning frameworks. These frameworks may be used together with image classification, OCR, language-based processing, and retrieval mechanisms to collectively interpret the visual appearance and imprinted text, which can be leveraged to identify a medication, retrieve the medication information, and recognize when more complex or unfamiliar conditions arise (14). These types of methods can be particularly useful where the packaging or labelling of medicines is not available, and the only certain information can be obtained from the medicine itself.

Overall, multi-attribute learning is a promising area in medication recognition using AI, where multiple pharmaceutical attributes work together in a cohesive model. These methods have the potential to enhance the robustness of recognition and to extend the application to medication verification and digital-health scenarios by integrating the visual, textual, and contextual information. However, their practical and clinical utility depends on factors other than simply recognition performance: knowledge of the diversity of the datasets, the method used for validation, generalizability, usability, and safety (3, 12, 14).

### 3.3 Medication safety and clinical relevance of medication recognition

Medication recognition constitutes a computer vision problem that has received increasingly focused attention in the context of medication safety and digital-health applications, even as image classification remains a well-established problem within computer vision more broadly. Accurate medication identification can support medication verification and contribute to safer medication-use processes, particularly for older patients managing multiple medications or complex treatment regimens (15, 16). Medication-identification errors, in turn, can lead to medication-administration errors, medication-management difficulties, and adverse drug events (ADEs). AI-based medication recognition has therefore emerged as a promising digital-health approach for supporting the automated identification, verification, and monitoring of medications (15, 17).

Recent advances in artificial intelligence and computer vision have enabled the development of automated systems capable of detecting and identifying tablets and capsules under varying imaging conditions. Such systems can reduce reliance on manual visual inspection and have been explored across settings including pharmaceutical manufacturing, quality assurance, medication verification, and automated monitoring. Detection and recognition methods have been investigated, including approaches based on the YOLO architecture. Medication-monitoring systems that pair recognition with intelligent assistance have also been explored as a means of supporting medication adherence and assisting caregivers of older adults with medication-related tasks (15). Whether such systems meaningfully improve adherence or reduce medication errors, however, depends on system design, validation setting, usability, and evidence of successful real-world deployment.

Medication recognition can additionally serve as one component of a broader patient-facing healthcare or digital-health solution. For some systems, recognition correlates with reminder generation, medication-information retrieval, adherence monitoring, or other medication-management tasks. OCR-based methods can be used to extract and retrieve medication information from labels and/or imprints, which could help with medication management processes for older adults, limited health literacy patients, and multilingual patients (16). The basic recognition task is only part of the process for more complex functions such as drug-interaction checking, personalized guidance, and clinical decision support, which generally involve connecting to other medication databases, knowledge resources, or health systems.

Automated medicine identification can be used as an enabler in medicine identification, medicine management, and digital-health applications as a whole. However, there are other factors that are equally important in determining the contribution of recognition performance to medication safety and patient-centered care, such as the quality of the datasets, validation, generalizability, usability, deployment context, and safety considerations (15–18).

### 3.4 Challenges in real-world medication recognition

Despite significant improvements in AI and computer vision, real-world conditions make reliable medication identification challenging. In these applications, the environment in which images are taken may vary significantly from the controlled environments used in the construction of the databases, including illumination, background complexity, camera characteristics, viewing angle, occlusion, and imaging noise. These factors can introduce errors in recognition performance and reduce the ability to transfer recognition models from their training to new conditions. Significant advances have been achieved in the field of deep learning and in the combination of deep learning and hybrid recognition methods, but there are still challenges in the field, such as robustness to varying imaging conditions, scalability, diversity of datasets, and the lack of standardized evaluation methods (19).

A significant problem is the disparity in performance between experimental and real-world use. Many variations of images captured by mobile medication-recognition systems are not included in the training sets, and they are user-generated. In recent years, research has been conducted on the following methods that aim to increase robustness to these variations: multimodal feature extraction, shape analysis, imprint analysis, model compression, and data augmentation. Despite this, performance on large-scale pill-recognition tasks is often found to be lower when using consumer-captured images compared to standardized reference images, further highlighting the need for assessing recognition systems under realistic imaging conditions, as opposed to idealized conditions (20, 21).

Additional problems arise if the system of medication recognition is considered for practical or healthcare deployment. Performance of automated and code platforms may differ widely across datasets and in healthcare contexts; therefore, no single platform or approach can be expected to perform optimally in all settings and for all use cases. Data quality, computational resources, system complexity, and usability also influence the practical implementation (22).

These challenges collectively highlight the importance of more powerful, scalable, and well-validated medication-recognition systems, ones that grow from larger and more representative datasets, standardized benchmarking protocols, and comprehensive testing in realistic settings. This advancement is vital to establishing the viability of integrating AI-powered medication recognition into digital-health systems without compromising performance, user-friendliness, and security (19, 21, 22).

### 3.5 Medication recognition pipeline and digital health system architecture

Modern medication recognition systems can be implemented as end-to-end digital health pipelines that transform visual information about medications into identification outputs and, in some systems, medication-related information for end-users. Current architectures may combine image acquisition and preprocessing, pill detection and feature extraction, pill classification and retrieval, verification, and image-based information delivery, rather than relying on image classification alone. Integrated pipelines of this kind can support medication identification and, when linked to appropriate information resources, enable downstream medication-related functions as well (23). Such architectures therefore provide a basis for embedding medication recognition within broader digital-health systems.

The image acquisition phase of a typical medication recognition pipeline may involve capturing images through a smartphone, wearable camera, or clinical imaging system. The image-enhancement phase applies techniques intended to improve image quality and recognition robustness, while image segmentation is used to isolate the relevant medication region. Feature-extraction methods then derive characteristics from the processed images to support recognition (23, 24). The shape, color, and texture of the medication, along with the imprint details, can then be analyzed with AI and deep learning models. Some also include retrieval techniques to compare extracted features to information in pharmaceutical reference databases to support identification even when there is no packaging or labelling available (23).

For example, AI-based medication recognition has been extended into digital-health applications, medication reminders, explainable AI, accessibility, and assistive technologies with the supplementary use of speech-to-text (STT) and text-to-speech (TTS) (25), showcasing a medication recognition system that is accessible to people with visual impairments. Such systems can enhance user independence by providing information related to medication via user-friendly interfaces, but the practical utility of these systems will be dependent on the level of recognition reliability, usability, and deployment context (24, 25).

From a health informatics standpoint, medication recognition systems can integrate computer vision with pharmaceutical databases, medication-information resources, and user-centered interfaces. This kind of integration lays the groundwork for medication-management applications spanning medication identification, information access, reminders, and other patient-oriented functions across both clinical and community settings (23–25). Downstream functions such as medication safety support, adherence assistance, or clinical decision support, however, require appropriate integration with additional information resources and should be considered distinct from the medication-recognition task itself.

## 4 Taxonomy of AI-based medication recognition systems

### 4.1 Detection-based approaches

Detection-based approaches, as shown in Figure 9, comprised 19 of the 91 included studies and represented a major methodological direction in AI-based medication recognition. These approaches primarily focused on localizing pills, tablets, capsules, medication packages, or relevant visual regions using object-detection architectures, either as the principal recognition mechanism or as an initial stage in a subsequent identification pipeline. The reported approaches included YOLO-family detectors, Mask R-CNN, RetinaNet, SSD, Faster R-CNN, and customized lightweight detection architectures. Representative examples included YOLOv8 with learnable multi-scale feature fusion for pill detection (26), customized YOLOv8-based recognition (27, 28), Mask R-CNN-based pill-shape localization (29), and deep-learning-based medication detection systems (30). Other studies investigated counterfeit medication detection (31), multi-pill detection using feature augmentation and automatic labeling (32), capsule and tablet detection (18, 33), lightweight real-time pill detection (34), and computer-vision-based medication detection for adherence-oriented applications (15, 35). These studies illustrate the use of detection architectures across different medication forms and imaging conditions.

**Figure 9:**
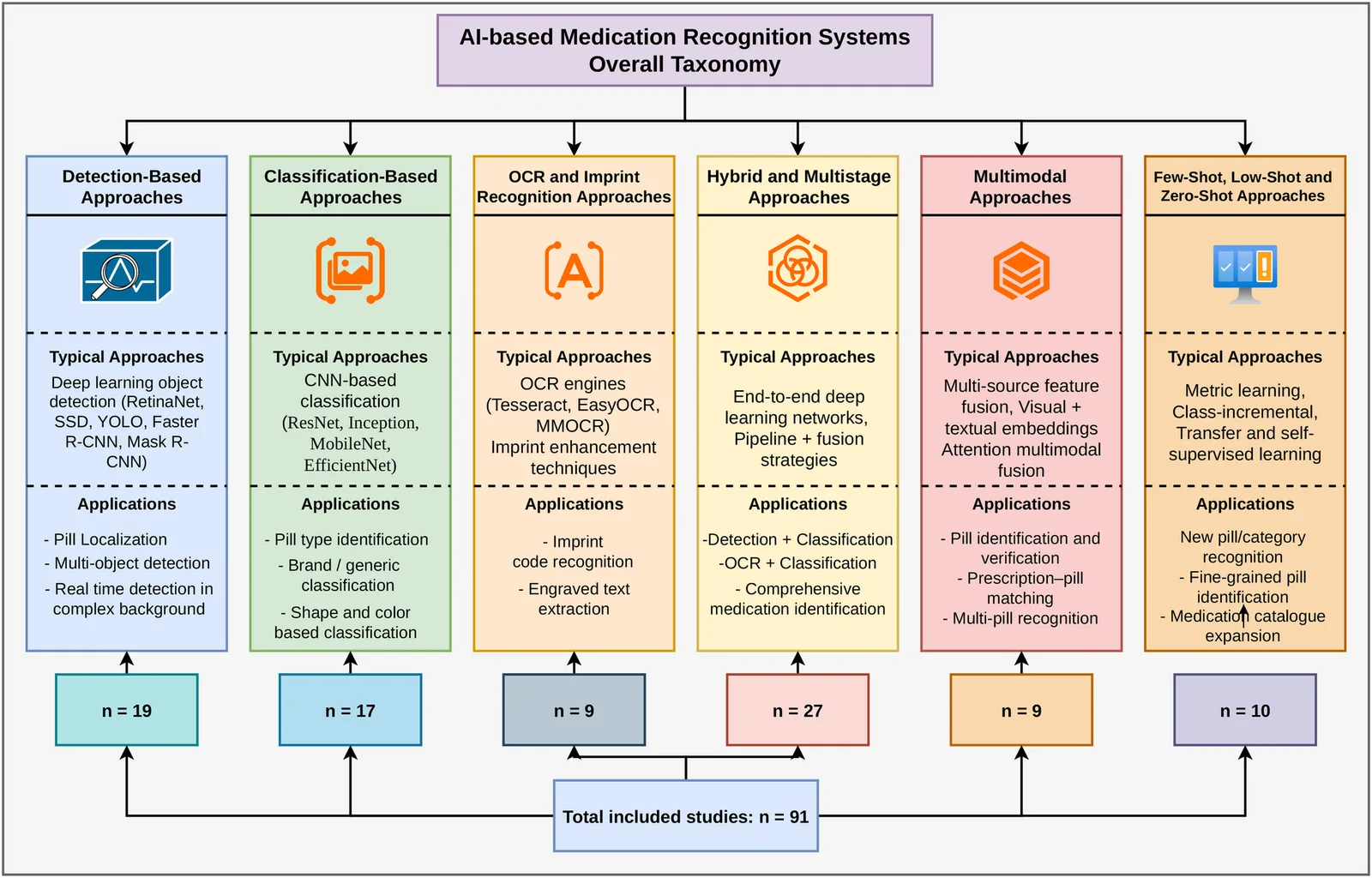
Overall taxonomy of AI-based medication recognition systems. For analytical counting, each study was assigned to one primary methodological family, although individual studies could incorporate additional computational components. systems.

The detection literature also included approaches designed for unconstrained or application-oriented settings. Examples included wearable smart-glasses-based pill recognition (36), elderly-oriented medication recognition (4), smart pill detection (17), unconstrained pill-shape recognition (37), and deep-learning-based detection of medicine-name regions (38). Multicenter real-world recognition (8) and comparative evaluation of RetinaNet, SSD, and YOLOv3 (39) further illustrate the diversity of detection settings and methodological configurations. Across the 19 studies, detection was therefore used both as a standalone recognition strategy and as a localization component within broader recognition pipelines. Differences in imaging conditions, medication categories, dataset composition, and evaluation protocols indicate that these studies represent distinct methodological implementations rather than directly interchangeable detection systems. Consequently, the taxonomy identifies detection as a methodological category without implying a universal performance hierarchy among the architectures.

### 4.2 Classification-based approaches

Classification-based approaches, as shown in Figure 9, comprised 17 of the 91 included studies and focused primarily on assigning medication images to predefined pill, tablet, drug, or shape categories. These studies formulated medication recognition as an image-classification problem, using convolutional neural networks (CNNs), transfer-learning architectures, ensemble methods, and related deep-learning models to learn discriminative visual representations. Representative approaches included Pill-MSVAE for automatic drug identification (40), AI-based pill recognition designed for visually impaired users (25), and a deep-learning pharmaceutical recognition system for mobile medication identification (24). Other studies investigated pharmaceutical pill identification using MobileNet, ResNet50, and related architectures (41), round-pill shape recognition using deep learning (42), and transfer-learning-based drug classification (43).

Several studies incorporated additional strategies to address challenges encountered in medication image classification. These included ensemble learning for class-imbalance handling (44), approaches addressing illumination variation during pill identification (45), and recognition systems incorporating voice assistance for visually impaired users (46). Stacking-based classification using ResNet50, Inception-V3, and MobileNet was investigated in Ahammed et al. (47), while capsule-network-based tablet identification was examined in He and Zhu (48). Mobile-oriented pill image classification was further explored in Mehmood et al. (49), and a deep-learning approach incorporating Faster R-CNN was categorized within the classification evidence in Boonthep et al. (50).

Additional studies examined CNN-based tablet classification under controlled feeding conditions (51), deep-learning-based drug identification using medical-center data (52), smartphone-based pill recognition under varying imaging conditions (53), and fine-grained pill identification using a deep convolutional network (54). Collectively, these studies demonstrate the use of classification-based approaches across different medication forms, visual characteristics, imaging environments, and application settings. The classification category therefore captures studies in which assigning medication images to predefined recognition classes constitutes the primary methodological direction, while differences in datasets, architectures, and evaluation protocols are considered separately in the subsequent comparative synthesis.

### 4.3 Imprint recognition and OCR-based approaches

OCR and imprint recognition approaches, as shown in Figure 9, comprised nine of the 91 included studies, representing a methodological category in which medication identity was derived primarily from textual or imprint information associated with pills, tablets, blister packages, or medication packaging. These studies employed optical character recognition and related text-processing techniques either as the principal recognition mechanism or as a complementary component within broader medication-recognition pipelines. The approaches therefore ranged from dedicated pill-imprint recognition to OCR-assisted identification of medication names, dosages, and other textual information.

A representative dedicated imprint-recognition approach was GO-PILL (55), which employed geometry-aware preprocessing, imprint localization and rectification, and an OCR architecture based on ABINet to recognize printed, debossed, curved, diagonal, and linear pill imprints. The study illustrates how geometric variation in medication markings can be incorporated into an OCR-based recognition pipeline. Other studies combined visual recognition with OCR for tablet identification and medication-information extraction (56), investigated OCR-based real-time medication identification (57), and integrated CNN-based visual processing with OCR for tablet recognition (58). These approaches demonstrate the use of textual information as an additional discriminative source when visual appearance alone may be insufficient.

OCR-based recognition was also applied to medication packaging and mobile-oriented identification systems. OptiRecDrug (59) used PaddleOCR and EasyOCR to identify drugs from photographs of packaging and labels, while other studies investigated imprint-based recognition (60), OCR combined with text-detection components such as CRAFT and Keras-OCR for blister-package images (61), and OCR-CNN configurations combined with object-detection components for image-based drug recognition Ajhari et al. (62). Another study incorporated textual and visual medication information within a deep-learning-based automatic pill-identification framework (14). These studies illustrate the use of OCR across both pill-surface imprints and medication-package text.

Collectively, the OCR and imprint-recognition studies demonstrate that textual information can complement visual characteristics in medication identification. However, OCR-stage performance should not be interpreted as equivalent to end-to-end medication-identification performance, because the two represent different stages of the recognition process. Differences in imprint characteristics, image preprocessing, text-detection procedures, dataset construction, and evaluation protocols also limit direct comparison across studies. Accordingly, this category represents a distinct methodological direction within AI-based medication recognition rather than a performance hierarchy among OCR systems.

### 4.4 Hybrid and multistage recognition approaches

Hybrid and multistage approaches, as shown in Figure 9, comprised 27 of the 91 included studies and represented the largest methodological family in the taxonomy. These systems integrated two or more complementary processing stages, models, feature representations, or decision mechanisms to perform medication recognition. Examples included YOLO-based detection and classification pipelines (63, 64), combinations of deep-learning models with feature-based processing (65), and integrated detection, classification, and small-region analysis for medication identification (66, 67). Other studies combined object localization with clustering or additional recognition modules (68–70), while application-oriented systems incorporated monitoring, retrieval, or supporting processing stages alongside recognition (23, 71–73).

Several studies implemented explicit sequential pipelines in which medication regions were first detected or segmented and subsequently classified or identified (12, 20, 74, 75). Such architectures were also used for automated pill identification, incorporating multiple CNNs, preprocessing, feature extraction, or complementary recognition components (76–78). More integrated frameworks combined detection with multi-attribute classification and medication metadata retrieval (3), deblistering or dispensing-oriented processing (79), and attention-based or uncertainty-aware recognition components (80). Mobile medication identification systems further combined object detection, image classification, and OCR/text recognition to support medication identification workflows (81).

Hybrid architectures also included improved detection pipelines and systems combining multiple recognition stages or evaluation components (82–85). For example, the NLM-based medication-identification studies integrated localization with deep CNN recognition, while the lightweight framework combined pill detection, multi-attribute recognition, OCR-based imprint analysis, and metadata retrieval.

From a digital-health perspective, these multistage architectures are relevant because they can connect medication image acquisition with identification, information retrieval, monitoring, verification, or medication-management functions. However, the hybrid category represents methodological organization rather than a performance hierarchy, as the individual systems differ substantially in task definition, datasets, evaluation protocols, and intended digital-health applications.

### 4.5 Multimodal medication recognition approaches

Multimodal medication-recognition approaches, as shown in Figure 9, comprised nine of the 91 included studies (13, 86–93). These systems extend image-based recognition by integrating complementary information sources, including prescription context, textual or imprint information, multiple visual representations, packaging information, or language-based models. The resulting approaches aim to reduce reliance on a single visual representation and support medication identification within more information-rich digital-health workflows.

Nguyen et al. (86) proposed PIKA, which combined CNN-derived pill-image features with prescription-derived medical knowledge graphs, graph embeddings, and context attention for contextual pill recognition. The system therefore linked visual medication appearance with prescription relationships rather than relying solely on image similarity. Similarly, Hsu and Su (89) combined YOLOv4-based pill identification with prescription confirmation encoded through QR information, supporting medication verification on an embedded platform. Chen et al. (92) integrated deep-learning image classification with barcode/QR-based prescription information within an automated dispensing workflow, using MobileNet V1 and Inception V3 for medicine verification.

Other studies incorporated textual information directly into visual recognition. My et al. (88) fused RGB, contour, texture, and OCR-derived imprint information using a Vision Transformer, while Rádli et al. (91) combined multiple visual streams with word embeddings derived from medication information leaflets. Nguyen Thi My et al. (13) integrated RGB, contour, texture, and imprinted text using lightweight attention and OCR components for edge-based recognition. Menaha et al. (90) further demonstrated multimodal drug identification using medication images, drug-name information, and a multimodal Gemini-based model.

More comprehensive multimodal systems were reported by Balahariraj et al. (87) and Nguyen et al. (93). PGPNet-X combined visual recognition, OCR, packaging/logo information, prescription verification, gated multimodal fusion, and graph reasoning, whereas Nguyen et al. (93) incorporated multimodal data fusion with graph-based reasoning for multi-pill recognition.

Collectively, these studies demonstrate how multimodal recognition can connect medication appearance with textual, prescription, contextual, and relational information, supporting more information-rich digital-health and medication-management applications. However, the studies differ substantially in modalities, fusion strategies, tasks, and deployment settings; therefore, this category represents a methodological direction rather than a universal performance hierarchy.

### 4.6 Few-shot, low-shot and zero-shot approaches

Few-shot, low-shot, and zero-shot approaches, as shown in Figure 9, comprised 10 of the 91 included studies and represent learning paradigms designed to address medication recognition when labeled examples are limited or when previously unseen pill categories must be recognized. Seven studies were categorized as few-shot, two as low-shot, and one as zero-shot.

Few-shot approaches investigated recognition with only a small number of labeled examples per category. The original Few-Shot Pill Recognition study (94) employed multi-stream deep learning using RGB, texture, contour, and imprint information and evaluated category-disjoint few-shot settings on CURE and NIH/NLM datasets. Self-supervised few-shot learning was investigated using AMDIM and Prototypical Networks (95), while Multi-Stream Pill Recognition with Attention (96) used multi-stream feature representations and self-attention. Metric-based recognition using textual and visual cues was examined in Rádli et al. (97), and transfer learning with Prototypical Networks and ResNet-101 was evaluated in Chee et al. (98). Few-shot class-incremental recognition was further addressed by Zhang et al. (99, 100) using FCPill and CURE-derived datasets.

Low-shot approaches focused on recognition with extremely limited reference information. Wang et al. (101) investigated pill recognition using minimal labeled data, whereas the ePillID benchmark (102) addressed fine-grained pill identification using limited consumer-image examples across thousands of pill types.

The zero-shot study (103) introduced Zero-PIMA for pill–prescription matching, combining pill-image detection with prescription text, graph representations, and contrastive learning to recognize unseen pill categories.

Collectively, these approaches are particularly relevant to digital-health medication-recognition systems because they address the practical challenge of adding or identifying medications for which extensive labeled image collections may not be available.

## 5 Deep learning architectures and computational intelligence techniques

### 5.1 Convolutional neural networks

Convolutional neural networks (CNNs) are widely used deep learning architectures in medication recognition because they can automatically learn hierarchical visual representations from pharmaceutical images. Unlike conventional machine-learning approaches that may rely on hand-crafted features, CNNs can learn discriminative representations of characteristics such as shape, colour, texture, contour, and imprint information. These properties make CNN-based architectures applicable to pill classification, medication verification, and image-based medication recognition tasks.

In medication recognition, CNNs progressively learn visual features ranging from low-level characteristics such as edges and contours to higher-level representations that support discrimination between visually similar medications. CNN-based approaches have therefore been explored in various recognition applications such as classification of pills, identification of medication, and verification-related tasks. Their usage serves as a basic visual processing element, which can then be followed by a detection module, an OCR module, a retrieval module, or another computational module.

CNN-based recognition is extended by transfer learning, attention mechanisms, and multimodal feature fusion in recent studies. Transfer learning can be used to help with recognition in situations where there are few medication datasets or labelled examples, and attention mechanisms can highlight informative regions or features in medication images. Recent multimodal CNN-based architectures have also combined complementary image information such as visible-light and infrared images to tackle difficult imaging situations. These extensions demonstrate how CNNs can be used in more complex pipelines for recognizing medication, but not just as a standalone medication recognizer.

CNN-based architectures are also applicable to digital-health medication-recognition workflows. They’ve been used in mobile, embedded, OCR-based, and medication-information-retrieval systems as a visual-processing layer, which can help with medication identification and verification. The integration of complementary technologies provides opportunities for applications including patient accessible medication information systems, medication-dispensing verification, and medication identification. The evidence, however, is still diverse with respect to datasets, computational demands, interpretability and ground truth validation; therefore, CNN architecture should not be assumed to be clinically effective or ready for deployment.

### 5.2 Object detection architectures

Object detection architectures formed an important computational component of the medication-recognition systems reviewed in this study. Unlike image-classification architectures that generally operate on a pre-cropped medication region, object detectors jointly estimate the location and class of medication objects, making them suitable for images containing multiple pills, capsules, blister-packaged medicines, or visually complex backgrounds. The detection studies employed both two-stage and single-stage architectures, including Faster R-CNN and Mask R-CNN, as well as SSD, RetinaNet, and YOLO-family detectors (18, 29, 34, 39).

Two-stage architectures were used where region proposal, localization, and object refinement were important. Mask R-CNN-based pill-shape localization was investigated in An et al. (29), while other detection pipelines incorporated region-based processing for medication localization. Single-stage architectures were more frequently adapted for efficient medication detection. YOLO-based systems included customized YOLOv8 detection with learnable feature fusion (26), customized YOLOv8-based recognition (27), lightweight real-time detection (34), and YOLO-based counterfeit medication detection (31). Multi-pill detection and automated labeling were also investigated using YOLO-based architectures (32). Comparative evaluation of RetinaNet, SSD, and YOLOv3 was reported in Tan et al. (39).

Architectural modifications increasingly focused on feature representation, computational efficiency, and adaptation to medication-specific visual characteristics. These included learnable multi-scale feature fusion, adaptive feature sampling, lightweight convolutional components, pretrained backbones, and feature-pyramid or cross-scale processing. Such modifications are relevant to medication images containing small, overlapping, or visually similar objects and to applications requiring rapid inference (26, 27, 34, 39).

From a digital-health perspective, object detection architectures can provide the localization component required for medication-recognition workflows operating on unconstrained images, multiple-medication scenes, wearable or mobile image acquisition, and real-world clinical or community settings (4, 8, 15, 36, 37). However, the reviewed architectures were evaluated under different datasets, medication categories, imaging conditions, and validation protocols; therefore, their reported results should be interpreted within their respective experimental contexts rather than used to establish a universal ranking of detection architectures.

### 5.3 Classification architectures

Classification architectures were used to assign medication images to predefined pill, tablet, drug, or shape categories. The reviewed studies primarily employed convolutional neural networks (CNNs), transfer-learning models, ensemble architectures, and specialized deep-learning networks. Pill-MSVAE combined CNN, Vision Transformer, and variational autoencoder components for automatic pill recognition (40), while other CNN-based systems addressed medication recognition for visually impaired users and mobile applications (24, 25). Transfer-learning architectures based on models such as MobileNet and ResNet were also investigated for pharmaceutical pill identification (41, 43), while deep convolutional architectures were applied to round-pill and fine-grained medication recognition (42, 54).

Several studies modified conventional CNN classification through ensemble and multi-network architectures. Ensemble learning was investigated to address medication-class imbalance (44), while a stacking architecture integrating ResNet50, Inception-V3, and MobileNet was developed for pill classification (47). These approaches illustrate the use of complementary feature representations from multiple pretrained or independently trained networks. Capsule-network-based classification was additionally investigated for tablet identification (48), providing an alternative representation-learning architecture.

Classification architectures were also adapted for variable imaging and deployment conditions. Illumination-aware recognition was examined to improve robustness to changes in image appearance (45), while mobile-oriented classification systems were investigated for medication recognition on portable platforms (49). A Faster R-CNN-based deep-learning approach was categorized within the classification evidence in the study-level taxonomy (50). CNN-based classification was further evaluated for controlled tablet-feeding conditions (51), medical-center medication data (52), and smartphone images acquired under varying conditions (53).

Overall, the classification evidence demonstrates architectural progression from conventional CNNs toward transfer learning, ensemble, capsule, and deployment-oriented architectures, while differences in datasets, class definitions, and evaluation protocols require architecture-level comparisons to remain study-specific.

### 5.4 OCR and sequence-based recognition

OCR and sequence-based architectures were employed to extract medication imprints, names, and other textual information from pill surfaces and medication packaging. These architectures typically combine text localization, image rectification, visual feature extraction, character recognition, and sequence modeling rather than treating medication recognition as a conventional image-classification problem. GO-PILL (55) represents a geometry-aware architecture that integrates imprint refinement, localization and rectification, TextSnake-based processing, and MMOCR/ABINet. Its ABINet component combines a ResNet-based visual encoder with a Transformer language model and a fusion module for imprint recognition.

Other studies adopted conventional and deep-learning OCR pipelines. Tesseract and Keras-OCR were investigated for medication-text recognition, with word-level correction incorporated to improve textual recognition (56), while real-time medication identification using OCR was explored in Sony et al. (57). CNN-based visual processing was combined with OCR for tablet recognition and medication-information extraction in Pujari et al. (58), illustrating an architecture in which visual classification and textual recognition operate as complementary components.

Text-detection and OCR architectures were also adapted to medication packaging. OptiRecDrug (59) incorporated PaddleOCR and EasyOCR for recognition from package and label photographs. Imprint-based deep-learning recognition was investigated in Pilania et al. (60), whereas Maitrichit and Hnoohom (61) combined text-detection and OCR components, including CRAFT and Keras-OCR, for blister-package images. Ajhari et al. (62) integrated OCR and CNN processing with object-detection components, demonstrating a multistage architecture for image-based drug recognition. Textual and visual medication information were further incorporated into a deep-learning pill-identification framework (14).

These architectures are relevant to digital-health medication-recognition workflows because textual information can complement visual appearance when medication identity depends on imprints or package information. However, OCR accuracy and end-to-end medication-identification performance represent different evaluation stages and should therefore be interpreted separately.

### 5.5 Hybrid and end-to-end framework architectures

Hybrid and end-to-end frameworks integrate multiple computational components or sequential processing stages within a unified medication-recognition workflow. Unlike single-stage architectures, these systems may combine object detection, image classification, segmentation, feature extraction, OCR, retrieval, or decision modules to address different stages of medication identification and verification. YOLO-based detection followed by classification was used in several medication-recognition pipelines (63, 64), while other frameworks combined multiple deep-learning models with feature-based processing or localized image analysis (65–67).

Sequential architectures were also developed in which medication regions were first localized or segmented and subsequently classified or identified (12, 20, 68–70, 74, 75). Other frameworks integrated multiple CNNs, preprocessing and feature-extraction stages for automated pill identification (76–78). These architectures demonstrate how complementary computational modules can be arranged to address differences in medication appearance, object localization, and recognition requirements.

More application-oriented end-to-end frameworks incorporated medication attributes, metadata, or downstream verification components. Detection was combined with multi-attribute classification and medication-information retrieval (3), while dispensing-oriented systems integrated recognition with medication-handling workflows (79). Attention-based and uncertainty-aware components were incorporated into other recognition frameworks (80), and mobile-oriented systems combined detection, classification, and OCR within a unified medication-identification workflow (81). Additional integrated architectures included enhanced detection and recognition pipelines (82–85).

From a digital-health perspective, hybrid and end-to-end architectures are relevant because they can connect image acquisition with medication localization, identification, information retrieval, verification, or dispensing support. However, architectural complexity does not by itself establish superior performance; differences in datasets, task definitions, evaluation protocols, and deployment conditions require each framework to be interpreted within its study-specific context.

### 5.6 Multimodal architectures

Multimodal architectures integrate complementary information sources within medication-recognition pipelines to provide representations beyond a single visual input. The reviewed architectures combined medication images with textual, imprint, prescription, packaging, contextual, or relational information, while other systems fused multiple visual representations. PIKA (86) combined CNN-derived pill-image features with medical knowledge-graph representations and contextual attention, linking visual medication characteristics with prescription-related information.

Several architectures incorporated prescription and textual information directly into the recognition process. Hsu and Su (89) combined YOLOv4-based pill identification with QR-based prescription confirmation in an embedded system, while iDispensing (92) integrated deep-learning image classification with barcode/QR-based prescription information for automated dispensing verification. My et al. (88) fused RGB, contour, texture, and OCR-derived imprint representations using a Vision Transformer, demonstrating multimodal visual and textual feature integration. Similarly, Rádli et al. (91) combined multiple visual streams with word embeddings derived from medication information leaflets, using attention-based metric learning for pill recognition.

Lightweight multimodal architectures were also investigated for resource-constrained deployment. Nguyen Thi My et al. (13) combined RGB, contour, texture, and imprint information using lightweight attention and OCR components for edge-device recognition. Menaha et al. (90) further explored multimodal drug identification using a Gemini-based generative AI framework incorporating medication image and textual information.

More comprehensive fusion architectures were reported in Balahariraj et al. (87) and Nguyen et al. (93). PGPNet-X (87) combined visual recognition, OCR, packaging and logo information, prescription verification, gated multimodal fusion, and graph reasoning. Nguyen et al. (93) similarly incorporated graph-neural-network-assisted multimodal data fusion for multi-pill recognition.

Overall, multimodal architectures demonstrate how heterogeneous medication information can be integrated for identification, verification, and medication-management workflows. However, differences in modalities, fusion mechanisms, tasks, and deployment settings require architectural comparisons to remain study-specific rather than interpreted as a universal performance hierarchy.

### 5.7 Few-shot, low-shot and zero-shot learning architectures

Few-shot, low-shot, and zero-shot learning architectures address medication recognition under limited labeled-data conditions or when recognition must extend to previously unseen medication categories. The reviewed architectures employed metric learning, prototypical learning, self-supervised representation learning, transfer learning, and class-incremental learning mechanisms. The Few-Shot Pill Recognition framework (94) used multi-stream visual representations incorporating RGB, texture, contour, and imprint information for category-disjoint few-shot recognition. Self-supervised pretraining followed by Prototypical Networks was investigated in Cordeiro et al. (95), while a multi-stream architecture with attention and metric learning was evaluated in Rádli et al. (96). Textual and visual representations were further integrated within metric-based learning in Rádli et al. (97), and transfer learning with Prototypical Networks and ResNet-101 was explored in Chee et al. (98).

Class-incremental architectures were developed to support recognition as new medication categories are introduced. Zhang et al. (99) proposed a forward- and backward-compatible few-shot class-incremental framework, while Zhang et al. (100) investigated a well-prepared few-shot class-incremental architecture using FCPill and mCURE datasets. These architectures are relevant to medication-recognition systems that may need to accommodate expanding medication categories without requiring extensive retraining data.

Low-shot architectures addressed recognition with minimal reference information. Wang et al. (101) employed a GoogLeNet/Inception CNN ensemble with limited labeled pill images and synthetic training data, whereas the ePillID benchmark (102) investigated fine-grained pill identification under limited-reference conditions.

The zero-shot architecture reported in Nguyen et al. (103) combined pill-image detection, prescription-text representation, graph-based learning, and contrastive learning for pill–prescription matching involving unseen categories. Collectively, these architectures demonstrate alternative strategies for reducing dependence on large labeled medication-image collections, with potential relevance to scalable digital-health medication-recognition systems.

## 6 Comparative analysis of existing studies

### 6.1 Overview of included studies

The systematic evidence base comprised 91 studies that met the predefined eligibility criteria for AI-based medication recognition. These studies addressed 19 detection, 17 classification, 9 OCR/imprint-recognition, 27 hybrid, 9 multimodal, 7 few-shot, 1 zero-shot, and 2 low-shot approaches. Each study was assigned a single primary AI task for analytical organization, although individual studies could still incorporate multiple computational components within their recognition pipelines.

To support meaningful cross-study interpretation, the included studies were assessed at full text for their methodological and evaluation characteristics. This assessment considered the primary medication-recognition task, identifiable model or methodological family, dataset and dataset size, quantitative performance reporting, evaluation procedures, validation and generalizability, and other methodological characteristics captured through the D1–D8 assessment framework. Based on the completeness and comparability of the reported methodological, dataset, evaluation, and performance evidence, 53 studies provided sufficient information to support structured within-task comparison.

A further 36 studies were classified as conditionally comparable, since limitations in the available methodological, dataset, or evaluation information restricted direct numerical interpretation. These studies were retained for supplementary and contextual synthesis. Two additional studies were retained for narrative synthesis, as the available evidence did not support structured cross-study comparison. All 91 studies therefore contributed to the overall evidence base, though the depth of comparative interpretation depended on the available full-text evidence.

For concise presentation, 34 representative studies were selected on the basis of methodological, dataset, evaluation, and model-comparison diversity, rather than reported performance alone. Thirty-three of these were drawn from the structured comparison pool, while one additional detection study was retained from the conditionally comparable group because it offered an explicit within-study comparison of multiple detection architectures, despite a lower D5 generalizability rating. This approach preserved diversity in datasets, experimental conditions, methodological configurations, and evaluation settings, while limiting redundancy within the main-text tables. The complete structured comparison pool and the conditionally comparable studies were retained in the supplementary evidence. The resulting comparison framework covered all 91 studies, with no overlap among the structured, conditional, and narrative synthesis groups.

Within the structured-comparison pool, representative studies were purposively selected to ensure coverage of the major AI-task categories, methodological families, dataset characteristics, evaluation strategies, and validation settings, while minimizing redundancy among studies with closely similar experimental configurations. Selection was not based on the magnitude of reported performance.

Figure 10 summarizes the coverage of studies used for structured within-task model/method comparison. All studies in the smaller few-shot, zero-shot, and low-shot groups were retained, as were the OCR/imprint studies meeting the comparison criteria, whereas representative subsets were drawn from the larger detection, classification, hybrid, and multimodal groups.

**Figure 10:**
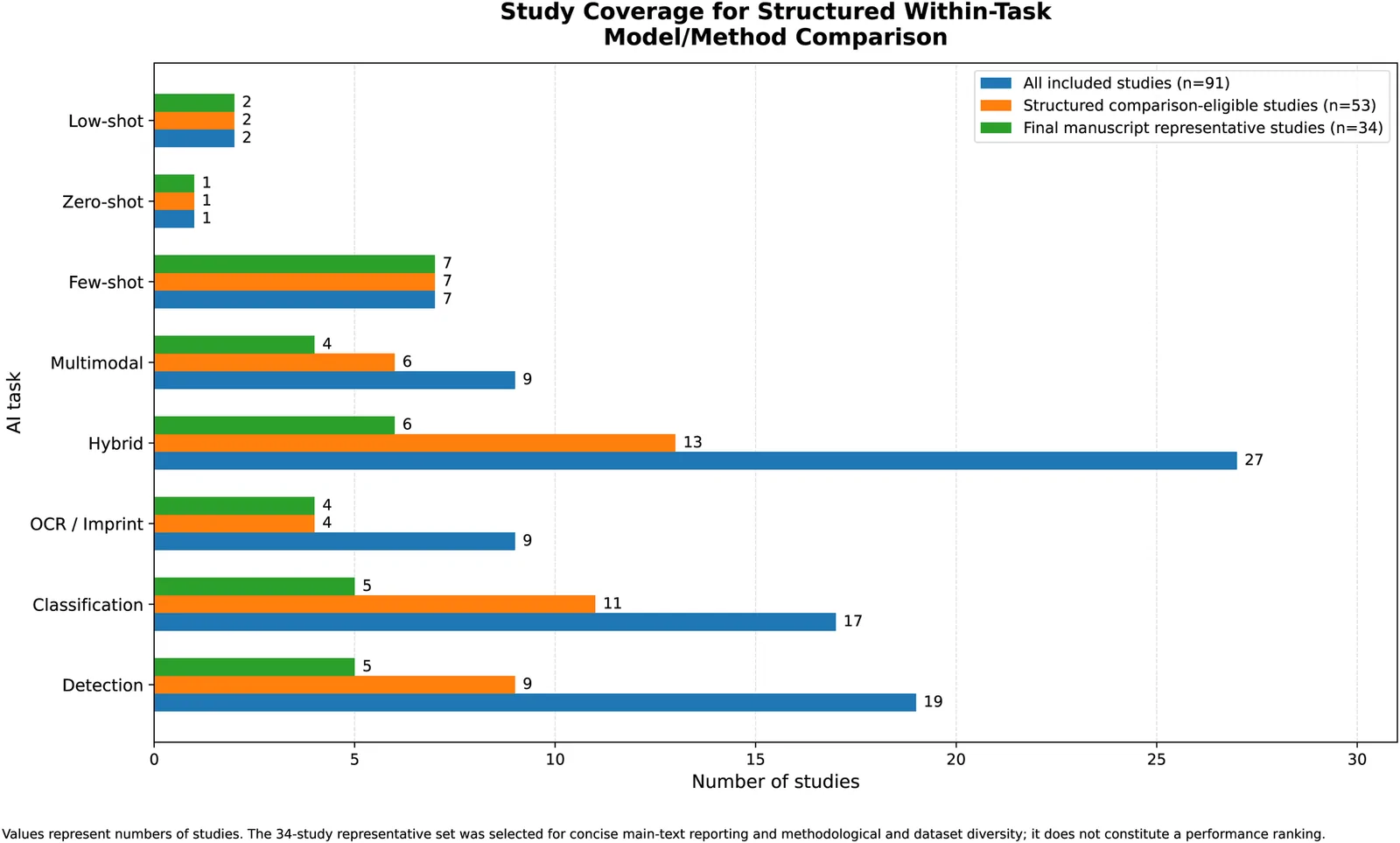
Study coverage for structured within-task model/method comparison across the 91 included medication-recognition studies. Values show the total included evidence base, the structured comparison-eligible studies, and the representative studies presented in the main manuscript tables.

Overall, the reported performance measures were interpreted within their relevant task, dataset, and evaluation context, rather than pooled into a common performance estimate or used to establish universal model rankings.

### 6.2 Evaluation metrics

The evaluation metrics used in the studies identified in this review differed based on the type of AI tasks implemented, which ranged from detection, classification, OCR/imprint recognition, and retrieval to few-shot or class-incremental recognition, and are summarized in Table 7. Commonly used metrics were accuracy, precision, recall, F1-score, and mean Average Precision (mAP). However, recognition of medications by accuracy alone might not be a robust measure of performance when multiple drug classes are present in a dataset with varying rates of occurrence or when there are varying consequences to a false positive vs. a false negative.

**Table 7:** Common evaluation metrics reported in medication recognition studies.

Metric Category	Reported Metrics	Typical Application
Detection	Precision, Recall, F1-score, IoU, mAP@0.5, mAP@0.5:0.95	Pill Detection
Classification	Accuracy, Precision, Recall, F1-score, Sensitivity, Specificity	Pill Classification
OCR / Imprint	CER, Precision, Recall, F1-score	Imprint Recognition
Retrieval	Top-1 Accuracy, Top-5 Accuracy, mAP	Pill Retrieval / Recognition
Few-shot / FSCIL	Accuracy, Top-1 Accuracy, Top-5 Accuracy, Average Accuracy (AA), Performance Drop (PD)	Few-shot and Zero-shot Recognition

In object detection, precision, recall, F1 score, Intersection over Union (IoU), mAP@0.5 and mAP@0.5:0.95 are widely used to assess performance in localization and recognition tasks. Studies that were done with the aim of classification mostly used accuracy, precision, recall, F1 score, sensitivity, and specificity when these statistics were available. For text recognition, two groups of methods employed OCR and imprint-recognition methods and reported the Character Error Rate (CER); retrieval-based and few-shot recognition studies reported the Top-1 accuracy, Top-5 accuracy, and mean Average Precision. Few-shot class-incremental studies also used Average Accuracy (AA) and Performance Drop (PD) to measure the recognition performance and the loss in performance from one incremental learning session to the next.

Overall, the literature examined demonstrates that none of the reviewed evaluation metrics can fully capture the recognition performance of medication across all AI methods. Complementary metrics are thus required to measure the localization accuracy, recognition performance, retrieval effectiveness, text-recognition quality, and performance as less or incremental training data are available. This task-based framework supports contextual comparison of AI-driven medication recognition systems across studies while accounting for differences in tasks, datasets, evaluation protocols, and validation conditions.

### 6.3 Structured within-task model and method comparison

#### 6.3.1 Detection-based methods

The detection studies reported performance under substantially different dataset sizes, medication-category counts, image-generation procedures, and validation settings, making direct numerical comparison appropriate only within comparable evaluation conditions. Table 8 therefore summarizes representative studies in terms of their experimental datasets, reported performance measures, and evaluation context rather than ranking the underlying detection architectures.

**Table 8:** Comparison of included detection-based medication recognition studies.

Author(s), Year	Model / Method	Dataset Type	Dataset Size	Performance Metrics
Dang et al. (2024) (27)	Customized YOLOv8	Public benchmark	8,693 pill photographs; 32 pill categories	mAP: 99.5%; precision: 98.1%; recall: 98.8%.
Ebron et al. (2024) (31)	YOLOv5 with data augmentation	Custom	1,500 images per front/back model; 70% training and 30% validation	mAP${}_{50-95}$: 0.848 for the front model and 0.869 for the back model.
Lee et al. (2025) (32)	YOLOv7 with VFI augmentation and automated labeling	Custom	480 original images; 61,440 augmented images; 40 pill types; 862 test images	Precision: 98.9%; recall: 100%; individual pill accuracy: 98.9%; image accuracy: 95.8%.
Ashraf et al. (2024) (8)	Microsoft Azure Custom Vision for pill detection and recognition	Custom, multicenter real-world	26,880 training images from 30 SODFs; 1,440 validation images; 960 device-dependency images; additional clinical images	Precision, recall, F1-score, mAP, and accuracy. Azure Custom Vision: 98.7% precision, 95.1% recall, 98.2% mAP; external clinical evaluation: 83.1% precision, 71.39% recall, 75.76% F1-score.
Tan et al. (2021) (39)	RetinaNet, SSD, and YOLOv3	Custom	5,131 original images; 261 drug varieties; 51,310 augmented images	Precision, recall, F1-score, mAP, and FPS. mAP: 82.89% (RetinaNet), 82.71% (SSD), and 80.69% (YOLOv3); FPS: 17, 32, and 51, respectively.

Dang et al. (27) evaluated a customized YOLOv8 model on a public benchmark containing 8,693 pill photographs across 32 pill categories and reported an mAP of 99.5%, with precision and recall of 98.1% and 98.8%, respectively. Ebron et al. (31) investigated YOLOv5 with data augmentation using separate front- and back-view models, reporting mAP${}_{50-95}$ values of 0.848 and 0.869, respectively. Lee et al. (32) combined YOLOv7 with VFI-based augmentation and automated labeling and reported precision of 98.9%, recall of 100%, individual pill accuracy of 98.9%, and image-level accuracy of 95.8% across 40 pill types.

Importantly, evaluation under more diverse or clinically oriented conditions provides additional information beyond benchmark performance. Ashraf et al. (8) evaluated Microsoft Azure Custom Vision using a multicenter real-world dataset and reported 98.7% precision, 95.1% recall, and 98.2% mAP in the reported model evaluation, while external clinical evaluation resulted in lower precision (83.1%), recall (71.39%), and F1-score (75.76%). This difference illustrates the importance of considering validation context when interpreting detection performance. Tan et al. (39) further demonstrated a computational-performance trade-off by comparing RetinaNet, SSD, and YOLOv3. Although the reported mAP values were 82.89%, 82.71%, and 80.69%, respectively, the corresponding frame rates were 17, 32, and 51 FPS, highlighting the relevance of inference efficiency alongside detection performance.

Overall, the detection studies demonstrate high reported localization performance across several experimental settings, but the results are not directly equivalent because the studies used different datasets, medication categories, image-generation procedures, evaluation metrics, and validation conditions. Therefore, the reported values should be interpreted as study-specific performance estimates rather than evidence of universal superiority of one detection architecture over another.

#### 6.3.2 Classification-based methods

The classification studies reported medication-identification performance across datasets that differed considerably in image volume, number of medication classes, and evaluation measures. The values presented in Table 9 should therefore be interpreted in relation to the corresponding experimental conditions rather than as a direct ranking of classification models.

**Table 9:** Comparison of included classification-based medication recognition studies.

Author(s), Year	Model / Method	Dataset Type	Dataset Size	Performance Metrics
Laohakijvitoon et al. (2024) (43)	VGG16, Inception-V3, and ResNet50-V2 with transfer learning	Custom	6,240 images; 26 drugs	Accuracy: 88.46%; precision: 98.46%; recall: 82.05%; F1-score: 89.51%.
Ahammed et al. (2024) (47)	ResNet50, Inception-V3, and MobileNet stacking ensemble	Public (Kaggle)	10,000 images; 10 pill classes	Accuracy: 98.80%; precision: 98.81%; recall: 98.80%; F1-score: 98.80%.
Yoon et al. (2025) (51)	ResNet50-based CNN	Custom	4,080 images; 102 pill types	Accuracy: 88.80%; macro-F1: 88.55%; ROC-AUC: 0.948.
Ting et al. (2020) (52)	YOLOv2-based deep-learning drug identification	Custom	250 blister-packaged drug types; 36,000 images reported	Front F1-score: 93.72%; back F1-score: 95.99%.
Wong et al. (2017) (54)	ImageNet-pretrained Deep Convolutional Network	Custom	5,284 images; 400 tablet/capsule classes	Top-1 accuracy: 95.35±0.22%; Top-5: 98.75±0.35%; Top-10: 99.55±0.21%.

Laohakijvitoon and Nilwong (43) evaluated VGG16, Inception-V3, and ResNet50-V2 using transfer learning on 6,240 images representing 26 drugs. The study reported an accuracy of 88.46%, together with precision of 98.46%, recall of 82.05%, and an F1-score of 89.51%. The difference between precision and recall indicates that the reported performance was not uniform across evaluation measures, emphasizing the importance of considering multiple metrics when assessing medication-recognition systems.

Ahammed et al. (47) used a stacking ensemble combining ResNet50, Inception-V3, and MobileNet on a public Kaggle dataset containing 10,000 images across 10 pill classes. The reported accuracy, precision, recall, and F1-score were all approximately 98.8%. In contrast, Yoon et al. (51) evaluated a ResNet50-based CNN using 4,080 images covering 102 pill types and reported 88.80% accuracy, 88.55% macro-F1, and a ROC-AUC of 0.948. The substantially larger class count in the latter study illustrates why performance values should be interpreted together with the classification scope rather than considered independently.

Ting et al. (52) investigated deep-learning-based identification of blister-packaged drugs across 250 drug types and reported separate front- and back-view F1-scores of 93.72% and 95.99%, respectively. Wong et al. (54) evaluated an ImageNet-pretrained deep convolutional network on 5,284 images covering 400 tablet/capsule classes, reporting top-1, top-5, and top-10 accuracies of 95.35%, 98.75%, and 99.55%, respectively. Overall, these studies demonstrate that reported classification performance is closely linked to class count, dataset composition, image acquisition, and evaluation protocol. The reported results consequently provide study-specific evidence rather than a basis for declaring one classification architecture universally superior. Comparisons are most meaningful when datasets, medication categories, metrics, and evaluation procedures are sufficiently aligned.

#### 6.3.3 OCR and imprint recognition methods

The studies summarized in Table 10 evaluated medication recognition from textual information such as printed imprints, with substantial variation in image sources, dataset scale, recognition pipelines, and evaluation protocols. The reported results therefore need to be interpreted according to the specific recognition stage and evaluation setting rather than treated as directly comparable performance estimates.

**Table 10:** Comparison of representative OCR and imprint-recognition approaches for medication recognition.

Author(s), Year	Model / Method	Dataset Type	Dataset Size	Key Performance
Jo et al. (2026) (55)	GO-PILL: TextSnake with MMOCR/ABINet for imprint localization and rectification	Public (NLM)	2,032 pill images	Precision: 81.93%; Recall: 81.73%; F1-score: 81.83%; Top-1/Top-5/Top-10: 46.63%/68.80%/76.52%.
Maitrichit et al. (2020) (61)	Tesseract OCR and Keras-OCR with word correction	Custom, hospital-based	34,000 images; 200 medicine types	Keras-OCR: 83% without and 92% with correction; Tesseract: 52% and 78%; combined CNN + Keras-OCR: 94.23%.
Heo et al. (2023) (14)	YOLOv5 imprint detection with RNN/GRU correction and ResNet-32	Public (MFDS and NLM)	24,404 samples	MFDS Top-1/Top-3: 85.65%/92.35%; NLM reference: 74.46%/88.70%; NLM consumer: 78.00%/89.10%.
Sony et al. (2025) (57)	YOLOv8 with EasyOCR	Custom	∼5,000 images; 50 medications	Precision, Recall, F1-score; F1: 0.93 (paracetamol), 0.88 (ibuprofen), 0.91 (aspirin).

Jo et al. (55) evaluated GO-PILL using TextSnake with MMOCR/ABINet for imprint localization and rectification on 2,032 publicly available pill images from the NLM. The study reported precision of 81.93%, recall of 81.73%, and an F1-score of 81.83%, while Top-1, Top-5, and Top-10 recognition rates were 46.63%, 68.80%, and 76.52%, respectively. These results illustrate the distinction between text localization/recognition performance and final medication identification performance, as the latter may remain more challenging even when detection-related measures are comparatively higher.

Maitrichit and Hnoohom (61) evaluated Tesseract OCR and Keras-OCR using a hospital-based dataset of approximately 34,000 images covering 200 medicine types. Keras-OCR achieved 83% recognition without word correction and 92% with correction, while Tesseract improved from 52% to 78% following correction. A combined CNN and Keras-OCR pipeline achieved 94.23%, indicating the potential contribution of post-processing and integration with visual recognition components to overall recognition performance.

Heo et al. (14) used YOLOv5 for imprint detection together with RNN/GRU-based correction and ResNet-32, evaluating 24,404 samples from MFDS and NLM sources. Top-1/Top-3 recognition rates varied across MFDS and NLM reference and consumer subsets, ranging from 74.46% to 85.65% for Top-1 and from 88.70% to 92.35% for Top-3. Sony et al. (57) reported medication-specific F1-scores of 0.93 for paracetamol, 0.88 for ibuprofen, and 0.91 for aspirin using approximately 5,000 custom images.

Overall, the studies indicate that OCR/imprint recognition performance depends on the quality and source of textual information, correction procedures, medication coverage, and evaluation subset. Consequently, the reported values should be regarded as study- and protocol-specific evidence, rather than evidence that one OCR or imprint-recognition approach is universally superior.

#### 6.3.4 Hybrid-based methods

The hybrid studies in Table 11 combined multiple processing stages or complementary model components to address different aspects of medication recognition. Their reported results varied according to the combination of localization, classification, textual recognition, metadata retrieval, and image-processing components, as well as the size and composition of the evaluation datasets. Accordingly, the reported values are considered within their respective experimental settings rather than as evidence of a universal performance hierarchy.

**Table 11:** Comparison of representative hybrid-based studies.

Author(s), Year	Model / Method	Dataset Type	Dataset Size	Key Performance
Zeng et al. (2017) (20)	SVM localization + multi-CNNs with triplet loss and Knowledge Distillation	Public (NIH/NLM)	7,000 images; 1,000 pills	mAP: 0.375/0.829; Top-1: 52.7/73.7%; Top-5: 81.7/95.6% (one-/two-side)
Ou et al. (2020) (75)	EFPN-ResNet-50 + Inception-ResNet v2	Self-collected (DPID) custom	2.43 million images; 612 drug classes	Localization: 96.3%; Top-1/Top-3/Top-5: 82.1/92.4/94.7%
Sai Sachin et al. (2026) (3)	YOLOv12n + ConvNeXt-Tiny + metadata retrieval	Pillbox-derived custom	10,000 images; 200 classes	Detection mAP@50–95: 0.983; class/shape/color: 0.990/0.999/0.995
Chotivatunyu et al. (2020) (81)	Detection + classification + OCR	Custom	56,000 classification images; 21,000 detection images	mAP: 0.875; AP@0.5: 0.998; classification: 93.79%; OCR: 99.71%
Sai Sachin et al. (2026) (84)	YOLOv26n + MobileNetV4-ConvSmall + EasyOCR	Pillbox-derived custom	20,000 images; 400 classes	Detection mAP@50: 0.989; classification accuracy: 99.60%; Top-1/Top-5: 89.83/99.49%
Liu et al. (2026) (85)	YOLOv12 + Faster R-CNN + SAM	Kaggle + curated dataset	152 public + 60 curated images	MAE: 0.167; detection accuracy: 93.3%; adherence accuracy: 91.7%

Zeng et al. (20) combined SVM-based localization with multiple CNNs incorporating triplet loss and knowledge distillation. Using 7,000 images involving 1,000 pills, the study reported mAP values of 0.375 and 0.829 for one- and two-side evaluation, respectively, with corresponding Top-1 accuracies of 52.7% and 73.7%, and Top-5 accuracies of 81.7% and 95.6%. The difference between one- and two-sided evaluation demonstrates the influence of available visual information on recognition performance.

Ou et al. (75) evaluated EFPN-ResNet-50 with Inception-ResNet v2 on a self-collected DPID dataset containing approximately 2.43 million images across 612 drug classes. The study reported 96.3% localization performance and Top-1, Top-3, and Top-5 recognition rates of 82.1%, 92.4%, and 94.7%, respectively. The substantially larger image collection and class coverage provide a different evaluation context from studies using smaller custom datasets.

Sai Sachin and Karthikeyan (3) combined YOLOv12n, ConvNeXt-Tiny, and metadata retrieval using a Pillbox-derived dataset of 10,000 images across 200 classes. It reported detection mAP@50–95 of 0.983 and class, shape, and color recognition values of 0.990, 0.999, and 0.995, respectively. Same authors (84) combined YOLOv26n, MobileNetV4-ConvSmall, and EasyOCR using 20,000 images across 400 classes, reporting detection mAP@50 of 0.989, classification accuracy of 99.60%, and Top-1/Top-5 recognition rates of 89.83%/99.49%.

Chotivatunyu and Hnoohom (81) integrated detection, classification, and OCR using separate custom datasets and reported mAP of 0.875, AP@0.5 of 0.998, classification accuracy of 93.79%, and OCR performance of 99.71%. Liu et al. (85) combined YOLOv12, Faster R-CNN, and SAM and reported detection accuracy of 93.3% and adherence accuracy of 91.7%, together with an MAE of 0.167.

Overall, these studies illustrate the potential of integrating complementary recognition stages, but their heterogeneous datasets, task definitions, metrics, and evaluation procedures limit direct numerical comparison. The reported results should therefore be interpreted as study-specific evidence of hybrid system performance, with particular attention to which component and evaluation stage each metric represents.

#### 6.3.5 Multimodal-based methods

The multimodal studies summarized in Table 12 incorporated information from more than one representation or processing source, with evaluation results varying according to dataset composition, medication-class coverage, and the evaluation environment. Because these studies used different combinations of visual, textual, and contextual information and reported different performance measures, their numerical results should be interpreted within their respective experimental settings.

**Table 12:** Comparison of representative Multimodal-based studies.

Author(s), Year	Model / Method	Dataset Type	Dataset Size	Key Performance
Balahariraj et al. (2026) (87)	PGPNet-X	Custom	18,400 images; 1,120 classes; 162,000 annotated pills	mAP@0.5: 91.3%; mAP@0.5:0.95: 63.1%; Top-1 accuracy: 93.8%; OCR CER: 8.7%
Radli et al. (2024) (91)	Multi-stream metric learning with image and text embeddings	Custom	4,480 images; 112 pill classes	Top-1 accuracy: 93.56%; Top-5 accuracy: 99.37%
Nguyen Thi My et al. (2026) (13)	Adaptive Lightweight Attention (ALA)	CURE; NLM	CURE: 8,649 images, 196 classes; NLM: 425 classes, ∼7 images/class	CURE: 96.23% accuracy, 93.07% mAP; NLM: 60.00±2.31% accuracy, 48.45±1.28% mAP
Nguyen et al. (2023) (93)	PGPNet	VAIPE	9,426 images; 96 classes; 1,527 prescriptions	69.7% mAP; 94.4% AP50; 83.4% AP75; 90.0% APs; 66.4% APm; 70.1% APl

Balahariraj et al. (87) evaluated PGPNet-X using a custom dataset containing 18,400 images and 162,000 annotated pills across 1,120 classes. The reported results included mAP@0.5 of 91.3%, mAP@0.5:0.95 of 63.1%, Top-1 accuracy of 93.8%, and an OCR character error rate (CER) of 8.7%. The difference between the two mAP measures and the inclusion of OCR CER indicates that the system was evaluated across multiple aspects of medication recognition rather than through a single performance measure.

Rádli et al. (91) used a multi-stream metric-learning approach based on image and text embeddings with 4,480 images covering 112 pill classes. The reported Top-1 and Top-5 accuracies were 93.56% and 99.37%, respectively, demonstrating the contribution of considering multiple ranked recognition candidates in addition to the top prediction.

Nguyen Thi My et al. (13) evaluated an Adaptive Lightweight Attention approach on both CURE and NLM datasets. Performance differed substantially between the two evaluation sources: accuracy and mAP were 96.23% and 93.07% on CURE, compared with 60.00±2.31% and 48.45±1.28% on NLM. This difference provides an important indication of the effect of dataset and evaluation-domain variation on reported performance.

Nguyen et al. (93) evaluated PGPNet on the VAIPE dataset containing 9,426 images across 96 classes and 1,527 prescriptions. The study reported an overall mAP of 69.7%, with AP50 of 94.4%, AP75 of 83.4%, and AP values of 90.0%, 66.4%, and 70.1% for small, medium, and large objects, respectively.

Overall, the multimodal studies show that performance can vary considerably across datasets and evaluation conditions. In particular, the CURE-to-NLM difference reported by Nguyen Thi My et al. (13) highlights the importance of external dataset evaluation when interpreting recognition results. Thus, the reported values should be considered context-dependent performance estimates, rather than evidence of universal superiority of a particular multimodal approach.

#### 6.3.6 Few-shot, zero-shot and low-shot learning-based methods

The studies summarized in Table 13 evaluated medication recognition under limited-reference or limited-training conditions, with substantial differences in the learning protocols, datasets, class coverage, and performance measures. Consequently, the reported values are interpreted according to the specific evaluation setting rather than used to establish a general performance hierarchy.

**Table 13:** Comparison of representative few-shot, zero-shot, and low-shot learning-based medication recognition studies.

Author(s), Year	Model / Method	Dataset Type	Dataset Size	Key Performance
Ling et al. (2020) (94)	Multi-Stream CNN with W${}^{2}$-Net and triplet-loss metric learning	NIH + CURE	NIH: 7,000 images, 1,000 classes; CURE: 8,973 images, 196 classes	5-way 1-shot accuracy: 64.2% (NIH); 53.7% (CURE)
Cordeiro et al. (2025) (95)	SSL4PILL with self-supervised pretraining and ProtoNet	NLM PIR + CURE	Exact evaluation subset not reported	Accuracy: 83.72% (NLM PIR); 60.78% (CURE)
Radli et al. (2023) (96)	MS-SA EfficientNet-B0 with Siamese/triplet metric learning	CURE + OGYEI	CURE: 8,973 images, 196 classes; OGYEI: 3,154 images, 78 classes	Top-1/Top-5: 85.72%/92.96% (CURE); 95.33%/99.78% (OGYEI)
Radli et al. (2024) (97)	MSFN/DM-MSFN	CURE + OGYEIv2	CURE: 8,973 images, 196 classes; OGYEIv2: 4,480 images, 112 classes	CURE Top-1/Top-5: 89.02%/98.67%; 89.20%/99.54%; OGYEIv2: 99.14%/100%
Chee et al. (2024) (98)	ProtoNet with ImageNet-pretrained ResNet-101	CURE	8,973 images, 196 classes	5-way 1-shot accuracy: 89.65%
Zhang et al. (2024) (99)	DBC-FSCIL with ResNet18	FCPill + mCURE	FCPill: 60,000 images, 100 classes; mCURE: 1,873 images, 171 classes	Final accuracy: 89.59% (FCPill); 78.15% (mCURE)
Zhang et al. (2026) (100)	WP-FSCIL with ResNet18	FCPill + mCURE	FCPill: 60,000 images, 100 classes; mCURE: 1,873 images, 171 classes	Final accuracy: 90.40% (FCPill); 78.35% (mCURE)
Nguyen et al. (2024) (103)	Zero-PIMA with pill localization	VAIPEPP	2,156 photos; 1,527 prescriptions; 6,366 pill objects; 107 classes	Seen/Unseen mAP: 68.71%/65.63%
Wang et al. (2017) (101)	GoogLeNet/Inception ensemble	NIH/NLM PIR	1,000 classes; 2,000 reference; 5,000 consumer images	mAP: 0.328; Top-1/5/10/20: 22.86%/49.06%/59.92%/69.52%
Usuyama et al. (2020) (102)	ResNet152 + CBP with multi-head metric learning	ePillID	13,532 images; 8,184 appearance classes	GAP: 81.20%; GAP@1: 91.19%; MAP: 95.76%; MAP@1: 92.01%

Ling et al. (94) evaluated a multi-stream CNN incorporating W${}^{2}$-Net and triplet-loss metric learning using NIH and CURE datasets. Under a 5-way 1-shot setting, the reported accuracy was 64.2% on NIH and 53.7% on CURE. Cordeiro et al. (95) used SSL4PILL, combining self-supervised pretraining with ProtoNet, and reported accuracies of 83.72% on NLM PIR and 60.78% on CURE. The difference between datasets in both studies indicates that recognition under limited examples can be sensitive to the characteristics of the evaluation source.

Rádli et al. (96) evaluated MS-SA EfficientNet-B0 with Siamese/triplet metric learning on CURE and OGYEI, reporting Top-1/Top-5 accuracies of 85.72%/92.96% and 95.33%/99.78%, respectively. Rádli et al. (97) reported CURE Top-1/Top-5 values ranging from 89.02%/98.67% to 89.20%/99.54% across the reported approaches, while OGYEIv2 results were 99.14% Top-1 and 100% Top-5. Chee et al. (98) reported 89.65% accuracy under a 5-way 1-shot setting using ProtoNet with an ImageNet-pretrained ResNet-101 on CURE.

For incremental and unseen-class recognition, Zhang et al. (99, 100) evaluated DBC-FSCIL and WP-FSCIL, respectively, on FCPill and mCURE. Final accuracies were 89.59% and 78.15% for DBC-FSCIL, compared with 90.40% and 78.35% for WP-FSCIL. Nguyen et al. (103) evaluated Zero-PIMA on VAIPEPP and reported seen/unseen mAP values of 68.71% and 65.63%. These results directly illustrate the distinction between recognition of seen and unseen medication classes.

Wang et al. (101) reported mAP of 0.328 and Top-1/5/10/20 values of 22.86%, 49.06%, 59.92%, and 69.52%, respectively, using NIH/NLM PIR data. Usuyama et al. (102), using ePillID with 13,532 images across 8,184 appearance classes, reported GAP of 81.20%, GAP@1 of 91.19%, MAP of 95.76%, and MAP@1 of 92.01%.

Overall, these findings indicate that limited-example and unseen-class recognition remains highly dependent on the evaluation protocol, reference examples, dataset characteristics, and class composition. Results obtained under 5-way 1-shot, incremental-learning, or seen/unseen settings should therefore not be directly compared with conventional fully supervised classification results. The reported values provide evidence about performance under their respective limited-data scenarios rather than demonstrating universal superiority of any particular learning strategy.

### 6.4 Computational efficiency and deployment considerations

Computational efficiency and deployment feasibility varied across the reviewed medication-recognition studies, reflecting differences in model architecture, processing stages, hardware platforms, input requirements, and application scenarios. Importantly, inference speed and computational characteristics were not reported consistently across studies, limiting direct comparison of computational efficiency. Where reported, measures such as frames per second (FPS), inference time, and deployment platform provide additional context for interpreting the practical applicability of recognition systems.

Several detection and classification studies reported explicit inference-speed measurements. Tan et al. (39) evaluated RetinaNet, SSD, and YOLOv3 and reported frame rates of 17, 32, and 51 FPS, respectively, illustrating that computational throughput can differ among models evaluated within the same experimental setting. Yoon et al. (51) reported approximately 3.5 ms per image for a ResNet50-based classification system, corresponding to approximately 285 FPS, in an automated sorting scenario. Similarly, Ou et al. (75) reported localization and classification times of 65 ms and 26.7 ms per pill, respectively, demonstrating the contribution of individual processing stages to overall inference time.

Deployment-oriented studies further demonstrated the importance of hardware and optimization conditions. Sai Sachin and Karthikeyan (3) reported 23 FPS for detection and 65 FPS for classification using a YOLOv12n–ConvNeXt-Tiny framework in an edge/clinical system. Nguyen Thi My et al. (13) evaluated an ONNX-optimized multimodal framework and reported 3.30 FPS on edge hardware and 4.38 FPS on GPU-based execution. These results illustrate that reported computational performance is dependent not only on the model architecture but also on the deployment environment and optimization strategy.

More complex hybrid, OCR-based, and multimodal systems may involve additional processing stages, including text localization, OCR, metadata integration, feature fusion, or semantic processing. Such components can increase functional capability while also introducing additional computational requirements. However, the available studies do not provide sufficiently standardized measurements to quantify these differences across methodological groups.

Overall, deployment readiness should not be inferred from recognition accuracy alone. Inference latency, throughput, model size, hardware requirements, optimization procedures, and real-world or edge-device validation should be considered together. The limited and heterogeneous reporting of these characteristics indicates a need for standardized computational and deployment evaluation in future medication-recognition studies.

### 6.5 Strengths and key achievements

The 91 studies in this review cover significant methodological advances in the field of AI-based medication recognition, ranging from detection, classification, OCR, and imprint recognition, to hybrid pipelines, multimodal systems, few-shot, zero-shot, and low-shot learning approaches. This is because there is now a growing recognition that the problem of medication recognition is not just about image classification, and is a multi-stage, context-dependent problem. The evidence base also relies on public benchmarks, custom datasets, multicenter real-world data, and cross-dataset evaluation, providing a more comprehensive view of the type of experimental conditions in which medication-recognition systems have been studied.

One major advantage of the literature reviewed is the increasing use of complementary sources of information. Hybrid approaches are a combination of localization, visual classification, OCR, metadata, or retrieval, and multimodal approaches utilize visual, textual, prescription, and/or contextual information. These arrangements provide some practical ways for dealing with drugs that may look alike or lack visual details. This is furthered by OCR and imprint-recognition techniques, which allow for the recognition of something beyond its appearance by using the textual markings on the surface of medications.

The evidence also suggests a shift in focus towards recognition in limited data and changing class settings. Few-shot and low-shot methods are used to investigate the recognition problem where labeled examples are few or limited; zero-shot and class-incremental methods are used to investigate the recognition problem with new or unseen classes of medications. They are particularly applicable in settings where medications are recognized, such as a medication collection service where it is not always possible to capture and annotate all images for all medications. Nevertheless, the results reported in these studies are specific to the learning and assessment procedures used in each study.

Another important change has been towards more practical, deployment-oriented systems. There were a few reports that addressed inference speed, execution on edge devices, integration of mobile or automated systems, or optimized model implementations. Overall, these efforts suggest tangible advances toward practical applications, though there are some inconsistencies in the reporting of computational and deployment results in the larger evidence base.

The overall methodological diversity and the increasing focus on real-world recognition challenges, that is, visually similar medications, limited labeled data, multimodal information, unseen classes, and deployability, are the principal strengths of the current evidence. Concurrently, the methodological improvements reflected in these developments should be interpreted as collective progress in the field rather than as evidence that one recognition method is superior to another.

### 6.6 Limitations and failure scenarios

Although methodological advances were seen in the 91 studies included in the evidence base, some methodological limitations and failure conditions were also identified. One of the largest of these was the significant heterogeneity of the datasets, type of medications, and conditions of image acquisition, evaluation protocols, and reported performance measures. The number and type of medication classes, image source, training and testing methods, and recognition tasks were often variable across the studies, making it difficult to compare the number of drugs across approaches. Therefore, the high performance achieved with a particular experimental setup should not be extrapolated to other setups.

Other restrictions included the scale and composition of the datasets. Some studies used custom or limited datasets, and some studies reported large image augmentations based on a small number of original images. Such augmentation can result in an apparent increase in the number of training instances without necessarily introducing equivalent diversity of independent occurrences of the medication or of training conditions. Variability across domains, as evidenced by differences in scores between the benchmark and external datasets, further highlighted the potential impact of domain variation. For instance, Nguyen Thi My et al. (13) noted a significantly different result on CURE and NLM, and Ashraf et al. (8) reported reduced precision, recall, and F1 score in the external clinical test scenario than those reported in the model test scenario. The results indicate that recognition performance in the controlled setting may not accurately represent recognition in the real-world.

Failure scenarios were also associated with similar-looking drugs, little text information, modification of viewing conditions, and novel drug classes. The imprints may be unclear, incomplete, or distorted in OCR methods, and appearance-based methods may be confused if the medication has a similar appearance, shape, or surface. Few-shot, zero-shot, and class-incremental learning require appropriate reference information, and their reported performance depends on the quality of the reference data and the evaluation protocol used.

An additional limitation was methodological reporting. Not all studies reported the same computational characteristics, external validation, inference conditions, size of the model, and deployment hardware. The process of annotation was also not consistently described, nor was there a consistent description of independent verification or inter-annotator agreement. Such discrepancies in reporting limit any attempt to evaluate the capability of the results being reproduced and ready for practical use.

Combined, the evidence indicates that medication recognition systems remain sensitive to the dataset, how the dataset was acquired, how the system was evaluated, and where it is deployed. The development of the future, therefore, needs to focus on multiple and self-collected databases, an open and transparent data-splitting process, external and real-world validation, standard evaluation protocols, detailed reporting of the annotations, and reproducible measures of computation and deployment.

### 6.7 Generalization, robustness and scalability

Generalization and robustness remain key factors in making medication recognition an AI system function from a controlled experimental setting to a wide variety of real-world settings. There was a large degree of variation in the composition of datasets, medication-class coverage, conditions under which images were acquired, and how the images were validated across the 91 studies included. These differences indicate that reported performance should be considered in the context of the diversity and independence of the evaluation data, and not as a general representation of performance across a larger number of settings in which medications are evaluated.

Another way to gain insight into generalization capability is through cross-dataset and external evaluations, as in the case of NLM, where accuracy and mAP dropped to 60.00±2.31% and 48.45±1.28%, respectively, highlighting the performance changes when the evaluation source changes. Likewise, Ashraf et al. (8) reported the results of the external clinical test and found that it achieved lower precision, recall, and F1 score compared to the internal clinical test. In total, these results highlight that results from a single benchmark or internal test set might not reflect robustness to variations in data source or acquisition conditions.

Diversity is also directly related to scalability in the context of datasets. The number of medication classes and images in the studies ranged from relatively few to hundreds or more classes and images. More data can be used to provide wider coverage of classes; however, nominal image counts could be inflated by augmentation or repeated image acquisition techniques. The number of independent images, devices, environments, and acquisition conditions represented should therefore be considered, in addition to the number of covered medication classes, when assessing scalability.

Medications that look alike, shifts in lighting or orientation, partial occlusion, imprinted blurriness or incompleteness, and previously unseen classes are particularly challenging to render robust. Few-shot, zero-shot, and class-incremental approaches provide means to support recognition beyond classes used for training, but they are dependent on the quality of reference information and the evaluation procedure employed.

Overall, the evidence indicates that generalization and scalability have not yet been shown to be consistent across medication-recognition research to date. Further study is recommended to focus on independently collected external datasets, cross-dataset evaluation, various imaging devices and working environments, clearly distinguishable training and test data, and robust testing standardization. To reduce the evidence base required for scalable deployment in clinical, mobile, and community settings, reporting of performance under multiple acquisition conditions and new classes of medications would bolster the evidence.

## 7 Research gaps and open challenges

### 7.1 Dataset limitations and data scarcity

The challenges lie in the availability and diversity of datasets, as well as in the limitations of current AI models. Data availability and diversity continue to be significant hurdles in the creation of effective AI-driven medication recognition systems. While the literature reviewed covers datasets of a few thousand images to large-scale collections, significant variance exists in the number of classes of medications, image source, acquisition conditions, and reference images represented. For instance, in the case of fine-grained recognition, the number of images used for evaluation by Wang et al. (101) was 5,000 consumer images, with 1,000 classes of medications, while Usuyama et al. (102) used 13,532 images over 8,184 appearance classes, which is the number of appearance classes of all the medications in his inventory. The appearance of these medications may not be evenly represented, nor may the imaging conditions be uniform in this data.

The few-shot recognition in a category-disjoint scenario was investigated in Ling et al. (94), and the ePillID benchmark (102) focused on fine-grained recognition with limited consumer-image examples for thousands of pill types. Variations in the evaluation sources also suggest that recognition performance may be sensitive to the dataset characteristics, as in Ling et al. (94), different 5-way 1-shot accuracy values are reported on NIH and CURE datasets (13) respectively.

In terms of previously unseen medication classes, Nguyen et al. (103) presented an approach for zero-shot pill/prescription matching, and Zhang et al. (99, 100) introduced few-shot class-incremental recognition approaches. The methods reduce reliance on large amounts of labelled data, but also highlight the need for developing recognition systems which continuously adjust to ever-growing medication inventories.

These constraints have implications for the reliability and scalability of medication-recognition applications that are deployed in a variety of environments, including heterogeneous healthcare settings and communities. From a digital-health informatics perspective, these limitations can threaten the reliability and scalability of medication-recognition applications deployed in a variety of environments, including heterogeneous healthcare settings and communities. Future datasets should therefore seek to increase medication coverage, include images generated by the consumer, include various imaging conditions, and systematically document annotations. Such resources would facilitate more representative benchmarking and facilitate scaling up of recognition systems that would allow the recognition of rare and newly introduced medicines without significant retraining.

### 7.2 Challenges in imprint recognition and rare medication identification

The identification of medication markings remains a challenge in medication recognition because the geometry, visibility, orientation, depth, and image quality of a medication marking vary. In the reviewed literature, 9 of the 91 papers were dedicated to the specific problem of OCR or imprint recognition, in which case geometry-aware preprocessing together with imprint localization and rectification, and ABINet-based OCR was used to recognize printed, debossed, curved, diagonal, and linear imprints, and the results were presented in a paper authored by Jo et al. (55), and they developed the system, GO-PILL. Despite this, only 46.63% of the top-1 recognition was achieved, which suggests that the problem of imprint localization or text recognition is not directly correlated with the success of end-to-end medication identification.

The reviewed evidence also highlights significant image source sensitivity and sensitivity to correction procedures, with the results of Maitrichit and Hnoohom (61) showing that Tesseract and Keras-OCR were evaluated on about 34,000 images taken in hospitals across 200 different medicine types. After word correction, the performance of Keras-OCR improved from 83% to 92%, and a pipeline of CNN and Keras-OCR improved to 94.23%. These results highlight the need for visual-visual and visual-textual recognition stage preprocessing, textual correction, and integration. However, there remain certain differences in datasets, imaging conditions, imprint characteristics, and evaluation protocols, which hinder direct comparisons of approaches.

Rare and previously unseen medications pose a related data-scarcity problem because conventional supervised recognition requires labeled examples for each medication category. Ling et al. (94) studied category-disjoint few-shot pill recognition using the RGB, texture, contour, and imprint features, providing one approach to recognition in the absence of labeled examples for each medication category. Few-shot, low-shot, zero-shot, and class-incremental approaches are thus also potential ways to aid with the recognition of new or underrepresented medications; how effective they are depends on reference information, class coverage, and test set design.

Future research should focus from a digital-health informatics perspective on a variety of imprint sets, realistic consumer-acquired images, limited coverage of rare medications, OCR performance under distorted marking, standardized end-to-end evaluation, and methods for safely and reliably embedding new classes of medications into recognition and medication-information workflows.

### 7.3 Real-world clinical validation and deployment issues

The validation of AI-driven medication recognition in real-world healthcare settings is still a significant challenge in moving the technology from experimental models to practical digital healthcare solutions. In the 91 studies included, there were significant differences in the composition of the datasets, medications included, image-acquisition environments, validation procedures, computational requirements, and deployment context. Therefore, it is not possible to extrapolate from controlled or self-packaged sample sets to clinical or community settings.

One of the more illustrative examples is found in Ashraf et al. (8) who used a multicenter real-world dataset to assess the performance of Microsoft Azure Custom Vision. The model evaluation scored 98.7% of precision, 95.1% of recall, and 98.2% of mean average precision, whereas external clinical evaluation resulted in significantly lower precision (83.1%), recall (71.39%), and F1-score (75.76%). Independent clinical validation of this recognition gap demonstrates the value in having an independent group and suggests that there is a meaningful variance in recognition reliability due to differences in domain, acquisition, and image characteristics in the real world.

Another gap is along the deployment feasibility. A number of studies have considered mobile, embedded, wearable, or edge-oriented recognition but did not consistently report computational characteristics of the model, including such aspects as frame rate, as mentioned by Tan et al. (39) in their work, which compared the performance of the RetinaNet, SSD, and YOLOv3, reporting significantly diverging frame rates despite roughly similar mAP numbers. When implementing digital health, the recognition accuracy will need to be considered alongside factors such as latency, throughput, model size, hardware requirements, energy usage and optimisation, rather than in isolation.

Workflow integration, usability, robustness to varying imaging situations, and safe handling of unreliable or incorrect predictions are additional issues to consider for clinical deployment. The evidence reviewed indicates that these factors are not widely reported on a standardised basis to limit the ability to assess deployment readiness and reproducibility.

Future studies should focus on prospective and multicenter validation, independent external datasets, diverse devices and acquisition environments, robust test standardization, reporting in terms of computation, and evaluation in realistic medication-identification workflows. Such evidence would bolster the foundations for the embedding of AI-enabled medication recognition capabilities in clinical, community, and patient-facing digital-health environments, but not be interpreted as a measure of algorithmic effectiveness as clinical effectiveness.

### 7.4 Explainability, trustworthiness, and user acceptance

Explainability, trustworthiness, and user acceptance are unexploited aspects of AI-powered medication recognition for digital health deployment. The reviewed literature highlights that the majority of studies focus on detection, classification, or recognition performance, and not so much on explaining model predictions, communicating uncertainty, or supporting transparent decision-making. This gap is especially significant if recognition outputs are to be used for informing medication verification or patient-facing medication-management processes.

Some of the studies included in this research did include contextual mechanisms that could aid in the interpretation or verification process, such as visual recognition, OCR, packaging information, prescription verification, multimodal fusion, and graph reasoning, with examples of how complementary information can help the verification process. Balahariraj et al. (87) and Nguyen et al. (93) provided an example of integrating these different types of information. The attention-based and uncertainty-aware components were also mentioned in Brindha et al. (80), indicating the direction of uncertainty communication. Despite this, none of these approaches constitute a standard method to account for recognition decisions, and there is little evidence on explanations of less common drugs or more complex pictures.

However, the trustworthiness of a system is not limited to its algorithms’ performance. The reviewed studies provide little evidence for the ability of clinicians to interpret the output of systems, understand uncertain predictions, and/or to understand the rationale behind medication-identification decisions. Therefore, only accuracy measures cannot be used to qualify an AI system as trustworthy and appropriate for medication recommendation. The study’s results are consistent with the overall conclusion of this review—that performance estimates must be interpreted with care and should not be considered a standalone measure of clinical effectiveness.

Likewise, user acceptance has not been sufficiently studied. The usability and patient acceptance, clinician trust, human–AI interaction, and workflow compatibility of mobile and patient-oriented systems are under-researched and have clear potential to be usable, but systematic assessment of these areas needs further research. Clinically meaningful interfaces, calibrated uncertainty communication, human-in-the-loop verification, usability testing, and prospective studies should be evaluated in future studies for explainability. These assessments should look at how explanations support rather than just build confidence in appropriate ways. Transparent, explainable, and suitably conservative interaction mechanisms will play a key role in establishing safe and patient-centered digital-health workflows that incorporate medication recognition.

### 7.5 Privacy, security, and regulatory considerations

The privacy and security aspects of medication recognition via AI are still under-developed, with systems advancing from image-based to more comprehensive digital-health operations, and the regulatory framework is not yet fully established. The analysed evidence covers architectures that process the images of the medicines, prescription information, QR or bar code data, packaging information, etc., and the language-based inputs, which result in sensitive information flows that are subject to careful governance.

For instance, in Hsu and Su (89) the identification of the pills is based on YOLOv4 and the verification of the prescription with QR is implemented on an embedded platform, and in Chen et al. (92) the image classification is integrated with the prescription information provided by the barcode/QR code to facilitate the verification of the automated dispensing. These architectures show increasing integration of recognition outputs with medical information, as in Nguyen Thi My et al. (13) where visual, imprint and OCR information is used in edge device recognition; no specific privacy or security protections are defined in the cited work.

The introduction of multimodal systems adds extra governance considerations, as seen in Balahariraj et al. (87), which also incorporates visual recognition, OCR, packaging and logo information, prescription verification, multimodal fusion and graph reasoning, while Menaha et al. (90) fuses medication images with textual information in a generative-AI framework. Such architectures also enhance the quantity of information flows and emphasize the importance of managing access to data, data storage security, and data provenance, as well as data uncertainty and faulty outputs.

Another area of shortage relates to regulatory and safety preparedness. The reviewed studies show a wide range of variations in reporting the deployment conditions, external validation, annotation procedures, and uncertainty-related components. Brindha et al. (80) introduced uncertainty-aware recognition, but this is still not enough to enable the development of standardized safety mechanisms or regulatory readiness. Importantly, the evidence base fails to provide a consistent evaluation of privacy protection, cybersecurity, or regulatory compliance or the governance requirements more generally.

Future research should therefore consider the principles of privacy by design, secure data handling, explicit consent, and data-governance procedures if applicable, auditability, uncertainty communication, human oversight, and documented data-governance and risk-management procedures. Technical validation needs to be accompanied by regulatory evaluation in the context of intended healthcare environments where systems are intended for clinical, dispensing, or patient-facing use, and should include an assessment of medication recognition, as this is not only a question of image-recognition algorithm but also part of a safe and accountable digital-health service.

### 7.6 Trade-offs between accuracy, efficiency, and scalability

The applicability of AI-driven medication recognition in real-world digital-health systems still faces the difficulties of balancing recognition accuracy, computational efficiency, and scalability. The reviewed studies reveal that enhanced recognition accuracy is frequently achieved by increasing the number of processing steps, the number of models, the number of modalities of the input, or the size of the reference information, which may increase the computational load. The trade-off becomes critical when recognition is part of the patient-facing, clinical, or community medication-management processes.

This relationship was very well demonstrated in an experimental framework shared by Tan et al. (39). RetinaNet, SSD, and YOLOv3 obtained the mAP of 82.89%, 82.71%, and 80.69%, respectively, and the performance of their frame rate was reported as 17, 32, and 51 FPS. This shows how important it is to consider recognition performance in the context of inference throughput, and not as a standalone measure of deployability. Likewise, Sai Sachin and Karthikeyan (3) achieved 23 FPS for detection and 65 FPS for classification with an edge/clinical system, while Nguyen Thi My et al. (13) attained 3.30 FPS on edge hardware and 4.38 FPS on GPU execution, which illustrates the impact of the deployment environment and optimization decisions.

As classes of medications, image acquisition conditions, and user needs grow, scalability adds additional difficulties. As illustrated in Zhang et al. (99, 100), few-shot and class-incremental approaches provide ways of adding a new category of medicines with a small amount of labeled data. Despite this, there is a strong link between their reported performance and the evaluation procedures or reference information they were based on.

Future studies should include accuracy, latency, throughput, model size, memory requirements, energy consumption, hardware architecture, and optimization procedure. Performance should be assessed with standardized benchmarks in the context of medication class expansion, across a variety of devices and under realistic acquisition conditions.This type of integration would enable digital-health developers to determine accurate, efficient operating points and provide scalable medication-recognition systems without assuming that higher accuracy necessarily corresponds to better deployability.

## 8 Medication recognition in digital health and healthcare applications

### 8.1 Adverse drug event prevention and medication safety

Medication safety is a digital-health context in which AI-based medication recognition may provide an enabling function for medication identification and verification. The 91 included studies demonstrate a progression from image-based detection and classification toward hybrid and multimodal systems that combine visual characteristics with imprint, prescription, metadata, or retrieval information. These developments are relevant to medication-safety workflows because identification can provide an additional verification layer before medication-related actions are undertaken, although the reviewed evidence does not establish reductions in adverse drug events or medication errors.

Several included studies illustrate this potential integration. Sai Sachin and Karthikeyan (3, 84) combined pill detection, multi-attribute recognition, and medication metadata retrieval, demonstrating how recognition outputs can be linked with additional medication information within an integrated workflow. Similarly, multimodal approaches incorporated prescription or barcode/QR information alongside visual recognition, providing mechanisms for verification rather than relying exclusively on pill appearance. Such architectures are particularly relevant to digital-health informatics because they can connect computer-vision outputs with structured medication information and downstream verification processes.

The evidence also indicates that safety-oriented interpretation requires attention to validation context. Ashraf et al. (8) reported high performance during model evaluation but substantially lower precision, recall, and F1-score during external clinical evaluation. This difference highlights that recognition performance obtained under controlled or development conditions may not directly translate to clinical reliability. Consequently, high accuracy should not be interpreted as evidence of medication-safety effectiveness.

From a digital-health perspective, AI-based medication recognition may therefore support medication verification, dispensing workflows, and patient-centred medication-management applications when integrated with reliable information resources and human oversight. Future evaluations should examine false-positive and false-negative recognition, uncertainty handling, external validation, workflow compatibility, usability, and safety outcomes. Such evidence would help determine whether recognition systems can function reliably as components of medication-safety infrastructures rather than merely as standalone computer-vision models.

### 8.2 Pharmacovigilance and healthcare monitoring

Pharmacovigilance and healthcare monitoring represent digital-health contexts in which medication recognition may provide a verification function. Within the 91 included studies, however, most investigations evaluated medication detection, classification, imprint recognition, or identification rather than pharmacovigilance outcomes. Therefore, the evidence supports a potential enabling role for recognition within monitoring workflows, but does not establish direct improvements in adverse-event detection, reporting, or medication safety.

Several studies illustrate how recognition can be connected with contextual medication information. Balahariraj et al. (87) developed PGPNet, integrating visual recognition with OCR, packaging and logo information, prescription verification, multimodal fusion, and graph reasoning. This architecture demonstrates how medication images can be linked with prescription-related and contextual information, which may support medication monitoring and verification. Similarly, Sai Sachin and Karthikeyan (3, 84) combined medication detection, multi-attribute recognition, and medication metadata retrieval, providing a pathway from visual identification to structured medication information. These approaches are relevant to digital-health informatics because recognition outputs can potentially be incorporated into longitudinal medication records or monitoring interfaces when supporting infrastructure is available.

Monitoring-oriented applications were also represented by studies addressing adherence, smart-pill detection, and application-oriented recognition (15, 17, 81). Such systems indicate that medication recognition can contribute to tracking or verification processes, but recognition of a medication image should not be equated with confirmed medication ingestion, adherence, or adverse-event occurrence.

The evidence further emphasizes the importance of validation before healthcare monitoring deployment. Differences across datasets and evaluation domains can substantially affect recognition performance, as demonstrated by Nguyen Thi My et al. (13). Consequently, future digital-health monitoring studies should evaluate temporal consistency, recognition uncertainty, external validation, data integration, false-alert burden, and human oversight. Prospective evaluation within monitoring workflows is needed to determine whether AI-based medication recognition can contribute to pharmacovigilance-related and healthcare-monitoring functions without overstating its clinical effectiveness.

### 8.3 Clinical decision support systems

Clinical decision support systems (CDSS) represent a digital-health application of AI-based medication recognition, particularly when recognition outputs are linked with medication information, prescription context, or verification mechanisms. However, the 91 included studies primarily evaluated recognition tasks rather than clinical outcomes. Accordingly, the evidence supports an enabling role for medication recognition within CDSS workflows, but does not demonstrate improved clinical outcomes.

Several studies illustrate mechanisms supporting such integration. Balahariraj et al. (87) developed PGPNet-X by combining visual recognition, OCR, packaging and logo information, prescription verification, multimodal fusion, and graph reasoning. This architecture connects medication identification with contextual information, providing an interface between recognition and information-supported processes. Sai Sachin and Karthikeyan (3, 84) similarly combined medication detection, multi-attribute classification, and medication metadata retrieval, demonstrating how recognized medication characteristics can be connected with medication information. Hsu and Su (89) integrated pill identification with QR-based prescription confirmation, while Chen et al. (92) incorporated image classification with barcode/QR prescription information for automated dispensing verification.

From a digital-health informatics perspective, these architectures may provide an input layer for systems that retrieve medication information, verify prescriptions, or present information to healthcare professionals or users. Nevertheless, medication recognition alone does not constitute clinical decision support. Functions such as interaction checking, contraindication assessment, dose evaluation, or treatment recommendation require validated clinical knowledge, patient-specific information, and decision-support logic that were not consistently evaluated in the included studies.

Future research should investigate how recognition outputs can be safely integrated with electronic medication records, prescription data, knowledge resources, and human-in-the-loop decision processes. Evaluation should include recognition uncertainty, false-positive and false-negative consequences, interoperability, usability, external validation, and prospective workflow assessment. Such studies would clarify whether AI-based medication recognition can provide reliable inputs to CDSS while maintaining clinical oversight and avoiding interpretation of recognition performance as evidence of clinical effectiveness.

### 8.4 Patient-centered mobile and assistive health applications

Patient-centered mobile and assistive applications are digital-health setting examples for AI-based medication recognition, which are those involving a portable or user-facing interface for medication identification. Evidence of applications for visually impaired users, mobile medication identification, voice assistance, smartphone imaging, wearable devices, and unconstrained acquisition is included. The reviewed studies focus on recognition performance and not on patient-level outcomes or sustained usability and indicate potential for improvement for access to information about medications.

Medication recognition for visually impaired users was covered by classification-based systems for medication recognition and mobile application systems (24, 25); meanwhile, voice-assisted recognition was explored for visually impaired users (46). Pill classification with the mobile case in mind (49) and smartphone-based recognition of pills in varying imaging conditions (53) show adaptation to portable acquisition. This application space is extended towards assistive and community-oriented use with wearable smart-glasses-based recognition (36) and elderly-oriented medication recognition (4). The examples shown here demonstrate the potential of medication recognition as an interaction layer between image acquisition and medication information.

From a digital-health informatics viewpoint, mobile and assistive systems can include recognition and OCR, retrieval, metadata, or voice output to present medication-related information in ways that users can access. Hybrid systems with detection, classification, and OCR (81) provide examples of how recognition stages can be added to a medication-identification workflow. Lightweight and edge-oriented recognition, such as multimodal processing reported by Nguyen Thi My et al. (13), could assist in the case of resource-constrained devices.

But, more than just recognition accuracy, patient-centered deployment requires the ability to recognize and practice this skill. There are possible differences in image quality, lighting, burden of interaction, accessibility, uncertainty communication, privacy, and user acceptance that could influence utility. There is limited evidence supporting an evaluation of these factors. Future research could thus incorporate participatory usability assessment, different user groups, interface design for accessibility, test external by consumer-generated images, human-in-the-loop verification, and prospective evaluation of suitable reliance on recognition outputs. This kind of research could provide clarity on the potential of AI in patient-facing digital health services to be safe and effective.

### 8.5 IoT-enabled smart medication systems

IoT-enabled smart medication systems represent an emerging digital-health context in which AI-based medication recognition can interact with embedded devices, sensing components, prescription information, and medication-handling workflows. Within the 91 included studies, IoT was not treated as an independent retrieval requirement; therefore, these applications should be interpreted as contextual extensions of medication recognition rather than as evidence of established IoT-enabled clinical systems.

Several studies demonstrated recognition architectures that can operate within connected or embedded medication workflows. Hsu and Su (89) combined YOLOv4-based pill identification with QR-based prescription confirmation on an embedded system, illustrating how visual recognition can be linked with digitally encoded prescription information. Similarly, iDispensing (92) integrated deep-learning image classification with barcode/QR-based prescription information for automated dispensing verification. These architectures demonstrate the potential for connecting medication recognition with device-level verification and medication-handling processes.

Adherence-oriented and smart-medication applications were also represented. Pinto et al. (15) investigated computer-vision-based medication detection in an adherence-oriented context, while Dhayanithi et al. (17) addressed smart pill detection. Such studies indicate that recognition technologies may provide an input layer for connected medication-monitoring systems. However, detection or recognition of a medication should not be interpreted as direct evidence of medication ingestion, adherence, or improved health outcomes.

From a digital-health informatics perspective, lightweight and edge-oriented processing may be particularly relevant where recognition is performed close to the point of data acquisition. Nguyen Thi My et al. (13) combined visual attributes, imprint information, attention mechanisms, and OCR within an edge-oriented recognition framework. This direction may reduce dependence on continuous centralized processing, although practical deployment requires assessment of computational resources, connectivity, latency, privacy, and reliability.

Future research should evaluate interoperability between recognition models, sensors, embedded platforms, medication databases, and health-information systems. Prospective studies should additionally assess device reliability, data security, uncertainty handling, human oversight, usability, and performance under real-world conditions. Such evaluations are necessary to determine whether AI-based medication recognition can function as a reliable component of connected digital-health infrastructures.

## 9 Future research directions

### 9.1 Multimodal and context-aware medication recognition

Future research directions would be further development of multimodal medication recognition from image analysis to context-aware digital-health workflows. The studies included in the review were found to be using several types of information sources: images of medicines, imprints, prescription information, packaging, textual representations, and relational knowledge; nine of these used more than one source. PIKA (86) combined CNN-based pill features with a prescription-related knowledge graph and contextual attention to achieve pill image retrieval; PGPNet-X was created by Balahariraj et al. (87) by incorporating visual recognition, OCR, packaging and logo information, prescription verification, multimodal fusion, and graph reasoning. These architectures can enable context-based identification, but cannot be ranked directly since they involve different modalities and different evaluation contexts.

The image-text metric learning was performed in Rádli et al. (96) on 4,480 images in 112 classes of pills, while the image-text metric learning was performed, and it was found that there was large variation between the performance of the CURE and the NLM in Nguyen Thi My et al. (13). Results in this class suggest that the performance of multimodal systems should not be verified solely based on one set of test data, but rather on a variety of medication classes, acquisition settings, devices, and independent datasets.

Prescription confirmation and dispensing verification are examples of potentially useful relationships that could also be used in context-aware recognition, as described in Hsu and Su (89) and Chen et al. (92); lightweight multimodal processing is useful for an edge-oriented deployment in Nguyen Thi My et al. (13). Interoperability with medication databases, electronic medication records, prescription systems, and patient databases should also be tested, though not necessarily as components of contextual recognition; contextual recognition doesn’t necessarily mean clinical decision support.

Finally, uncertainty, conflicting evidence, privacy, transparency, computational efficiency, and human oversight should be taken into consideration by multimodal systems. Evaluation should include modality-specific errors, the impact of false positives and false negatives, external validation, latency and resource requirements. Future research on digital health should include a multimodal context to investigate the usability and safety of recognition vs. its actual clinical effectiveness in realistic scenarios, being careful not to break the distinction between recognition and actual clinical effectiveness.

### 9.2 Foundation models and generalized recognition systems

In addition to recognizing pills by fixed classifiers, Foundation models and generalized recognition systems have been proposed to address the need for more flexible systems that can combine visual, textual, prescription, and contextual information, with work such as that found in Menaha et al. (90) for multimodal drug identification using medication images, drug-name information, and a generative-AI framework using the Gemini model, and Nguyen et al. (103) for a framework that combines pill-image detection, prescription text and graph representations, and contrastive learning across both seen and unseen medication categories using the Zero-PIMA. These studies provide early and encouraging evidence; however, they have not yet proved to be accurate in every instance or found to be clinically effective.

The few-shot class-incremental recognition on FCPill and mCURE was also discussed by Zhang et al. (99) and Zhang et al. (100), respectively, demonstrating its relevance for expanding medication classes, which is another aspect that should be highlighted in future research. Nguyen et al. (103) also reported different mAP values between seen and unseen classes, which further highlights the importance of explicitly testing for generalized recognition under both conditions in future research.

Digital-health informatics should be a generalized system that links recognition with validated medication databases, prescription information, verified medication resources, electronic medication records, and patient-facing interfaces while maintaining human oversight. Evaluation should include whether foundation models can leverage context, identify uncertainty, reject predictions that are beyond their support, and distinguish between similar or unseen medications. Robustness should be evaluated over datasets, devices, imaging environments, classes of medications, and acquisition conditions that are not only within the intent of the application but also are independent of the other conditions.

Other factors to consider for deployment assessment are computational needs, latency, model size, privacy, data governance, explainability, and reproducibility. Future studies should examine generalized systems vs. task-specific approaches, using the same set of data and the same procedure, but without making assumptions about architecture. Such work might shed light on the potential scalability of the foundation-model recognition approach as inputs for medication verification and digital-health workflows, while still retaining the separation of generalized recognition with demonstrated clinical effectiveness and safety.

Segmentation-based pill recognition techniques for partially occluded, overlapping, and densely clustered pills should also be explored in future medication-recognition methodologies. These techniques could help to separate and identify individual drugs in more complex real-world imaging situations.

To conclude, the future direction of medication recognition research is poised to rely on foundation models, adaptability, and generalized learning methods that will provide scalable, data-efficient, and clinically applicable solutions in a rapidly changing digital-health landscape.

### 9.3 Explainable and trustworthy AI

Explainable and trustworthy AI ought to be a top research path for medication recognition as such, as recognition errors, uncertainty, and opaque predictions can impact the interpretation of the medication-identification outputs. Most of the reviewed evidence focuses on uncertainty-aware recognition (80), but explainability and calibrated confidence are not addressed across all of the evidence. Future systems should thus communicate information about the expected medication, a corresponding confidence level, uncertainty, and conditions in which it might be unreliable.

The opportunities associated with enhancing transparency are being explored by multimodal architectures that combine visual recognition with OCR, packaging information, logo information, prescription verification, multimodal fusion, and graph reasoning (87) and pill-image features with prescription-derived knowledge graphs and contextual attention (86). Digital-health informatics can benefit from architectures like this to include evidence-aware interfaces, as long as the source of each piece of information is explicitly stated and its reliability is known.

Future research should focus on interpretable recognition mechanisms that can recognize the relevant visual regions, imprint characteristics, textual evidence, or contextual relationships that lead to a given prediction. Explanations should be assessed by how well they maintain fidelity and are helpful, rather than as a means to automatically build trust. Confidence limits on images, abstention/referral procedures for images that lack confidence, and human-in-the-loop verification when confidence is low should also be built into systems. Such safeguards are most important for similar-looking drugs, partial imprinting, inconsistent drug procurement or manufacturing processes, and new drug classes.

Finally, trustworthiness should be evaluated using external, independent datasets and real digital-health workflows, as significant performance differences between the CURE and NLM datasets were found by Nguyen Thi My et al. (13). There is a need to include other elements in future evaluations, including robustness, uncertainty calibration, explanation quality, reproducibility, privacy considerations, and human factors in addition to traditional recognition measures. Prospective trials should be conducted on the use of transparent and uncertainty-aware interfaces to address appropriate reliance and safer verification without mixing up explainable recognition performance with demonstrated clinical effectiveness.

The conventional metrics of recognition systems (accuracy, precision, recall, F1-score, and mAP) need to be extended to include uncertainty-aware evaluation for future medication recognition systems. However, a highly accurate model could still make an incorrect prediction if the level of confidence is not well calibrated or if the image is not a medication that the model has never seen before for safety-critical identification of medicines. Evaluation based on calibration measures, such as expected calibration error (ECE), conformal risk-control approaches, and selective prediction, may provide further information about prediction reliability and enable systems to postpone uncertain predictions toward human verification. Such methods can enhance the security, transparency, and clinical validity of medication recognition with AI.

Overall, the future of medication recognition systems should strive to provide a balance between recognition performance, interpretability, uncertainty estimation, fairness, privacy, accessibility, and regulatory compliance. Creating these reliable and trustworthy platforms for AI could pave the way for safer human-AI interactions and bridge the gap between experimental systems and real-world, responsible digital-health solutions in the future.

### 9.4 Edge AI and resource-efficient deployment

Edge AI and resource-efficient deployment are essential for translating medication-recognition models into mobile, embedded, wearable, and other digital-health environments, where computational, connectivity, and latency constraints can limit cloud-based processing. The reviewed evidence shows growing attention to lightweight architectures and deployment-oriented evaluation, although computational reporting remains inconsistent and does not permit direct comparison across studies.

Tan et al. (39) compared RetinaNet, SSD, and YOLOv3, reporting 17, 32, and 51 FPS, respectively—results that illustrate the trade-off between recognition performance and throughput. Sai Sachin and Karthikeyan (3) reported 23 FPS for detection and 65 FPS for classification using a YOLOv12n–ConvNeXt-Tiny framework within an edge/clinical system. Nguyen Thi My et al. (13) evaluated an ONNX-optimized multimodal framework and reported 3.30 FPS on edge hardware vs. 4.38 FPS on GPU execution. Together, these findings suggest that deployment performance depends heavily on both architecture and hardware conditions.

For digital-health informatics, future systems should report inference latency, throughput, model size, memory and energy requirements, hardware configuration, optimization procedures, connectivity dependence, and performance under realistic acquisition conditions. Lightweight multimodal processing, as demonstrated by Nguyen Thi My et al. (13), may support local recognition while reducing reliance on centralized processing; resource efficiency, however, should never be pursued at the expense of recognition reliability, uncertainty handling, privacy, or human oversight.

Progress toward clinical evaluation and regulatory readiness is equally essential. Future development should draw on Good Machine Learning Practice, Software as a Medical Device (SaMD) principles, human-factors requirements such as ISO 62366, applicable IMDRF SaMD guidance, and emerging frameworks such as the EU AI Act. Clinical evaluation should incorporate multicenter validation, prospective assessment, usability testing, and established reporting frameworks such as CONSORT-AI and DECIDE-AI, along with post-market performance monitoring where applicable. Evaluation of this kind would help establish whether resource-efficient medication-recognition systems are sufficiently reliable, usable, and accountable for their intended digital-health deployment.

### 9.5 Federated learning and privacy-preserving healthcare systems

Federated learning and privacy-preserving computation represent promising future directions for medication-recognition systems operating within distributed digital-health environments. The reviewed evidence did not identify federated learning as an established methodological component of the included studies; its role should therefore be treated as a prospective research direction rather than a demonstrated capability of the current evidence base. This distinction matters when interpreting the potential for privacy-preserving deployment.

Several included studies already point toward architectures in which medication images are linked with prescription, barcode, QR, textual, or contextual information. Hsu and Su (89) integrated YOLOv4-based pill identification with QR-based prescription confirmation on an embedded platform, while iDispensing (92) combined deep-learning image classification with barcode/QR prescription information to support automated dispensing verification. Nguyen Thi My et al. (13) further demonstrated lightweight multimodal recognition using visual, imprint, and OCR information on edge devices. These architectures illustrate distributed, information-rich workflows in which data governance and controlled information exchange are likely to become increasingly important.

Future studies might examine federated training between hospitals, pharmacies, community sites, or patient-facing devices and allow for the development of models with heterogeneous data while keeping sensitive medication images localized. The communication overhead, client heterogeneity, non-independent data distributions, model convergence, privacy leakage, and robustness to corrupted or unreliable participants should be evaluated in such approaches, however. The mechanisms that ensure privacy must be considered in addition to recognition performance and not be assumed as part of distributed learning.

Future research is needed to design secure architectures for data governance that include the use of encryption, access control, auditing, data provenance, and, where applicable, a consent procedure. There were significant performance differences between the CURE and NLM datasets as shown by Nguyen Thi My et al. (13), indicating that domain variation cannot be assumed away and should be evaluated explicitly.

It is important to assess federated medication recognition using prospective, multicenter studies that include a comprehensive evaluation of privacy, reproducibility, robustness, usability, computational feasibility, and recognition reliability, not just accuracy.

### 9.6 Generative AI and autonomous healthcare systems

Generative artificial intelligence (AI) represents an emerging direction for extending medication recognition beyond image-level identification and toward interactive, context-aware digital-health applications. Within the 91 included studies, generative AI was not a dominant methodological category; however, Menaha et al. (90) demonstrated multimodal drug identification using medication images, drug-name information, and a Gemini-based generative-AI framework. This study offers an early indication that generative models can combine visual and textual medication information within a recognition workflow.

Future research could investigate generative AI systems that integrate medication recognition with structured medication databases, prescription information, retrieval mechanisms, and patient-facing interfaces. Such systems could potentially translate recognized medication information into understandable responses, support information retrieval, or provide context-sensitive assistance. Any generation of medication-related information, however, should remain grounded in verified sources, since generative outputs can contain inaccurate or unsupported information. Autonomous decision-making, accordingly, should not be inferred from the recognition capabilities demonstrated within the current evidence base.

The reviewed literature nonetheless provides several building blocks for this direction. Multimodal systems already combine medication images with prescription context, OCR, packaging information, textual representations, and graph-based knowledge, while hybrid frameworks connect recognition with medication-information retrieval and verification. These components could supply structured inputs for future generative systems, and Nguyen Thi My et al. (13) demonstrates the feasibility of lightweight multimodal processing on edge devices.

For digital-health informatics, future autonomous systems should therefore incorporate retrieval-grounded generation, provenance tracking, uncertainty communication, human oversight, access controls, and auditable interactions. Evaluation should extend beyond recognition accuracy to factual consistency, hallucination rates, response reliability, latency, privacy, usability, and performance across independent datasets and realistic healthcare workflows. Computational requirements deserve equal consideration, since multimodal and semantic processing can add considerable system complexity. Prospective studies are needed to determine whether generative AI can safely support medication-information workflows, without equating autonomous interaction with demonstrated clinical effectiveness.

## 10 Discussion

### 10.1 Principal findings and evolution of the evidence

The evidence base of 91 included studies points to a clear methodological evolution in AI-based medication recognition, moving from conventional image classification and object detection toward hybrid, multimodal, limited-data, and context-aware recognition systems. The taxonomy included 19 detection, 17 classification, 9 OCR/imprint, 27 hybrid, 9 multimodal, and 10 few-shot/low-shot/zero-shot studies, which show the progressive integration of complementary computational components. The categories should be viewed as methodological approaches, not as a hierarchy of performance, as the datasets, types of medications, acquisition conditions, and evaluation procedures varied significantly among the studies.

This evolution is also reflected in the development of visual recognition systems that combine textual, prescription, metadata, and retrieval data. Other authors such as (84) introduced the concept of integrating multimodal fusion with graph reasoning, and Nguyen et al. (86) introduced the concept of linking pill-image features to prescription-derived knowledge graphs. Others, such as Balahariraj et al. (87), combined visual recognition, OCR, packaging information, prescription verification, multimodal fusion, and graph reasoning, while Sai Sachin and Karthikeyan (3) combined detection and multi-attribute recognition with medication metadata retrieval. These advances provide valuable components for digital-health informatics processes, despite their fundamental recognition basis.

Another meaningful evolution is the limited-data approach investigated by Ling et al. (94) for the problem of few-shot pill recognition and by Nguyen et al. (103) for pill–prescription matching with unseen categories with Zero-PIMA. Such methods have been developed to tackle the practical problem of increasing the coverage of medication without large labeled datasets, but still require reference information and an evaluation design.

Importantly, the evidence further shows that there is a persistent translation gap, with significantly different performance found between the CURE and NLM datasets, and lower performance found when tested during external clinical evaluation vs the original clinical evaluation. These results highlight the importance of viewing the performance of a benchmark as only a first step towards generalizability and clinical effectiveness. In conclusion, the process of medication recognition is moving towards flexible components of digital health; however, further evidence should focus on external validation, standardized assessment, reproducibility, usability, uncertainty quantification, privacy, and the assessment of the real-world workflow.

The methodological assessment also showed high consistency between the two reviewers. Across the 728 paired D1–D8 domain-level assessments, observed agreement was 97.66%, with an aggregate Cohen’s κ of 0.959. This level of agreement supports consistent application of the predefined assessment framework, while the framework itself remains a manually developed study-level methodological assessment rather than a validated certainty-of-evidence instrument.

### 10.2 Evidence quality, generalizability, and validation

The quality of the evidence in the 91 studies included was quite variable, especially in terms of dataset construction, annotation, data splitting, evaluation methods, generalizability, reproducibility, and applicability to real-world settings. These characteristics were systematically examined using the D1–D8 assessment framework, which provided a structured view of study-level methodological concerns across the evidence base. There was a significant amount of variation in the number of datasets used and coverage of medication classes, with some studies using augmented images generated from relatively small original datasets. These characteristics may affect the strength of reported recognition results as well as the transferability of results.

Studies were also evaluated for their generalizability, with the work of Nguyen Thi My et al. (13) reporting 60.00±2.31% accuracy and 48.45±1.28% mAP in external clinical evaluation, but 96.23% accuracy and 93.07% mAP in their original evaluation domain, indicating significant domain sensitivity. Ashraf et al. (8) reported similar results, with lower precision, recall, and F1-scores in the external clinical evaluation, vs. the original evaluation domain. These findings reinforce the need to distinguish internal benchmark performance from evidence of generalizability.

There are also implications for digital-health informatics in terms of validation quality. Mobile, clinical, dispensing, or community acquisition workflows pose a different set of requirements for recognition systems, and these systems should be evaluated in various acquisition conditions, devices, medication classes, and environments. The evidence reviewed indicates that there was a lack of consistency in the documentation of external validation, computational reporting, annotation procedures, and deployment characteristics, limiting the possibility of reproducing the results and assessing readiness to implement.

The 91 included studies formed the complete evidence base for the review, whereas the 34 studies presented in the main comparative tables represented a purposively selected subset used to illustrate methodological, dataset, evaluation, and model-comparison diversity. The remaining studies were retained in the structured, conditional, or narrative synthesis and were not excluded from the overall evidence base. Accordingly, conclusions about methodological patterns and evidence gaps were informed by the complete set of 91 studies, while detailed comparative interpretation was limited to studies for which the reported evidence was sufficiently comparable.

This review was not aimed at estimating a single performance measure for the models and was not designed to rank the models universally. Comparative interpretation was only performed for studies where methodology and evaluation design were truly comparable. Future studies should emphasize the use of external data that is collected independently, the use of transparent patient or image separation where relevant, the use of protocols for evaluating the tools in a standardized manner, the inclusion of unseen datasets to test the tools, the use of prospective multicenter validation, and the reproducible reporting of computational and deployment conditions. Evidence must be evaluated in digital-health applications, with specific focus on usability, uncertainty handling, safety, interoperability, and workflow compatibility; and with the clear separation of evidence of technical recognition performance and evidence of clinical effectiveness.

### 10.3 Clinical translation and deployment readiness

To clinically translate and deploy AI-based medication recognition systems, there is a need for evidence to move beyond the recognition benchmark to reliable performance in real-world digital-health environments. The 91 studies included showed significant differences in the size of the datasets, medication coverage, the conditions under which these were collected, the validation processes, the computational demands, and the deployment environment. Therefore, for the purpose of reporting results, they should be interpreted in the context of specific studies and are not necessarily representative of clinical effectiveness or deployment readiness.

The use of external evaluation is especially illustrated in the example of Ashraf et al. (8). When the models were evaluated, Microsoft Azure Custom Vision had 98.7% precision, 95.1% recall, and 98.2% mAP while external clinical evaluation had significantly lower precision (83.1%), recall (71.39%), and F1-score (75.76%). This is a significant gap, indicating the need for independent clinical validation and that performance could vary substantially in clinical and acquisition environments.

Computational characteristics also affect the ability to deploy. Tan et al. (39) compared RetinaNet, SSD, and YOLOv3, reporting significantly different frame rates, yet generally similar mAP scores. Sai Sachin and Karthikeyan (3) reported 23 frames per second in detection and 65 in classification, while Nguyen Thi My et al. (13) reported 3.30 FPS for edge hardware and 4.38 FPS for GPUs. The results clearly indicate that, when evaluating the deployment feasibility, latency, throughput, model size, hardware, and optimization must be included alongside the recognition metrics.

In addition to workflow integration, usability, uncertainty communication, interoperability, privacy, human oversight, and reproducibility, clinical translation is a key consideration for digital-health informatics. Multicenter studies with different datasets, across various devices, should be performed, assessing the rate of recognition, the impact of false positive and false negative results, user interaction, and performance in realistic scenarios. Reporting frameworks and deployment-oriented evaluation should be clear about the difference between technical validation and clinical utility and safety. Such an approach would yield more robust evidence for use and/or assess the clinical value of medication-recognition systems in the clinical, community, dispensing, or patient-facing setting, while also avoiding an assumption of algorithmic accuracy as clinical value.

### 10.4 Medication recognition as a digital health component

Medication recognition with AI can be understood as a healthcare application of the digital-health spectrum, linking the acquisition of medical images with their recognition, information retrieval, verification, and the presentation to the user of healthcare-related applications. The conducted studies, listed in the 91, clearly indicate the evolution of OCR applications in the past years from simple methods of detection and classification towards hybrid and multimodal approaches that integrate contextual relationships, OCR, medication metadata, prescription information, and retrieval systems. This evolution implies medication recognition can be part of a larger digital-health workflows, not just an independent computer-vision problem.

Some of the included studies demonstrated this type of integration; for example, Sai Sachin and Karthikeyan (3) performed a combined study of medication detection, multi-attribute recognition, and medication metadata retrieval, from the visual recognition to structured medication information; Hsu and Su (89) combined image classification with QR-based prescription information for medication dispensing verification; and Chen et al. (92) merged image classification with barcode/QR prescription information for medication dispensing verification. These architectures demonstrate the connection between recognition outputs and complementary information sources and operational workflows in general.

Digital-health applications that are patient-centered point to this role. Studies included covered mobile medication identification, voice-based recognition, wearable recognition, and smartphone-based recognition as areas of potential application for accessible medication information services. However, the evidence that is available is mainly on recognition performance, with less attention paid to assessing sustained usability, patient outcomes, workflow integration, or appropriate use of the AI output.

This digital-health reading thus requires clarity. Medication recognition is not evidence of medication adherence, effectiveness of pharmacovigilance, clinical decision support, or patient outcomes. These functions rely on other validated information resources, clinical logic, human oversight, and prospective evaluation. Further research should focus on interoperability with electronic medication records, medication systems, prescription systems, and medication databases, as well as external validation, uncertainty communication, privacy, usability, and outcomes at the level of workflow. Such evaluation would be useful in elucidating the potential for AI-driven medication recognition to be a trusted and responsible tool in the realm of digital-health informatics without becoming a surrogate for the actual clinical effectiveness of the tool.

### 10.5 Implications for the next generation of medication recognition

Future medication recognition should go beyond single image classification accuracy and into intelligent, multi-modal, interoperable, and deployment-aware digital-health systems. The evidence base demonstrates methodological advancements from detection and classification to hybrid, multimodal, few-shot, zero-shot, and class-incremental approaches, which pave the way for future systems to handle increasingly varied medication-recognition scenarios. These developments should be interpreted as methodological progress rather than as evidence of proven clinical effectiveness.

Generalized recognition under changing medication classes and limited labeled data was a central topic of Nguyen et al. (103), and few-shot class-incremental recognition was explored by both Zhang et al. (99) and Zhang et al. (100). All of these methods provided the basis for systems that could be extended to accept new drugs without much manual labelling, but further work could be done with unseen classes, with standardised and independent datasets.

Medication images were also investigated along with drug-name information in the context of multimodal integration by Menaha et al. (90), and lightweight multimodal recognition by visual information, imprint, and OCR information was shown by Nguyen Thi My et al. (13) in the context of a generative-AI framework. Future systems should be integrated with validated medication databases and prescription information, electronic medication records, and patient-facing interfaces, with the retention of provenance, uncertainty communication, and human oversight.

The recognition ability vs. computation efficiency should also be considered in deployment-oriented development, as shown by different inference rates from different detection architectures in Tan et al. (39) and edge-oriented recognition performance in Sai Sachin and Karthikeyan (3, 84). Future evaluations should thus provide accuracy measures, in addition to latency, throughput, model size, hardware requirements, privacy, reproducibility, usability and external validation.

Medication recognition should therefore be assessed in prospective, multicenter, and standardized studies across realistic populations, devices, environments, and workflows, with evaluation covering recognition performance, computational efficiency, usability, safety, and clinical utility.

### 10.6 Limitations of the review

Several limitations should be considered when interpreting the findings of this systematic review. First, although the search was broadened to include Scopus, Web of Science, IEEE Xplore, and PubMed/MEDLINE and covered publications from 1 January 2016 to 4 September 2026, it was restricted to English-language publications and to studies meeting the predefined full-text eligibility criteria. As a result, relevant evidence published in other languages, unavailable as full text, or not indexed in the selected databases may not have been captured. The search strategy also prioritized medication-recognition and AI-method concepts over explicit safety, pharmacovigilance, or IoT terms, which strengthened technical retrieval but may have reduced retrieval of studies framed primarily around broader healthcare applications.

Second, methodological heterogeneity limited the scope for quantitative synthesis. The 91 included studies differed in medication coverage, dataset size and composition, imaging conditions, recognition tasks, evaluation metrics, validation strategies, and deployment settings. For example, Nguyen Thi My et al. (13) reported markedly different performance between the CURE and NLM datasets, while Ashraf et al. (8) observed lower performance during external clinical evaluation than during the original model evaluation. Findings such as these illustrate why reported metrics cannot be treated as directly comparable estimates across studies.

Third, the bespoke D1–D8 framework provided a structured study-level assessment of methodological concerns, but it was not a validated certainty-of-evidence instrument; it was designed to surface study-level methodological concerns rather than to generate an overall certainty-of-evidence rating. Given the substantial heterogeneity in study designs, recognition tasks, datasets, evaluation protocols, and reported outcomes, the synthesized evidence was interpreted qualitatively rather than through pooled quantitative estimates, with methodological limitations, validation strength, generalizability, and study-level concerns considered throughout the synthesis.

Finally, many included studies remained experimental or proof-of-concept in nature, with limited multicenter, longitudinal, or real-world validation. Privacy, security, interoperability, usability, and regulatory reporting were also addressed inconsistently across the evidence base. Rapid advances in multimodal, foundation, generative, and edge AI may further reshape this evidence landscape going forward. Conclusions should therefore be read as evidence of methodological progress rather than as established clinical effectiveness or deployment readiness.

## 11 Conclusion

This systematic review synthesized 91 primary studies examining AI-based medication recognition within a digital health informatics context. The evidence spanned detection, classification, OCR/imprint recognition, hybrid, multimodal, few-shot, low-shot, and zero-shot approaches, reflecting a methodological shift from isolated visual recognition toward systems combining visual, textual, prescription, metadata, and retrieval information. This progression provides a foundation for medication identification and verification across patient-facing, assistive, mobile, IoT-enabled, dispensing, and clinical decision-support workflows. However, technical recognition performance should not be equated with clinical effectiveness or medication-safety impact. Substantial heterogeneity across datasets, medication-class coverage, imaging conditions, evaluation protocols, validation strategies, and deployment contexts limits direct cross-study comparison and generalizability. External validation, cross-dataset evaluation, unseen-medication testing, prospective multicenter assessment, workflow integration, usability evaluation, and real-world clinical testing remain limited. The D1–D8 assessment identified methodological concerns related to dataset quality, annotation, data splitting and leakage, evaluation methodology, generalizability, reproducibility, real-world applicability, transparency, uncertainty, and safety. The high inter-rater agreement across the D1–D8 assessments supports consistent application of the predefined methodological criteria, while the framework should be interpreted as a structured study-level assessment rather than a validated certainty-of-evidence instrument. Reported benchmark performance should therefore be interpreted within its task, dataset, and evaluation context rather than as evidence of universal model superiority. Future research should prioritize diverse and standardized datasets, leakage control, independent external and multicenter validation, unseen-medication evaluation, reproducible reporting, explainable and uncertainty-aware AI, privacy-preserving computation, interoperability, and resource-efficient deployment. Evaluation should incorporate accuracy alongside latency, usability, safety, trust, workflow compatibility, and patient-centered outcomes. Because no formal publication-bias assessment, sensitivity analysis, or quantitative certainty-of-evidence assessment was performed, these findings represent a qualitative synthesis of a heterogeneous evidence base. Stronger prospective evidence is needed before clinical implementation.
