# Supplementary material for: Artificial intelligence for medication recognition and safety: a systematic review of detection, classification, OCR, and digital health applications
Source: Front Digit Health. 2026 Oct 15;8:1963607. doi: 10.3389/fdgth.2026.1963607 (PMC13717275; doi:10.3389/fdgth.2026.1963607)
Supplement: Supplementary file 1 [file Datasheet1.pdf]

# Data Extraction from Database:

**Block 1 — Medication/Pill Recognition**

(

"pill recognition"

OR "pill detection"

OR "pill classification"

OR "pill identification"

OR "pill image recognition"

OR "pill image classification"

OR "tablet recognition"

OR "tablet detection"

OR "tablet classification"

OR "tablet identification"

OR "tablet image recognition"

OR "capsule recognition"

OR "capsule detection"

OR "capsule classification"

OR "capsule identification"

OR "medication recognition"

OR "medication detection"

OR "medication classification"

OR "medication identification"

OR "medication image recognition"

OR "medicine recognition"

OR "medicine detection"

OR "medicine classification"

OR "medicine identification"

OR "medicine image recognition"

OR "drug recognition"

OR "drug detection"

OR "drug classification"

OR "drug identification"

OR "drug image recognition"

OR "drug image classification"

OR "imprint recognition"

OR "pill imprint recognition"

OR "medication imprint recognition"

OR "pill imprint"

OR "pill OCR"

OR "medication OCR"

)

**Block 2 — AI Methods**

(

"artificial intelligence"

OR "deep learning"

OR "computer vision"

OR "convolutional neural network"

OR "few-shot learning"

OR "zero-shot learning"

OR "multimodal learning"

OR "multimodal"

)

**Scopus**

TITLE-ABS-KEY(

"pill recognition"

OR "pill detection"

OR "pill classification"

OR "pill identification"

OR "pill image recognition"

OR "pill image classification"

OR "tablet recognition"

OR "tablet detection"

OR "tablet classification"

OR "tablet identification"

OR "tablet image recognition"

OR "capsule recognition"

OR "capsule detection"

OR "capsule classification"

OR "capsule identification"

OR "medication recognition"

OR "medication detection"

OR "medication classification"

OR "medication identification"

OR "medication image recognition"

OR "medicine recognition"

OR "medicine detection"

OR "medicine classification"

OR "medicine identification"

OR "medicine image recognition"

OR "drug recognition"

OR "drug detection"

OR "drug classification"

OR "drug identification"

OR "drug image recognition"

OR "drug image classification"

OR "imprint recognition"

OR "pill imprint recognition"

OR "medication imprint recognition"

OR "pill imprint"

OR "pill OCR"

OR "medication OCR"

)

AND

TITLE-ABS-KEY(

"artificial intelligence"

OR "deep learning"

OR "computer vision"

OR "convolutional neural network"

OR "few-shot learning"

OR "zero-shot learning"

OR "multimodal learning"

OR "multimodal"

)

**Web of Science**

TS=(

"pill recognition"

OR "pill detection"

OR "pill classification"

OR "pill identification"

OR "pill image recognition"

OR "pill image classification"

OR "tablet recognition"

OR "tablet detection"

OR "tablet classification"

OR "tablet identification"

OR "tablet image recognition"

OR "capsule recognition"

OR "capsule detection"

OR "capsule classification"

OR "capsule identification"

OR "medication recognition"

OR "medication detection"

OR "medication classification"

OR "medication identification"

OR "medication image recognition"

OR "medicine recognition"

OR "medicine detection"

OR "medicine classification"

OR "medicine identification"

OR "medicine image recognition"

OR "drug recognition"

OR "drug detection"

OR "drug classification"

OR "drug identification"

OR "drug image recognition"

OR "drug image classification"

OR "imprint recognition"

OR "pill imprint recognition"

OR "medication imprint recognition"

OR "pill imprint"

OR "pill OCR"

OR "medication OCR"

)

AND

TS=(

"artificial intelligence"

OR "deep learning"

OR "computer vision"

OR "convolutional neural network"

OR "few-shot learning"

OR "zero-shot learning"

OR "multimodal learning"

OR "multimodal"

)

**IEEE Xplore**

(

"pill recognition"

OR "pill detection"

OR "pill classification"

OR "pill identification"

OR "pill image recognition"

OR "pill image classification"

OR "tablet recognition"

OR "tablet detection"

OR "tablet classification"

OR "tablet identification"

OR "tablet image recognition"

OR "capsule recognition"

OR "capsule detection"

OR "capsule classification"

OR "capsule identification"

OR "medication recognition"

OR "medication detection"

```
 OR "medication classification"
 OR "medication identification"
 OR "medication image recognition"
 OR "medicine recognition"
 OR "medicine detection"
 OR "medicine classification"
 OR "medicine identification"
 OR "medicine image recognition"
 OR "drug recognition"
 OR "drug detection"
 OR "drug classification"
 OR "drug identification"
 OR "drug image recognition"
 OR "drug image classification"
 OR "imprint recognition"
 OR "pill imprint recognition"
 OR "medication imprint recognition"
 OR "pill imprint"
 OR "pill OCR"
 OR "medication OCR"
)
AND
(
 "artificial intelligence"
 OR "deep learning"
 OR "computer vision"
 OR "convolutional neural network"
 OR "few-shot learning"
 OR "zero-shot learning"
 OR "multimodal learning"
```

OR "multimodal"

)

**PubMed**

(

"pill recognition"[tiab]

OR "pill detection"[tiab]

OR "pill classification"[tiab]

OR "pill identification"[tiab]

OR "pill image recognition"[tiab]

OR "pill image classification"[tiab]

OR "tablet recognition"[tiab]

OR "tablet detection"[tiab]

OR "tablet classification"[tiab]

OR "tablet identification"[tiab]

OR "tablet image recognition"[tiab]

OR "capsule recognition"[tiab]

OR "capsule detection"[tiab]

OR "capsule classification"[tiab]

OR "capsule identification"[tiab]

OR "medication recognition"[tiab]

OR "medication detection"[tiab]

OR "medication classification"[tiab]

OR "medication identification"[tiab]

OR "medication image recognition"[tiab]

OR "medicine recognition"[tiab]

OR "medicine detection"[tiab]

OR "medicine classification"[tiab]

OR "medicine identification"[tiab]

```
  OR "medicine image recognition"[tiab]
  OR "drug recognition"[tiab]
  OR "drug detection"[tiab]
  OR "drug classification"[tiab]
  OR "drug identification"[tiab]
  OR "drug image recognition"[tiab]
  OR "drug image classification"[tiab]
  OR "imprint recognition"[tiab]
  OR "pill imprint recognition"[tiab]

  OR "medication imprint recognition"[tiab]
  OR "pill imprint"[tiab]
  OR "pill OCR"[tiab]
  OR "medication OCR"[tiab]
)
AND
(
  "artificial intelligence"[tiab]
  OR "deep learning"[tiab]
  OR "computer vision"[tiab]
  OR "convolutional neural network"[tiab]
  OR "few-shot learning"[tiab]
  OR "zero-shot learning"[tiab]
  OR "multimodal learning"[tiab]
  OR multimodal[tiab]
)
```
