# Supplementary material for: Artificial intelligence for medication recognition and safety: a systematic review of detection, classification, OCR, and digital health applications
Source: Front Digit Health. 2026 Oct 15;8:1963607. doi: 10.3389/fdgth.2026.1963607 (PMC13717275; doi:10.3389/fdgth.2026.1963607)
Supplement: Supplementary file 2 [file Datasheet9.pdf]

PRISMA 2020 CHECKLIST

PRISMA 2020 Checklist for the Systematic Review

"Artificial Intelligence for Medication Recognition and Safety: A Systematic Review of Detection, Classification, OCR, and Digital Health Applications"

Section / Topic	Item	PRISMA 2020 checklist item	Location in updated manuscript
TITLE	1	Identify the report as a systematic review.	p. 1 – Title identifies the work as a systematic review.
ABSTRACT	2	See the PRISMA 2020 for Abstracts checklist.	pp. 1–2 – Structured Abstract.
INTRODUCTION	3	Describe the rationale for the review in the context of existing knowledge.	pp. 2–5 – Sections 1.1–1.4; rationale, challenges and knowledge gaps.
INTRODUCTION	4	Provide an explicit statement of the objective(s) or question(s) the review addresses.	pp. 5–6 – Section 1.5; RQ1–RQ6.
METHODS	5	Specify the inclusion and exclusion criteria for the review and how studies were grouped for the syntheses.	pp. 10–12 – Section 2.4 and Table 3; synthesis grouping described in Section 2.9.
METHODS	6	Specify all databases, registers, websites, organisations, reference lists and other sources searched or consulted, and the date when each source was last searched or consulted.	pp. 9–10 – Section 2.3 and Table 2; Scopus, Web of Science, IEEE Xplore and PubMed/MEDLINE; search conducted 4 September 2026.
METHODS	7	Present the full search strategies for all databases, registers and websites, including filters and limits used.	pp. 9–10 – Section 2.3 and Tables 1–2; conceptual search blocks and database-specific fields are reported. Complete database-specific search strings are provided in Supplementary S1.
METHODS	8	Specify the methods used to decide whether a study met the inclusion criteria, including number of reviewers, independence	pp. 12–13 – Section 2.5; two reviewers independently screened title/abstract and full texts; disagreements were resolved by

Section / Topic	Item	PRISMA 2020 checklist item	Location in updated manuscript
		and disagreement resolution.	consensus.
METHODS	9	Specify the methods used to collect data from reports, including number of reviewers, independence and disagreement resolution, and automation tools if applicable.	p. 15 – Section 2.7; Reviewer 1 extracted study-level data and Reviewer 2 independently verified the extraction; discrepancies were resolved by consensus. Supplementary S2 contains the extraction data.
METHODS	10a	List and define all outcomes for which data were sought and state whether all compatible results were sought.	p. 15 – Section 2.7; performance metrics, validation, generalizability, deployment and other study-level outcomes were extracted where reported. No intervention-effect outcome was prespecified.
METHODS	10b	List and define all other variables for which data were sought and any assumptions made about missing or unclear information.	pp. 15–16 – Section 2.7; study, AI, dataset, evaluation, validation, deployment, OCR, IoT/edge, mobile and XAI variables were extracted where reported.
METHODS	11	Specify the methods used to assess risk of bias, including tool(s), number of reviewers, independence and disagreement resolution.	pp. 13–15 – Section 2.6; bespoke D1–D8 framework, independently applied by two reviewers to 91 studies. Across 728 paired domain-level assessments, 711 agreements and 17 disagreements yielded 97.66% observed agreement and aggregate κ=0.959; disagreements were resolved through consensus. Supplementary S3 contains assessments.
METHODS	12	Specify for each outcome the effect measure(s) used in the synthesis or presentation of results.	pp. 15–16, 29–40 – Task-specific performance metrics were retained in their original form because recognition tasks used heterogeneous measures; no common effect-size measure was used.

Section / Topic	Item	PRISMA 2020 checklist item	Location in updated manuscript
METHODS	13a	Describe the processes used to decide which studies were eligible for each synthesis.	pp. 10–12 and 19–20 – Eligibility, extraction, evidence mapping and qualitative cross-study synthesis; the 91-study evidence base and structured/conditional/narrative synthesis groups are described.
METHODS	13b	Describe methods used to prepare data for presentation or synthesis, such as handling missing summary statistics or data conversions.	pp. 15–20 – Structured extraction, standardisation and preservation of study-specific information; reported performance measures were not converted into a common metric.
METHODS	13c	Describe methods used to tabulate or visually display individual study results and syntheses.	pp. 20–21 and 29–40 – Evidence maps, comparative tables and Figures 4–8; supplementary evidence maps and study-level datasets.
METHODS	13d	Describe methods used to synthesise results and provide a rationale; if meta-analysis was performed, describe its statistical methods and software.	pp. 19–20 – Section 2.9; qualitative/descriptive cross-study synthesis and evidence mapping were used because of substantial heterogeneity; no meta-analysis was performed.
METHODS	13e	Explore possible causes of heterogeneity among study results and report analyses undertaken to investigate them.	pp. 19–20 and 63–64 – Heterogeneity was examined descriptively across tasks, datasets, metrics, validation, generalizability and deployment contexts; no formal subgroup/meta-regression analysis was performed.
METHODS	13f	Describe any sensitivity analyses conducted to assess robustness of the synthesized results.	p. 64 – The manuscript states that no formal sensitivity analysis was conducted because of the heterogeneous evidence base.
METHODS	14	Describe methods used to assess risk of bias due to missing results in a synthesis (reporting bias).	p. 64 – The manuscript transparently states that no formal publication/reporting-bias assessment was conducted.
METHODS	15	Describe methods used to	pp. 14–15 and 64 – No formal

Section / Topic	Item	PRISMA 2020 checklist item	Location in updated manuscript
		assess certainty (or confidence) in the body of evidence for an outcome.	certainty-of-evidence framework was applied; D1–D8 is a study-level methodological risk-of-bias framework and is not a certainty-of-evidence instrument.
RESULTS	16a	Describe results of the search and selection process, from records identified through studies included, ideally using a flow diagram.	pp. 7–8 – Figure 2 PRISMA 2020 flow diagram: 895 records identified, 136 duplicates removed, 759 screened, 105 full-text reports assessed, 14 excluded, and 91 studies included.
RESULTS	16b	Cite studies that might appear to meet the inclusion criteria but were excluded, and explain why they were excluded.	p. 12 – Table 4 summarises the 14 full-text exclusion categories; paper-level exclusion decisions and reasons are retained in the supplementary screening audit dataset (S1). Individual excluded studies are not cited in the main manuscript.
RESULTS	17	Cite each included study and present its characteristics.	pp. 22–40 – Study characteristics and comparative evidence are presented in the manuscript; Supplementary S2 provides the complete study-level extraction matrix for all 91 included studies.
RESULTS	18	Present assessments of risk of bias for each included study.	pp. 13–18 – D1–D8 framework, inter-rater agreement and evidence mapping; Supplementary S3 provides the study-level D1–D8 assessments.
RESULTS	19	For all outcomes, present results for each study and, where applicable, effect estimates and precision.	pp. 29–40 – Study-level task-specific performance metrics are presented where reported; heterogeneous metrics were retained rather than pooled across incompatible tasks.
RESULTS	20a	For each synthesis, briefly summarise characteristics and risk of bias among contributing studies.	pp. 19–20, 33–40 and 53–55 – Synthesis characteristics, methodological patterns and D1–D8 findings inform the cross-study

Section / Topic	Item	PRISMA 2020 checklist item	Location in updated manuscript
			interpretation.
RESULTS	20b	Present results of all statistical syntheses; if meta-analysis was performed, present summary estimates, precision and heterogeneity.	No statistical meta-analysis was conducted; qualitative/descriptive synthesis and evidence mapping were used.
RESULTS	20c	Present results of investigations of possible causes of heterogeneity conducted.	pp. 53–55 and 63–64 – Qualitative cross-study analysis of differences in datasets, tasks, metrics, validation, geographic/institutional settings and deployment contexts.
RESULTS	20d	Present results of sensitivity analyses conducted to assess robustness of synthesized results.	No formal sensitivity analysis was conducted/reported because of substantial heterogeneity.
RESULTS	21	Present assessments of risk of bias due to missing results for each synthesis assessed.	No formal reporting-bias assessment was conducted; this limitation is transparently stated.
RESULTS	22	Present assessments of certainty (or confidence) in the body of evidence for each outcome assessed.	No formal quantitative certainty-of-evidence assessment was conducted; D1–D8 was used for study-level methodological concerns rather than certainty.
DISCUSSION	23a	Provide a general interpretation of the results in the context of other evidence.	pp. 53–65 – Discussion and Conclusion interpret the synthesized evidence in relation to prior literature, methodological patterns, generalizability and application contexts.
DISCUSSION	23b	Discuss limitations of the evidence included in the review.	pp. 63–64 – Section 10.6; evidence heterogeneity, external validation, clinical evidence, methodological limitations and other evidence-base limitations.
DISCUSSION	23c	Discuss limitations of the review processes used.	pp. 63–64 – Section 10.6; search-source, language/full-text access, heterogeneity, lack of formal registration/protocol, and

Section / Topic	Item	PRISMA 2020 checklist item	Location in updated manuscript
			evidence-base limitations are discussed.
DISCUSSION	23d	Discuss implications of the results for practice, policy and future research.	pp. 42–52 and 53–65 – Research gaps, clinical translation, medication safety, regulation, privacy, equity, foundation models, edge AI and future research directions.
OTHER INFORMATION	24a	Provide registration information for the review, including register name and registration number, or state that the review was not registered.	p. 7 – Section 2.1; the review was not prospectively registered in PROSPERO or another public review registry.
OTHER INFORMATION	24b	Indicate where the review protocol can be accessed, or state that a protocol was not prepared.	p. 7 – Section 2.1; no formal review protocol was prepared.
OTHER INFORMATION	24c	Describe and explain any amendments to information provided at registration or in the protocol.	p. 7 – Section 2.1; no registered protocol or prospective registration was available, and no protocol amendments are reported.
OTHER INFORMATION	25	Describe sources of financial or non-financial support for the review and the role of funders or sponsors.	p. 65 – Funding statement: financial support from Vellore Institute of Technology (VIT); no external funding or grant was received.
OTHER INFORMATION	26	Declare any competing interests of review authors.	p. 65 – Conflict of Interest statement.
OTHER INFORMATION	27	Report which materials are publicly available and where, including data-collection forms, extracted data, analysis data, analytic code and other materials.	p. 65 – Supplemental Data and Data Availability Statement identify supplementary screening, extraction and D1–D8 assessment data and the PRISMA checklist. Analytic code is not specifically identified as publicly available.
